# Analysis of long-range chromatin contacts, compartments and looping between mouse embryonic stem cells, lens epithelium and lens fibers

**DOI:** 10.1186/s13072-024-00533-x

**Published:** 2024-04-20

**Authors:** Michael Camerino, William Chang, Ales Cvekl

**Affiliations:** 1 The Departments Genetics, Albert Einstein College of Medicine, NY10461 Bronx, USA; 2 Ophthalmology and Visual Sciences, Albert Einstein College of Medicine, NY10461 Bronx, USA

**Keywords:** Chromatin, CTCF, Differentiation, DNA looping, ES cells, Hi-C, lens, Pax6, Topologically associated domains

## Abstract

**Background:**

Nuclear organization of interphase chromosomes involves individual chromosome territories, “open” and “closed” chromatin compartments, topologically associated domains (TADs) and chromatin loops. The DNA- and RNA-binding transcription factor CTCF together with the cohesin complex serve as major organizers of chromatin architecture. Cellular differentiation is driven by temporally and spatially coordinated gene expression that requires chromatin changes of individual loci of various complexities. Lens differentiation represents an advantageous system to probe transcriptional mechanisms underlying tissue-specific gene expression including high transcriptional outputs of individual crystallin genes until the mature lens fiber cells degrade their nuclei.

**Results:**

Chromatin organization between mouse embryonic stem (ES) cells, newborn (P0.5) lens epithelium and fiber cells were analyzed using Hi-C. Localization of CTCF in both lens chromatins was determined by ChIP-seq and compared with ES cells. Quantitative analyses show major differences between number and size of TADs and chromatin loop size between these three cell types. In depth analyses show similarities between lens samples exemplified by overlaps between compartments A and B. Lens epithelium-specific CTCF peaks are found in mostly methylated genomic regions while lens fiber-specific and shared peaks occur mostly within unmethylated DNA regions. Major differences in TADs and loops are illustrated at the ~ 500 kb *Pax6* locus, encoding the critical lens regulatory transcription factor and within a larger ~ 15 Mb WAGR locus, containing *Pax6* and other loci linked to human congenital diseases. Lens and ES cell Hi-C data (TADs and loops) together with ATAC-seq, CTCF, H3K27ac, H3K27me3 and ENCODE *cis*-regulatory sites are shown in detail for the *Pax6*, *Sox1* and *Hif1a* loci, multiple crystallin genes and other important loci required for lens morphogenesis. The majority of crystallin loci are marked by unexpectedly high CTCF-binding across their transcribed regions.

**Conclusions:**

Our study has generated the first data on 3-dimensional (3D) nuclear organization in lens epithelium and lens fibers and directly compared these data with ES cells. These findings generate novel insights into lens-specific transcriptional gene control, open new research avenues to study transcriptional condensates in lens fiber cells, and enable studies of non-coding genetic variants linked to cataract and other lens and ocular abnormalities.

**Supplementary Information:**

The online version contains supplementary material available at 10.1186/s13072-024-00533-x.

## Introduction

Transcriptional regulation of individual genes is primarily mediated by sequence-specific DNA-binding transcription factors bound to promoters and distal enhancers, local recruitment of specific chromatin remodeling enzymes/complexes, and generation of specific combinations of local histone posttranslational modifications (PTMs) that either facilitate or inhibit formation of molecular interactions required for active transcription. These regulatory mechanisms operate in the context of cell type-specific 3D-organization of the nucleus [1–3]. DNA organization involves individual chromosome territories, transcriptionally active and inactive chromatin domains, TADs, and chromatin loops of various sizes [2]. Thus, chromatin folding is critical for compacting DNA within the nuclear space and provides platform for transcription coupled with RNA splicing, DNA replication, DNA repair, and regulation of chromosome structure and maintenance [1]. Phase separation to generate nuclear condensates is now considered as the driving force of chromatin folding [1–3].

Interphase chromosomes have discrete territories within the nucleus [4, 5]. It has been shown that active gene expression and chromatin state is correlated with positioning of individual loci within the nucleus. Active transcription primarily occurs towards the center, whereas transcriptionally inactive heterochromatin is positioned toward the nuclear periphery [6–8]. The major features of higher-order genome organization are driven by the functional status of the genome including distribution of molecular complexes regulating transcription and other processes in a self-organizing system. In turn, architectural features of the genome modulate its function [2]. A series of studies have shown that the genome organization is cell-type specific and directly linked to tissue-specific transcription [3, 9–12]. Recent studies based on chromatin structural modeling suggest fast chromatin dynamics including promoter-enhancer loops [13]. Studies focused on individual cell types during embryonic development are thus important for our understanding how chromatin structure relates to the genome function.

Regulation of long-range chromatin interactions is mediated by structural maintenance complexes (SMCs) including cohesin and condensin [14–16]. The multifunctional sequence-specific DNA-binding transcription factor CTCF is the most prominent protein defining boundaries of the extruded loops [17–20]. CTCF is comprised from centrally-located 11 zinc-fingers [21] with five (ZF3-7) and two (ZF1 and ZF10) of them involved in DNA and RNA binding, respectively [22–24]. Both the N- and C-terminal portions of CTCF contain intrinsically disordered regions (IDRs) [25]. Within the interphase chromosomes, TADs are formed at scale of several hundred kilo base pairs (kb) through loop-extrusion mechanism involving ATP-dependent molecular motor activity of the cohesin complex comprised of SMC1, SMC3, RAD21, and SCC3 subunits [26, 27]. Mapping of in vivo CTCF chromatin binding using ChIP-seq together with its gene loss-of-function studies revealed an unexpected complexity of downstream effects regarding promoter-enhancer interactions, chromatin looping and transcription, and variable dependence of these processes on both CTCF and cohesion [20, 28–31]. Super-resolution imaging revealed CTCF clusters of 4–8 molecules with approximately a quarter of them coupled with 3–15 cohesin molecules that are separated from RNA polymerase II clusters showing that cohesin and transcription have contrasting functions in CTCF clustering [32].

High-throughput chromosome conformation capture (Hi-C) employs a chromosome conformation capture (3 C) technique coupled with next generation DNA sequencing and is currently used to generate organizational maps of chromatin interactions of individual tissues [33, 34]. This approach allows for high-throughput and unbiased data analysis of chromatin organization at a resolution under 10 kb [35]. Hi-C allows detection of compartmentalization status defined as “active compartments A” and “inactive compartments B”, TADs, and inter/intra chromosomal interactions [36, 37]. Combined with CTCF DNA-binding data via ChIP-seq, ATAC-seq data and relevant histone PTMs, Hi-C proves to be a powerful tool in identifying novel candidate promoter-enhancer interactions [38–40].

Embryonic development generates over 400 different basic cell types in the mammalian body from a single fertilized egg [41]. Ocular lens is a unique avascular tissue comprised from two types of cells of common origin from the anterior pre-placodal ectoderm called lens epithelium and lens fibers [42–45]. The anterior portion of the lens is comprised of an epithelial cell layer. Epithelial cells at the equatorial zone divide, migrate and differentiate into secondary fiber cells forming outer layers of the lens fiber cell compartment. Within the lens fiber cell compartment, lens transparency requires formation of organelle free zone (OFZ) to prevent light scattering with major consequences for gene expression control [46]. Between E16.5 to E18.5 of mouse embryonic development, lens fiber cell denucleation includes changes of the nuclear shape and size reduction, chromatin condensation [47], transfer of nuclear proteins into the cytoplasm, up-regulation of lens-specific acidic DNase IIβ, phosphorylation of nuclear lamin A and C proteins by Cdk1, and culminating in abrupt disintegration of the individual nuclei within the centrally located “primary” lens fiber cell compartment [48–52]. Surprisingly, nascent crystallin gene expression remains at their maximal levels in these reorganizing nuclei [51]. At steady state levels, expression of crystallin genes ranks among the highest of any biological system found in nature, only comparable to globin genes in red blood cells [53]. Disrupted lens fiber cell denucleation results in both congenital and cortical cataracts [54–56].

A systematic analysis of the spatial and temporal organization of the genome is conducted by the four-dimensional nucleome (4DN) consortium [57]; however, no studies of the ocular cells are included. In the eye, earlier Hi-C studies analyzed mouse neural retina at different developmental stages [58], human adult neural retina [59] and human corneal limbal cells [60]. In the lens, temporal regulation of promoter-enhancer looping within the mouse αA-crystallin locus analyzed by 3 C also revealed two shadow enhancers [61]. To examine global 3D nuclear organization of the lens, we performed Hi-C and CTCF ChIP-seq using microdissected newborn mouse lenses and included ES cells for direct comparative analyses. Our earlier RNA-seq, ATAC-seq and whole genome bisulfite sequencing (WGBS) data from similar samples [62–64] serve for direct integration with nucleome mapping tools. ChIP-seq data including Pax6, RNA polymerase II and histone PTMs from newborn lens chromatin are also available [53, 65] for extended analyses. The main findings show local functional differences in nuclei from the lens epithelium compared to lens fibers and marked differences compared to ES cells and localization of majority of crystallin loci outside of chromatin loops.

## Results

### Hi-C sequencing identifies chromatin reorganization in differentiating lens

**Fig. 1:**
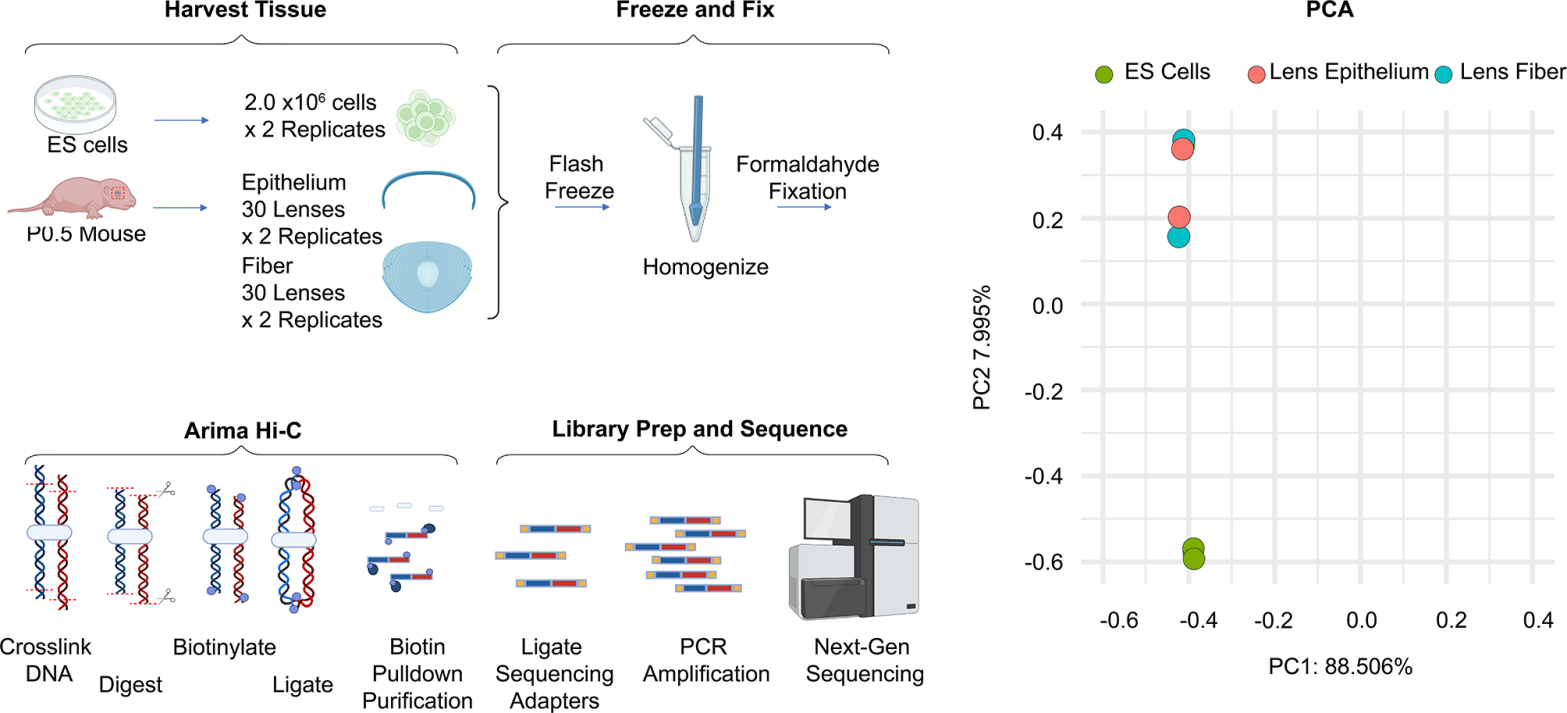
Tissues and experimental design of the Hi-C study. Lenses were harvested from newborn (P0.5) mice and microdissected into epithelium and fiber cells (30 lenses x 2 replicates). Mouse ES cells were harvested near ~ 80% confluency (2.0 × 106 cells x 2 replicates). Cells were crosslinked and processed according to Arima’s library preparation protocol. Chromatin contact maps were generated using the ENCODE pipeline. Differential compartment A/B analysis was performed using dcHiC (see Materials and Methods). The principal component analysis (PCA) on compartmental eigenvectors shows that both lens cells are distinct from the ES cells

To examine nucleome dynamics between the newborn mouse lens epithelial and fiber cells, we employed deep Hi-C sequencing. Parallel studies included mouse ES cells as a reference as it represents “ground” state of embryonic development and chromatin organization [9, 66]. A schematic of project workflow is shown in Fig. 1. To elucidate lens cell-type specific changes in chromosome organization, we initially investigated chromatin looping, TADs, compartments A and B, and chromatin state. With a total of 4.55 billion read pairs generated in ES cells, lens epithelium and lens fiber cells (1.48, 1.57, 1.50 billion read pairs, respectively), we detected 1.93 billion Hi-C contacts (Additional File 1: Table S1). Combining both biological replicates of ES cells, lens epithelium and lens fiber cells generated similar total number of 643.2, 588.0, and 699.1 million individual Hi-C contacts, respectively. The ES cells showed a far lower proportion of inter-chromosomal contacts (10.8%) compared to both lens epithelium (24.1%) and lens fiber cells (32.2%) (Additional File 1: Table S1). To determine the predicted resolution of the contact maps we used HiCRes [67]. When filtering out reads with MAPQ < 30 ESCs, lens epithelium, and lens fiber cells, all had a similar predicted resolution of 2.69–3.32 kb using 545–595 million read pairs (Additional File 2: Fig. S1). For A/B compartment analysis, dcHiC procure was used [68]. As expected, principal component analysis (PCA) on A/B compartment status shows both lens cell types clustered together and separated from ES cells (Fig. 1).

### Large scale changes in chromatin domains during lens cell differentiation

**Fig. 2:**
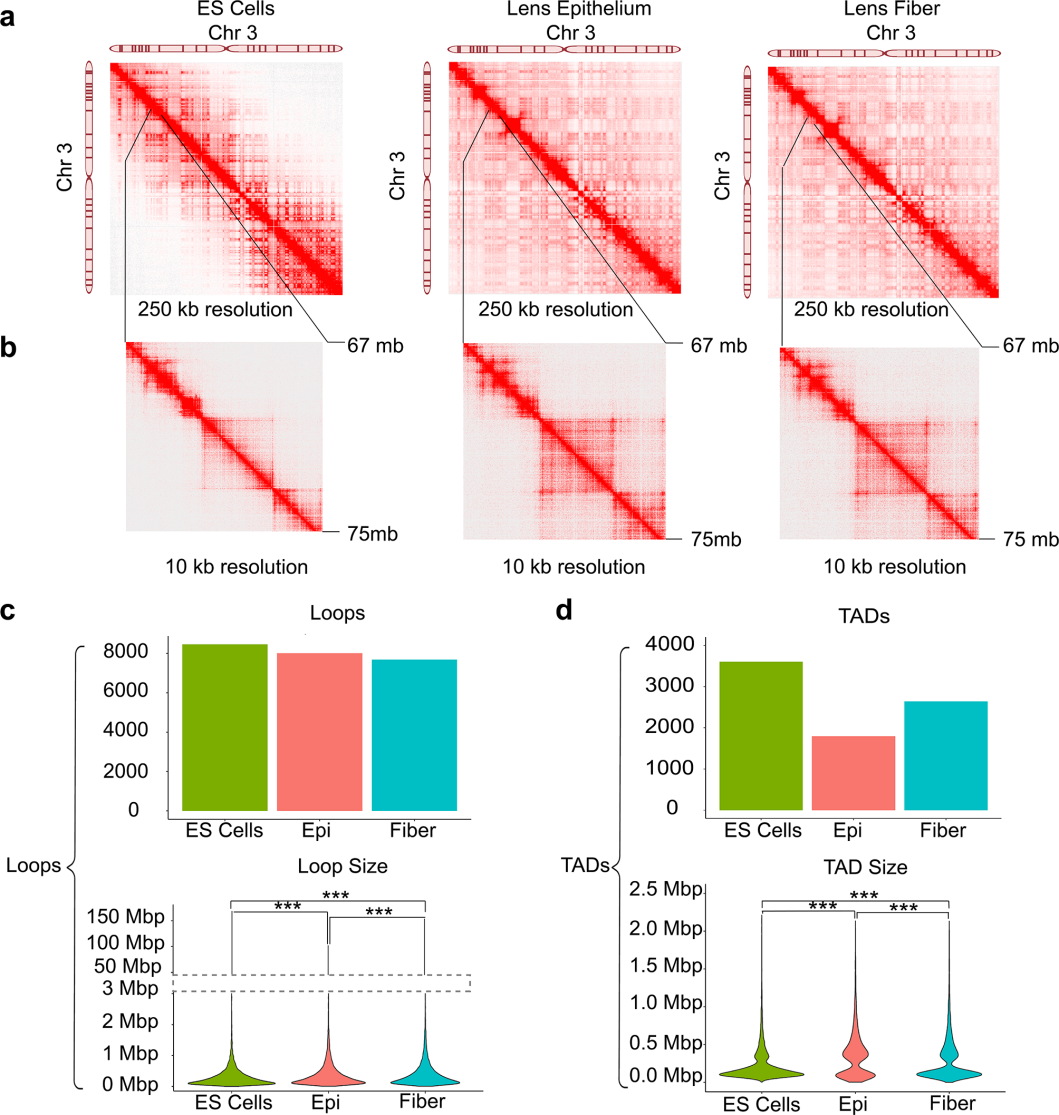
Mapping of chromatin loops and TADs in ES and lens cells. **(a)** Interaction maps of chromosome 3 generated from ES, lens epithelium and lens fiber cells. Interactions shown are over the span of ~ 160 Mb and are binned at 250 kb resolution. **(b)** Magnified view of a representative segment of 67–75 Mb on chromosome 3 showing marked differences in chromosome interactions between ESCs and lens cells. Interactions shown are binned at 10 kb resolution. **(c)** Distribution of loop sizes called in ES, lens epithelium and lens fiber cells, respectively. Dashed lines connect scale ranges to show all data points. **(d)** Number of TADs called in ES, lens epithelium and lens fiber cells including their median TAD sizes: 145, 135 and 335 kb, respectively. Significance bars denoted with asterisks: *p* ≤ 0.05, *p* ≤ 0.01 and *p* ≤ 0.001 denoted as *, **, *** respectively

First, to demonstrate differences between ES cells and both lens cells at the chromosomal (250 kb resolution) and the locus level (10 kb resolution), interaction maps of chromosome 3 are displayed in Fig. 2a. For example, there are notable differences in contact density and long-range chromatin interactions (denoted by arrows) such as within the chr3: 67,000,000–75,000,000 regions between the ES and both lens cells (Fig. 2b).

**Fig. 3:**
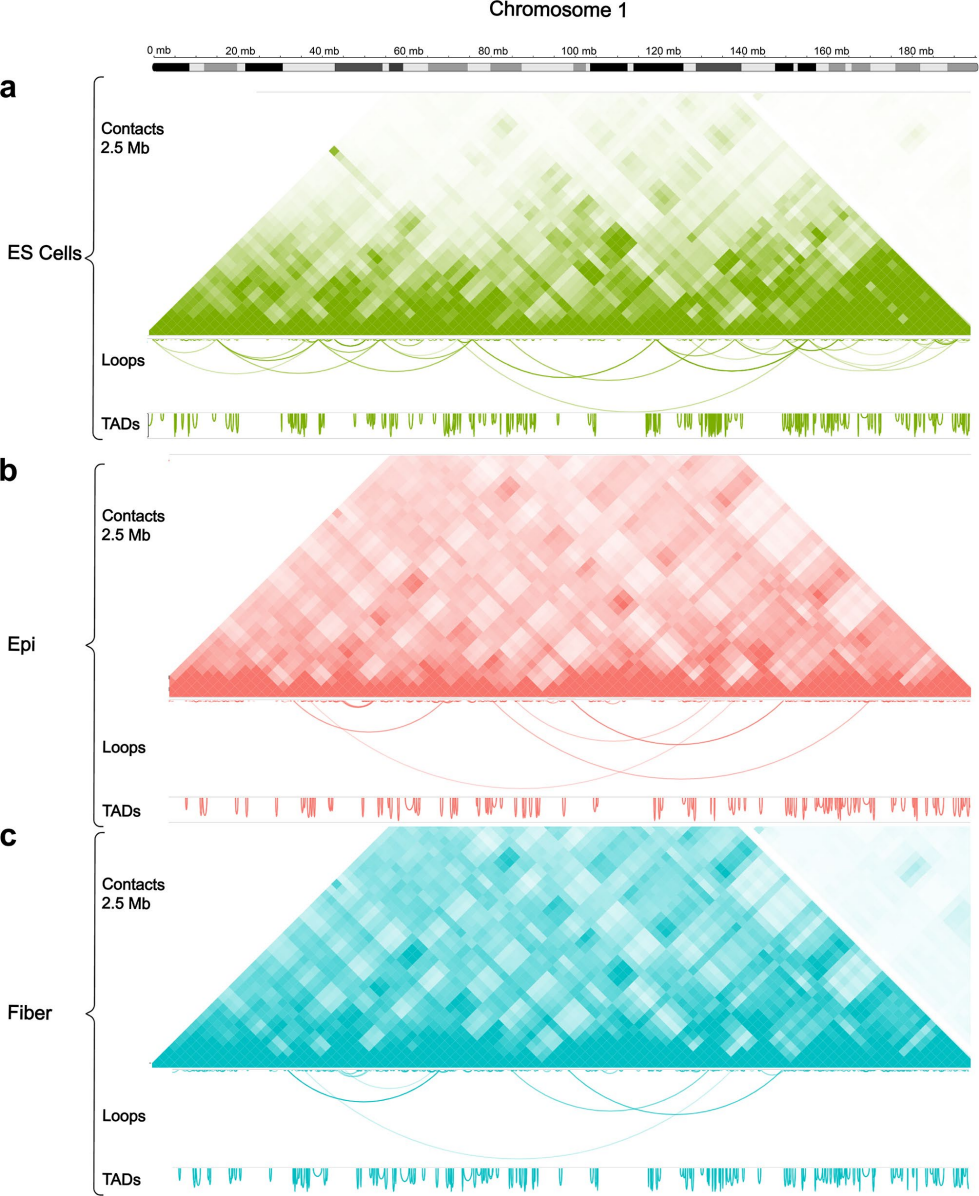
Differences between the number and distribution of long-range chromatin loops pertinent to entire chromosome 1 in ES and lens cells. Chromatin contact map showing TAD and loop size distribution along chromosome 1 in **(a)** ES cells (572 loops and 232 TADs), **(b)** Lens epithelium (569 loops and 127 TADs), and **(c)** Lens fiber cells (533 loops and 187 TADs). Contact interactions are binned at 2.5 Mb resolution. Genome wide loop calling shows larger number of loops > 3 Mb in ES (*n* = 315) compared to both lens epithelium (*n* = 84) and lens fiber cells (*n* = 82)

Next, we performed comparative analyses of chromatin loops and TADs. While similar number of loops were found between ES cells (*n* = 8,459), lens epithelium (*n* = 8,005) and lens fiber cells (*n* = 7,682), loop sizes and their distribution varied greatly. ES cells had a significantly higher mean loop size (989 kb) compared to the lens epithelium (582 kb) and lens fiber cells (581 kb) but all shared similar median sizes (220, 250 and 269 kb, respectively), indicating a higher proportion of long-range loop formations in ES cells (Fig. 2c). This is consistent with previous studies, showing that these interactions are reorganized after individual cell-fate specific differentiation programs [69–71]. ES cells had the largest number of TADs (*n* = 3,604) compared to both lens epithelium (*n* = 1,796) and lens fiber cells (*n* = 2,641). Interestingly, ES and lens fiber cells shared similar median TAD sizes (145 and 135 kb, respectively), whereas lens epithelium median TAD size was more than 2-fold larger (335 kb), indicating reorganization of the nucleome during lens epithelial to fiber cell transition (Fig. 2d). Cell type-specific chromatin loop and TAD size trends are mostly consistent across each individual 20 chromosomes (Additional File 2: Fig. S2). Next, chromosome 1 is shown to illustrate the difference in long-range loop formations in ESCs (Fig. 3a) compared to both lens cells (Fig. 3b-c). Genome wide loop calling shows a near 4-fold difference in chromatin loop size > 3 Mb in ES cells (*n* = 315) compared to both lens epithelium (*n* = 84) and lens fiber cells (*n* = 82). For statistical analysis of genome wide loop and TADs for every chromosome, see Additional File 7: Table S2. Though loop size distributions vary between ES and lens cells, genome wide loop anchor annotations show similar proportions of contacts mapped to exons, intergenic regions, introns and promoters (Additional File 2: Fig. S3).

**Fig. 4:**
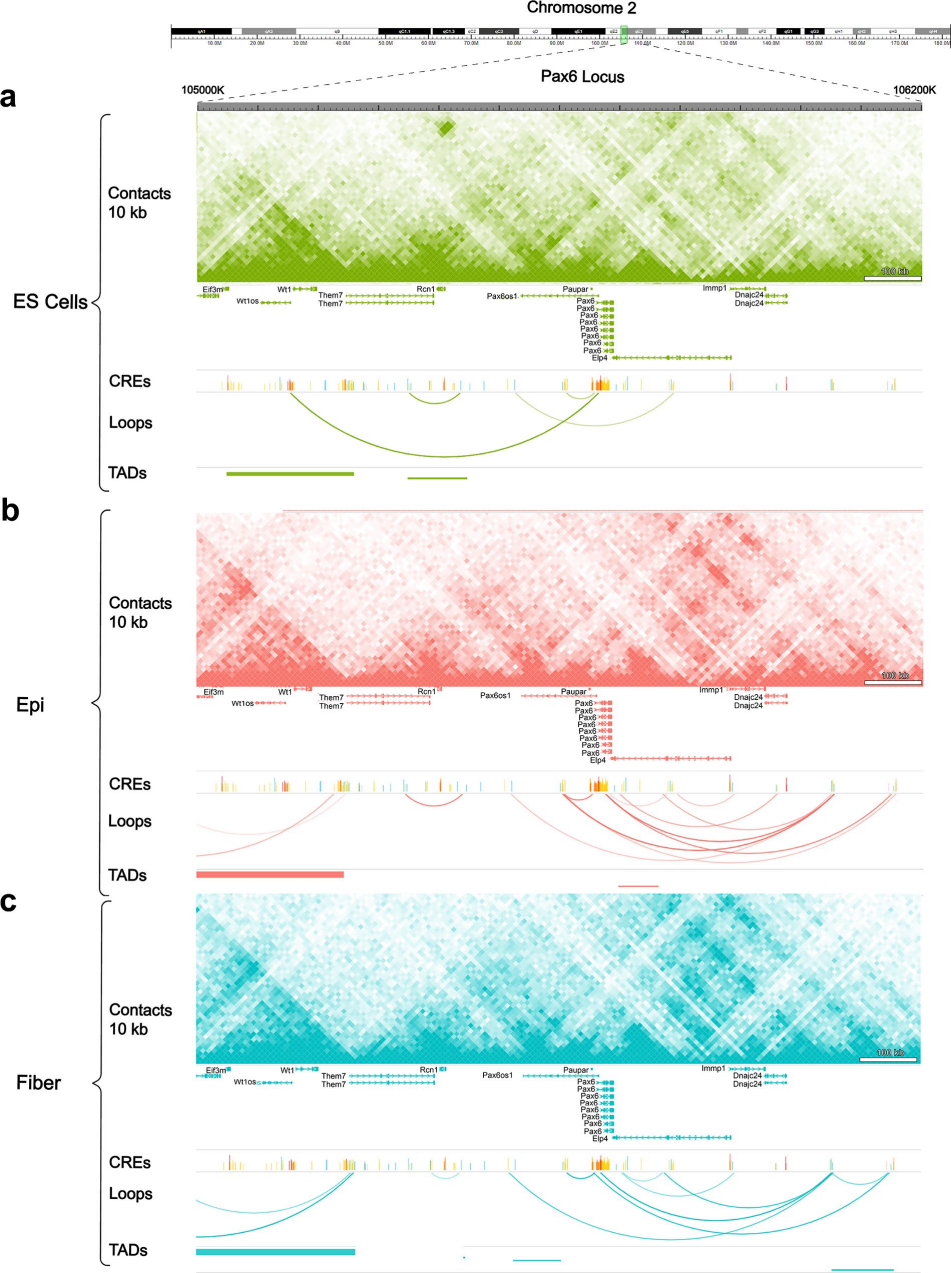
Identification of chromatin loops and TADs at the representative *Pax6* locus. Loops and TADs at the *Pax6* locus (1.4 Mb DNA region of the chromosome 2) show markedly different chromatin interactions in ES compared to both lens cells. **(a)** The *Pax6* locus within the larger WAGR region in ES cells shows long-range chromatin looping between the *Pax6* and *Wt1* loci while very limited activity within the *Pax6* locus. Chromatin looping patterns within the *Pax6* locus are mostly shared between **(b)** lens epithelium and **(c)** lens fiber cells. Contact interactions are binned at 10 kb resolution

Given the most prominent role of DNA-binding transcription factor Pax6 in lens progenitor lens cell formation, separation of the lens vesicle from the surface ectoderm and formation of lens epithelium and lens fibers [72, 73], we used the ~ 500 kb *Pax6* locus as the model to visualize individual chromatin loops. Importantly, human PAX6 locus is located within a larger 7 Mb genomic WAGR (Wilms tumor, aniridia, genitourinary malformation and mental retardation syndromes) region, where large deletions and mutations including *WT1*, *RCN1*, *PAX6*, *PAX6OS1* and *ELP4* loci cause interrelated human diseases affecting kidney, eye, brain and genitals [74, 75]. Here we identified one notable long-range interactions between the mouse Pax6 promoter region with *Wt1* (~ 540 kb, Fig. 4a) and *Meis2* (~ 10.3 Mb, data not shown) within ES cells that was not found in lens cells (Fig. 4b-c). Interestingly, *Meis2* encodes another transcription factor directly regulating Pax6 expression via multiple distal enhancers [76]. These interactions are in agreement with other ES Hi-C data sets [17, 66]. As expected, the *Pax6* locus shows dramatic changes in chromatin reorganization in both lens epithelium (Fig. 4b) and lens fiber cells (Fig. 4c), making contacts with both up- and down-stream proximal and distal enhancers when compared to ES cells. Given the complexity of DNA loops found in these three cell types, expression levels of WAGR genes as well as Pax6os1, Paupar and Wt1os lncRNAs in lens epithelium and fibers are shown in Additional File 2: Fig. S4. Thus, analyses of individual model loci reveal new insights into long-range chromatin loops that can be linked to cell-specific transcription (see below).

### Inter/intra-loops, TADs, type of chromatin loops, transcription, and compartment A/B analyses

**Fig. 5:**
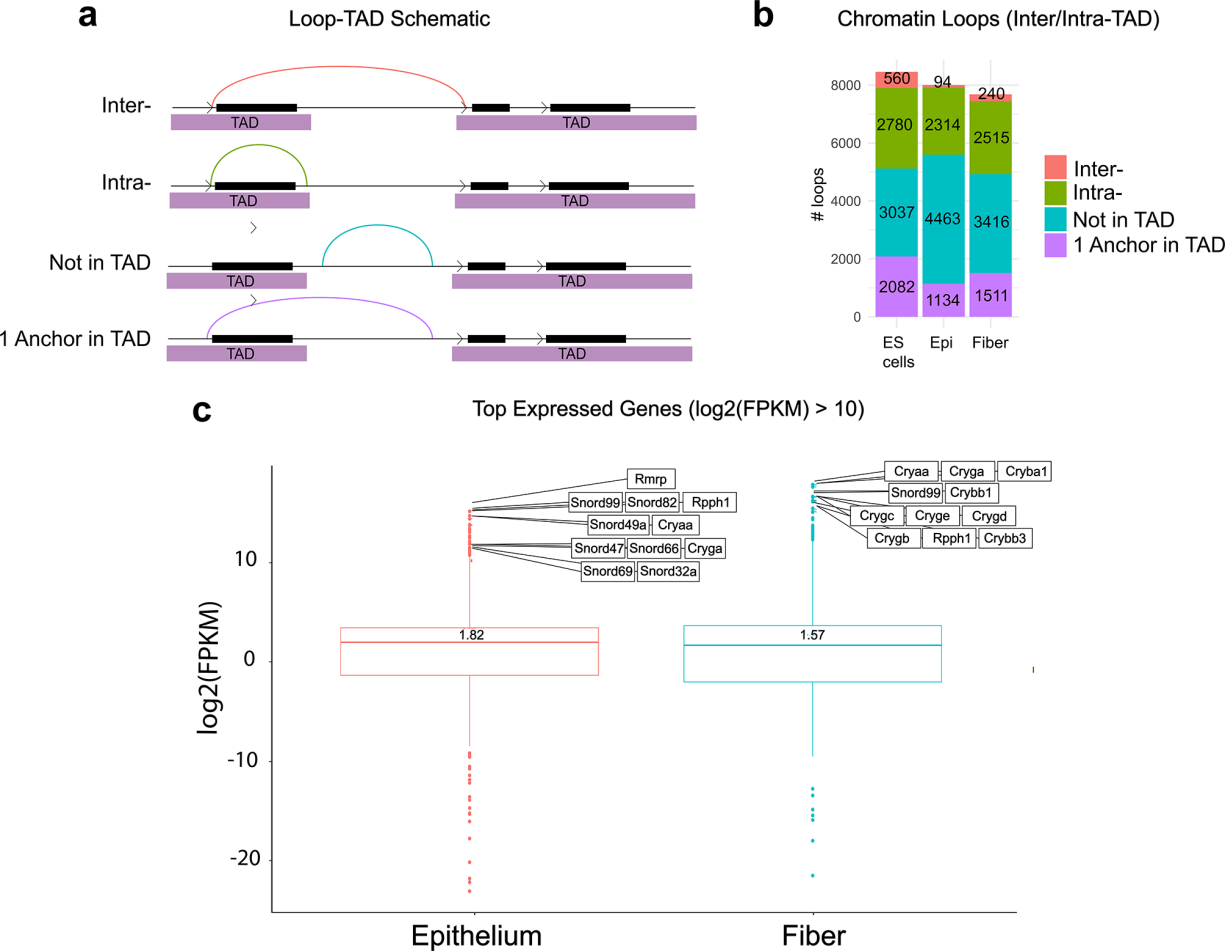
Proportion of inter-/intra-TAD chromatin loops and distribution of RNA expression of individual genes in lens cells. **(a)** Schematic diagram showing four possible loop-TAD anchor arrangements. Inter- is defined where both loop anchors connect two separate TADs. Intra- is defined where both loop anchors are within a single TAD. Loops with anchors that were found between TADs were defined as “Not in TAD”. Loops where one anchor lies within a given TAD and the other lies outside of the TAD were defined as “1 anchor in TAD”. **(b)** Quantitative analysis of four types of chromatin loops. ES cells show a higher number of Inter-TAD chromatin loops (*n* = 560) than lens epithelium (*n* = 94) and fiber cells (*n* = 240). Lens epithelial cells show the highest number of loops where anchors are not within a TAD boundary (*n* = 4,463). **(c)** Boxplot showing top differentially expressed coding and non-coding transcripts with fragments per kilobase of transcript per million mapped reads (FPKM). The top expressed genes in the lens epithelium mostly include small nuclear RNAs and αA-crystallin (*Cryaa)* gene whereas lens fiber cells show high expression of multiple crystallin genes

Next, we examined the relationship between loop anchors and TADs using Juicer tools HiCCUPS and Arrowhead, respectively [77]. We grouped the possible arrangements into four categories: Inter- and intra-loops, loops outside of TAD, and one loop anchor in TAD as schematically shown in Fig. 5a. ES cells showed a higher number of Inter-TAD chromatin loops (*n* = 560) compared to both lens epithelium (*n* = 94) and fiber cells (*n* = 240). Lens epithelial cells show the highest number of loops where anchors were not within a TAD boundary (*n* = 4,463) (Fig. 5b). Taken together, these data show major chromatin remodeling in the pathway from ES to lens cells.

Lens cells are characterized by robust expression of genes encoding α-, β- and γ-crystallins [53, 64]. Thus, we next analyzed gene expression profiles of newborn lens epithelium and lens fiber cells for comparative analysis with A/B compartment status. Transcriptome profiling reveals that lens epithelium has enrichment for *Cryaa* and *Cryga* crystallin genes and a battery of small-nuclear non-coding RNAs. Lens fiber cells show higher expression (Log2(FPKM) > 10.0) of lens structural genes including five members of the γ-crystalin gene family (Cryga, Crygc, Cryge, Crygd and Crygb), as well as members the β-crystalin family (Cryba1, Crybb1 and Crybb3) (Fig. 5c). Expression of all genes in lens epithelium and lens fiber cells can be found in Additional File 8: Table S3 and Additional File 9: Table S4, respectively.

**Fig. 6:**
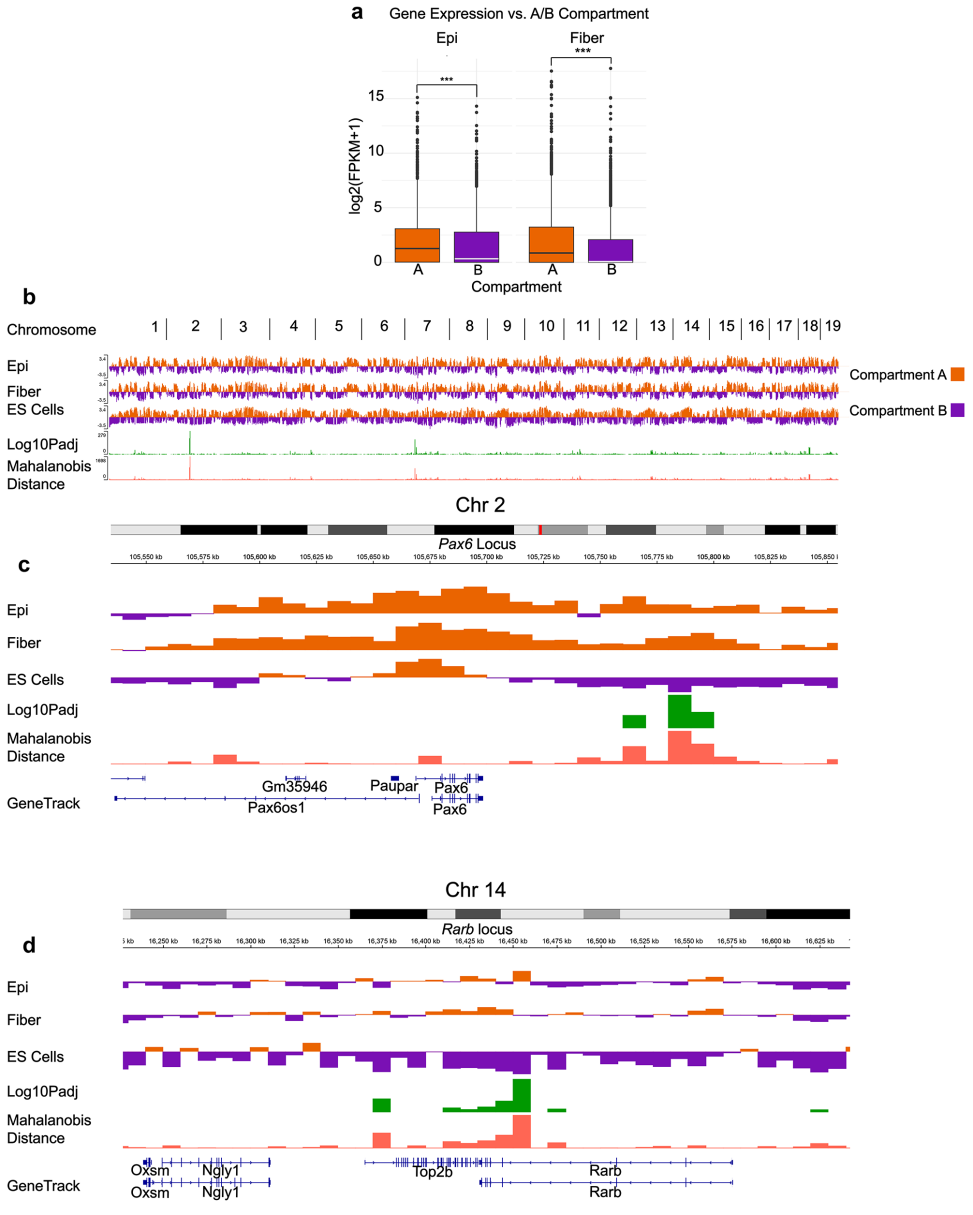
Comparative analysis of compartments A/B between both lens and ES cells. **(a)** Median gene expression of genes in A/B compartments in of lens epithelial (median: A = 1.26, B = 0.332) and lens fiber cells (median: A = 0.854, B = 0.054). **(b)** Genome wide compartment analysis. Two *Pax6* **(c)** and *Rarb* **(d)** loci are used to illustrate regional compartmental changes. Positive Eigen values shown in orange are denoted as “Compartment A”, whereas negative Eigen values shown in purple are denoted as “Compartment B”. Green peaks are show significant differences in A/B compartmental changes between cell types (Log 10 P adjusted of significant Mahalanobis peaks)

Additional global analyses of steady-levels of individual RNAs found within compartments A and B are shown in Fig. 6. When cross-referencing transcriptional data with compartment status, we found that both lens epithelium and fiber cells had higher median gene expression levels in compartment A (1.26, 0.854) than in compartment B (0.332, 0.054), respectively (Fig. 6a). Overall, genome wide compartment analysis shows minor differences between lens cell types but marked differences between lens cells and ESCs. The significant differences are denoted by Log10P adjusted values, notably at individual chromosomes 1, 2, 4, 7 11, 13, 14, 18, and 19 (Fig. 6b). We also noted locus specific compartmental changes. For representative loci, the *Pax6* locus introduced above exhibits dramatic changes from compartment B to A downstream of the gene body between ES to lens cells. Pax6 regulates αB- and γF-crystallin gene expression via synergistic action with RARβ/RXRβ heterodimeric transcription factors [78, 79]. The *Rarb* locus (novel cataract risk locus) [80], chromosome 14) also shows compartmental changes within the gene body (Fig. 6c-d). Finally, GO analysis of significant A/B compartment changes (500 unique genes) showed enrichment for terms related to cell morphogenesis, positive regulation of Ras protein signal transduction, homophilic cell adhesion, and response to leukemia inhibitory factor (Additional File 10: Table S5).

**Fig. 7:**
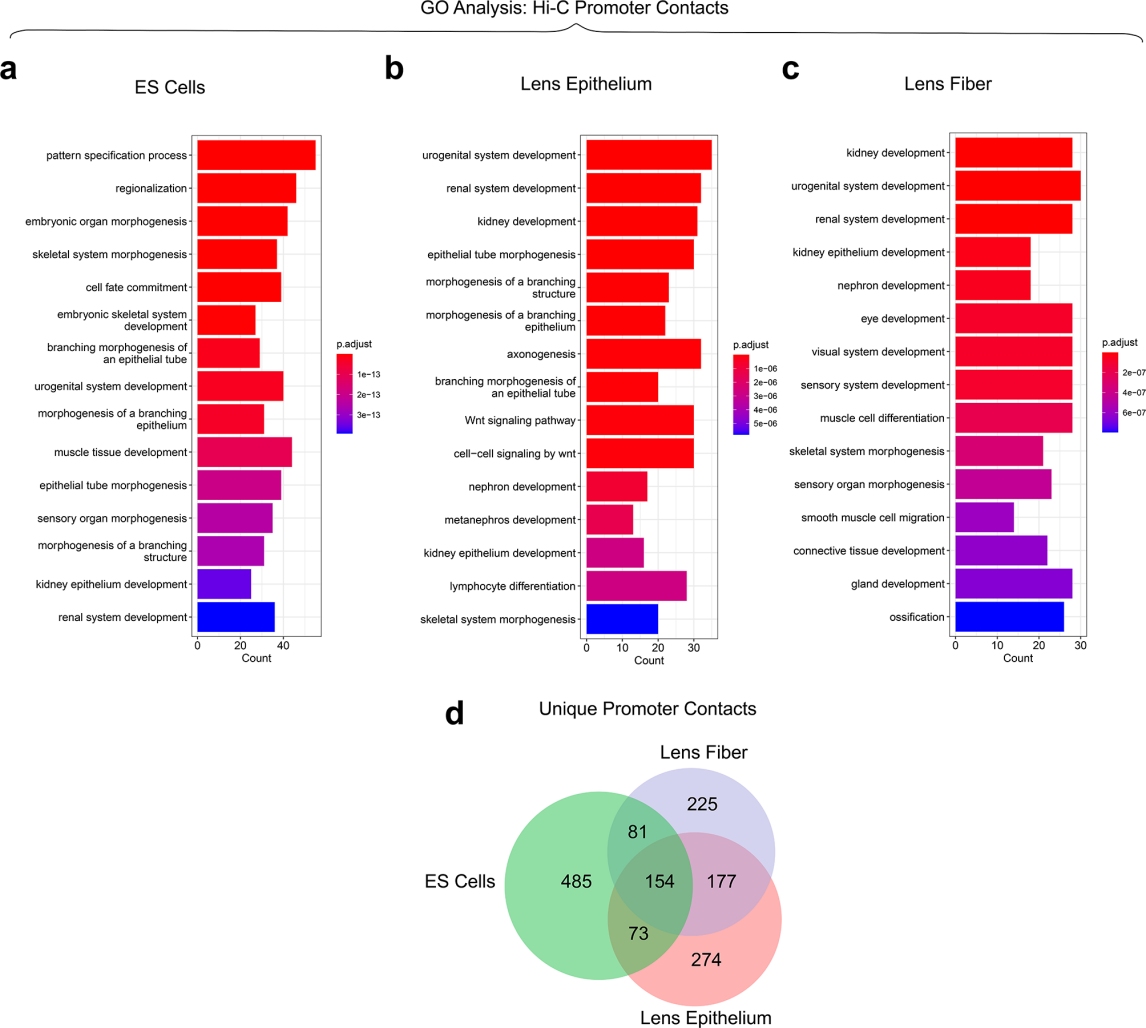
Gene Ontology (GO) analysis of Hi-C promoter chromatin contacts. GO analysis of chromatin loop contacts at or near promoters (2 kb upstream and 0.1 kb downstream of transcriptional start sites). **(a)** GO analysis of promoter contacts in ES cells. Note enriched terms related to embryonic development and cell fate commitment. **(b)** GO analysis of promoter contacts in lens epithelium. Note enriched terms related to epithelial morphogenesis, differentiation and proliferation. **(c)** GO analysis of promoter contacts in lens fiber cell promoter contacts. Note enriched terms related to development of the visual system. **(d)** Lens epithelium and fiber cells share more promoter contacts (*n* = 331) than with ES cells (*n* = 227 and *n* = 235, respectively). ES cells had the highest number of unique promoter contacts (*n* = 485) when compared with lens epithelium (*n* = 274) and lens fiber cells (*n* = 225)

To further understand functional roles of genes involved in chromatin looping, we performed GO analysis on loop anchors found within 1 kb upstream and 100 bp downstream of the transcriptional start site (TSS). Promoter contacts from ES cells show high enrichment for biological processes related to embryonic development. Notable terms (Fig. 7a) include pattern specification process (*p* = 3.27 × 10^− 21^), embryonic organ morphogenesis (*p* = 4.45 × 10^− 18^), and cell fate commitment (*p* = 1.92 × 10^− 17^). Notable genes with promoter looping include structural development genes *Runx2*, *Tbx1* and *Lhx1* (Additional File 11: Table S6); however, no data exists on their roles in lens. There was also promoter looping in important genes related to cell signaling and patterning, including *Wnt5a* [81, 82] and *Shh* [83, 84] (Additional File 11: Table S6), both genes involved in lens development. In lens epithelium, notable GO enrichments were found for terms related to epithelial development including epithelial tube morphogenesis (*p* = 8.61 × 10^− 9^), morphogenesis of a branching epithelium (*p* = 5.21 × 10^− 11^), and Wnt based cell-cell signaling (*p* = 1.98 × 10^− 10^) (Fig. 7b). Some prominent genes related to epithelial development with promoter looping include *Bmp4*, *Rara*, and *Pax2* [85–87] (Additional File 11: Table S6). There were also genes related to epithelial cell migration (*p* = 5.36 × 10^− 4^) including *Pxn* and *Hdac7* [88, 89] with promoter looping. Eye development was also significant GO category (*p* = 4.73 × 10^− 4^), listing 21 genes with promoter looping (Additional File 11: Table S6). Finally, lens fiber cell promoter contacts had notable GO enrichment for eye related terms including eye development (*p* = 8.08 × 10^− 8^), visual system development (*p* = 8.14 × 10^− 8^), and sensory system development (*p* = 8.82 × 10^− 8^) (Fig. 7c).

Notable genes encoding transcription factors regulating lens morphogenesis found here with promoter looping include *Pax6* [76], *Meis2* [76] and *Sox1* [90]. Lens fiber cell promoter contacts were also found in *Fgfr2* and *Rara* loci encoding proteins involved in FGF [91–93] and retinoic acid [94] signaling during lens development. Genes encoding anti-apoptotic protein Bcl2 [95] and chromosome organizational protein Nipbl involved in cohesion activities [96] were also noted for their lens fiber-specific promoter looping. Lens epithelium and lens fiber both share a similar number of unique promoter contacts (*n* = 274 and *n* = 225, respectively), while ES cells had the highest number (*n* = 485). Lens epithelium and fiber cells share more promoter contacts (*n* = 331) and with ES cells (*n* = 227 and *n* = 235, respectively) (Fig. 7d).

### Lens epithelium and fiber cells show different CTCF-binding associated with distinct CTCF-anchored looping distributions, but common DNA methylation patterns

**Fig. 8:**
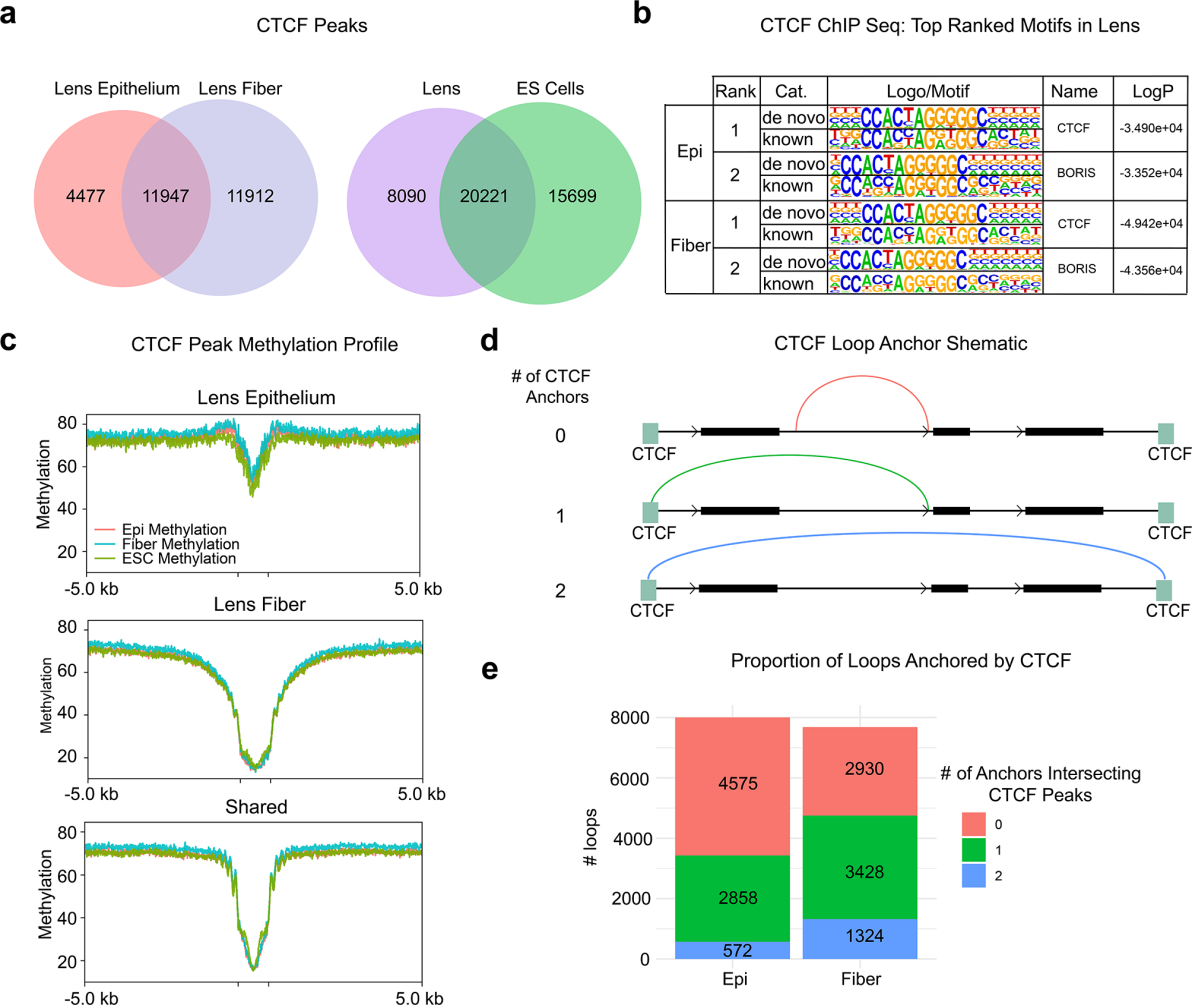
CTCF binding in lens epithelium and fibers and ES cells, role of DNA methylation and different CTCF-anchored looping distributions. **(a)** Two comparisons between total number of peaks identified in lens epithelium and fiber cells with 11,947of sheared CTCF peaks. **(b)** Identification of the top enriched motifs with the peaks and their direct comparison with known CTCF binding motifs. **(c)** CTCF peaks and DNA methylation (epi, fiber, and ES cells) in lens epithelium-specific, fiber-specific and shared peaks. Shown are centers of the peaks and their 5 kb flanking regions. **(d)** Proportions of loops anchored by CTCF. Three possible loop-CTCF anchor arrangements are shown. Note gain of total numbers of peaks with one or two anchors in lens fiber cells

To further examine chromatin organization in both lens cells, we determined binding of CTCF by ChIP-seq. A total number of individual 16,424 and 23,859 CTCF peaks were found in lens epithelium and lens fiber cells, respectively (Fig. 8a). These numbers are within the similar range as results obtained in mouse retina and brain [97, 98]. Between the two lens cell types, 11,947 (42.2%) of CTCF peaks are shared (Fig. 8a). To compare lens CTCF peaks with ES cells we compared the present data with an earlier study that by Casellas lab [99]. We then aggregated all lens CTCF peaks and found that lens cells have 8,090 (18.4%) unique and 20,221 (45.9%) shared peaks with ES cells (Fig. 8a). DNA *cis*-motif analysis of the individual peaks revealed that both lens epithelium and lens fiber cells shared the same top ranked motifs aligned with CTCF and BORIS aka CTCFL consensus binding sites (Fig. 8b). Note that BORIS is a germline-specific paralogue of CTCF [19, 100].

Earlier studies have shown that DNA methylation regulates binding of CTCF to DNA, impacting 3D genome structure and gene regulation [24, 101, 102]. Next, we used our recent WGBS data for newborn lenses and ES cells [63] to examine methylation patterns within the individual lens CTCF peaks. Overall, demethylation was observed in both lens epithelium and fiber at loci corresponding to epithelium-specific, fiber-specific, and shared CTCF peaks, though this demethylation was less pronounced at epithelium-specific peaks. Intriguingly, differential CTCF binding between lens epithelium and fiber was not associated with differential demethylation; instead, all DNA methylation patterns were similar between epithelium, fiber, and ES cells at epithelium-specific, fiber-specific, and shared CTCF peaks. This suggests that, while DNA demethylation may be necessary for CTCF-binding, it is not sufficient for predicting cell-specific CTCF (Fig. 8c).

Finally, to understand loop organization in the context of CTCF binding we analyzed proportion of loops containing 0, 1, or 2 anchors bound by CTCF (Fig. 8d). We found that lens fiber cells had nearly a ~ 2.5-fold higher proportion of loops (17.2%) where both loop anchors were bound by CTCF when compared to lens epithelium (7.15%). In contrast, lens epithelial cells have the highest proportion of loops (57.2%) where either anchor was not bound by CTCF when compared to lens fiber (38.1%).

### Subnuclear localization and changes in CTCF in differentiating lens fibers

**Fig. 9:**
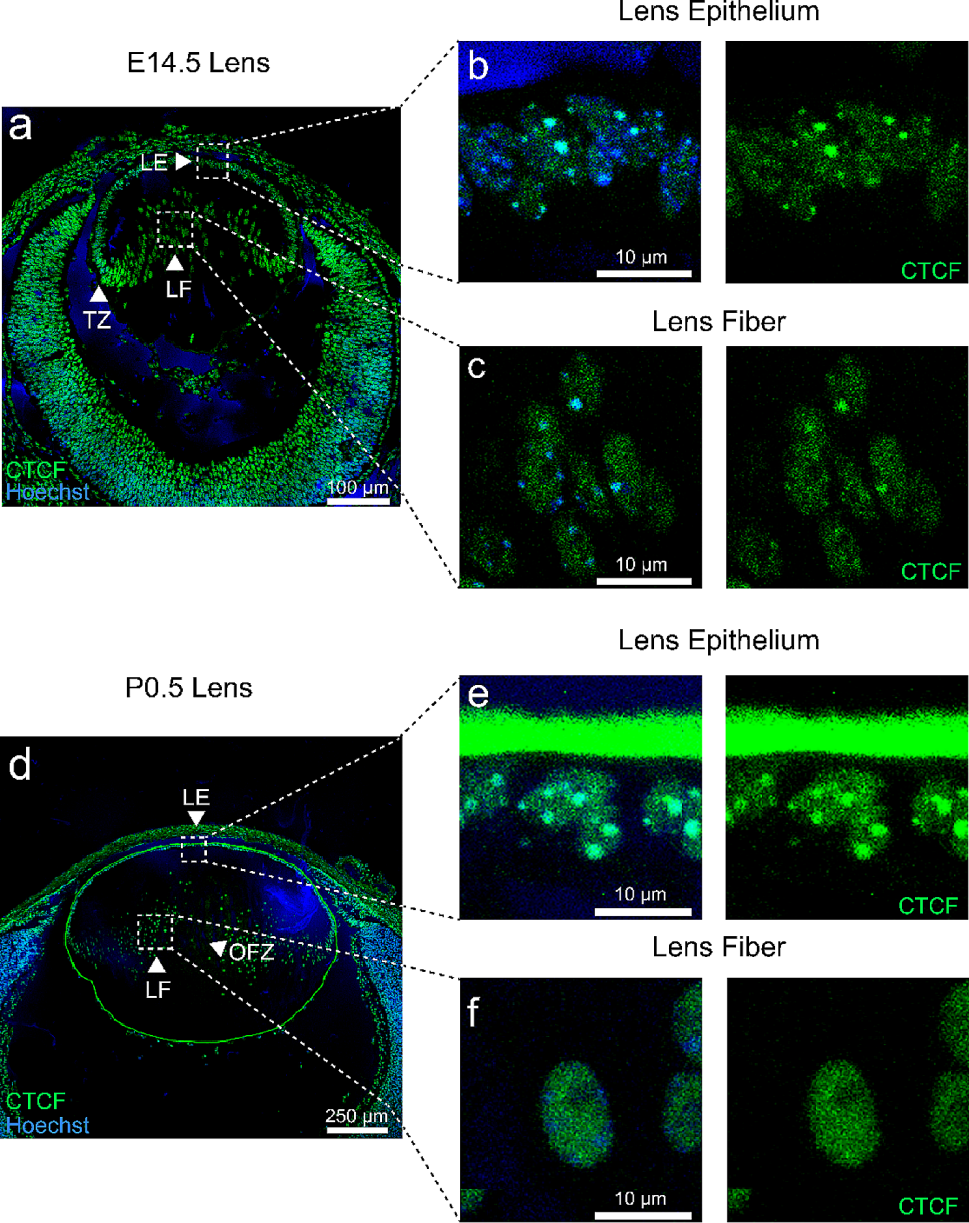
Immunofluorescence analysis of CTCF in developing mouse lens. **(a)** E14.5 mouse lens labeled for CTCF and nuclear stain Hoechst showing lens epithelium (LE) and primary lens fiber (LF) elongation and secondary LF formation at the transition zone (TZ). **(b)** Magnification of lens epithelium nuclei showing localization of CTCF in both nucleoplasm and nucleoli. **(c)** Early primary lens fiber cells also show localization of CTCF in nucleoli, but more diffusion in the nucleoplasm. **(d)** P0.5 lens showing mature structure of the lens and formation of the organelle-free zone (OFZ) as primary lens fiber cells are already denucleated. **(e)** Lens epithelial cells show strong CTCF signal in nucleoli and staining across the nucleoplasm. **(f)** Primary and secondary fiber cells show translocation of CTCF to the lens nucleoplasm prior their denucleation

To evaluate potential changes in CTCF subnuclear localization during lens development we performed immunofluorescence analyses of E14.5 and P0.5 mouse lenses. By E14.5, the lens epithelium and fiber cell compartments are fully formed and primary lens fibers execute their terminal differentiation. Lens epithelial cells located at the lens equator exit the cell cycle and differentiate into the secondary lens fibers [72] (Fig. 9a).

At E14.5 central and peripheral lens epithelium, distinct puncta of CTCF are localized in both nucleoplasm and nucleoli (Fig. 9b). Likewise, similar CTCF staining patterns are found in both differentiating primary and secondary lens fibers (Fig. 9c). At P0.5, primary lens fibers undergo their denucleation and the OFZ is formed (Fig. 9d). Both central and peripheral lens epithelium showed similar patterns of CTCF localization within both nucleoplasm and nucleoli (Fig. 9e). In contrast, the P0.5 fiber cells showed localization of CTCF to the nucleoplasm with much weaker CTCF staining within the nucleoli (Fig. 9f). Taken together, these findings demonstrate changes in CTCF subnuclear localization during lens fiber cell differentiation.

### Looping structures, CTCF binding and other chromatin features at individual model loci

**Fig. 10:**
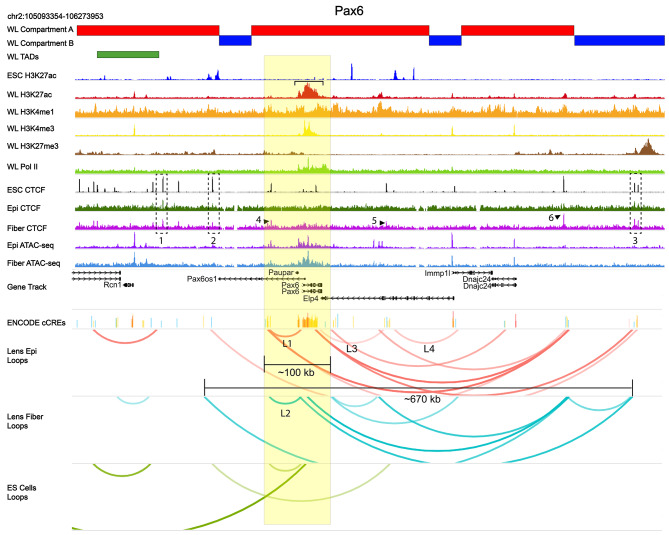
Chromatin loops, CTCF binding and other features of the *Pax6* locus. Genome browser representation of the loops (shown as arches) found at the *Pax6* locus and its flanking regions in lens epithelium (red), lens fibers (blue) and ES (green) cells. Pax6 coding regions including both proximal 5’- and 3’-flanking non-coding regions are highlighted in yellow. Compartments A and B are shown by red and blue horizontal bars, respectively. TAD regions identified by “Arrowhead” are shown in green. Whole lens ChIP-seq (H3K27ac, H3K4me1, H3K4me3 and H3K27me3 [59] and RNA polymerase II (Pol II) [47] tracks using whole lens (WL) chromatin are also shown. A broad region of H3K27ac is marked by a horizontal bracket. Lens epithelium (epi) and fiber cell CTCF (present study), ES cell CTCF [66], and ATAC-seq [56] are shown together with ENCODE candidate *cis*-regulatory elements (cCREs): Promoter-like signatures (red), proximal enhancer-like signature (orange), distal enhancer-like signature (yellow), DNase-H3K4me3 (pink) and CTCF (blue). Specific CTCF peaks and loops discussed in the text are marked by dotted boxes (1–3), numbered arrows (4–6) and (L1-L4), respectively. Yellow box is used to indicate locus of interest and scale.

**Fig. 11:**
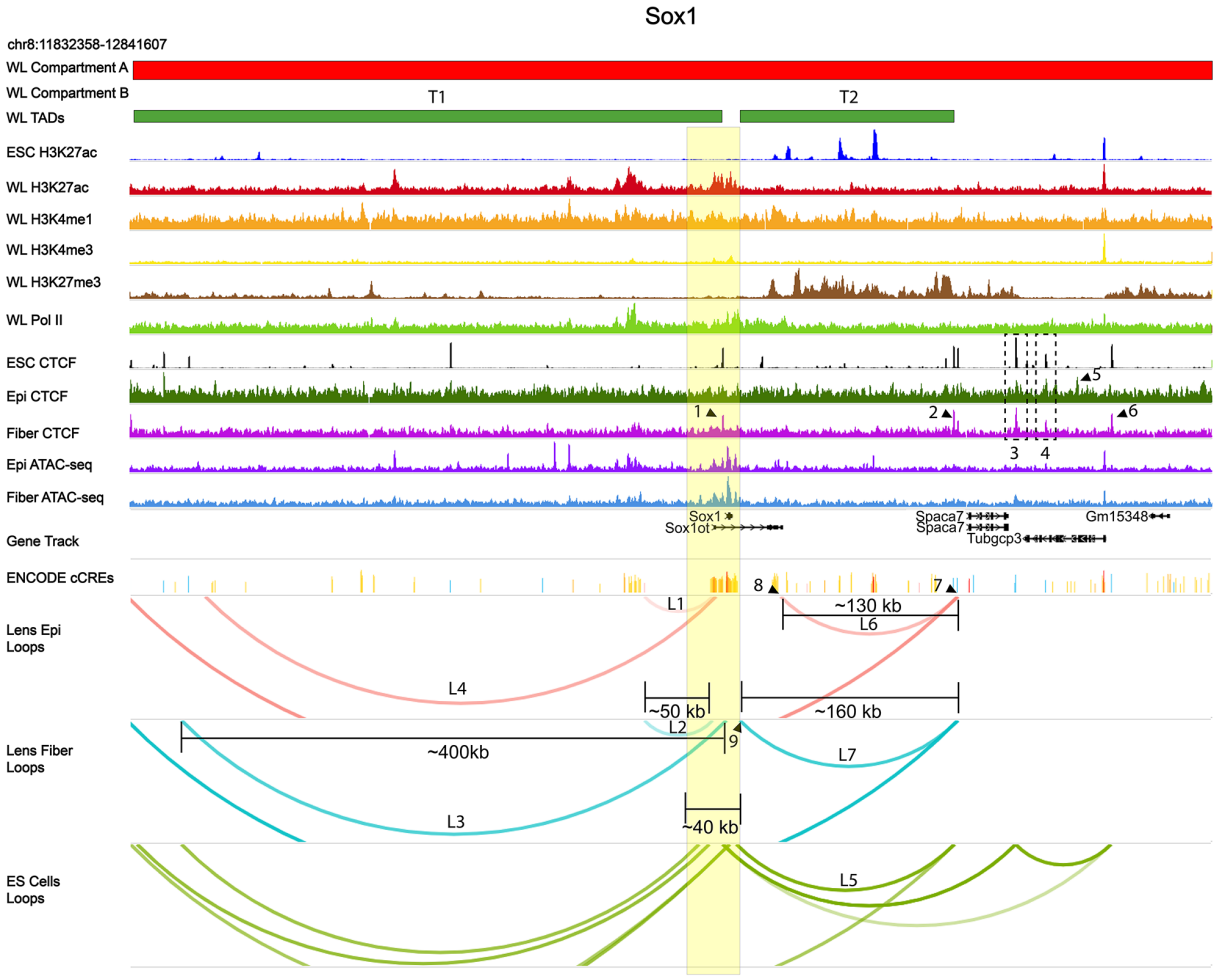
Chromatin loops, CTCF binding and other features of the *Sox1* locus. Sox1 coding regions and majority of overlapping Sox1ot are highlighted in yellow. The Sox1 gene body is located between two large upstream 430 kb and downstream 160 kb TADs (T1 and T2), respectively. See Fig. 10 for individual track description. Specific CTCF peaks and loops discussed in the text are marked by numbered arrows and dotted boxes (1–6) and L1-L7, respectively. See Fig. 10 for individual track description.

**Fig. 12:**
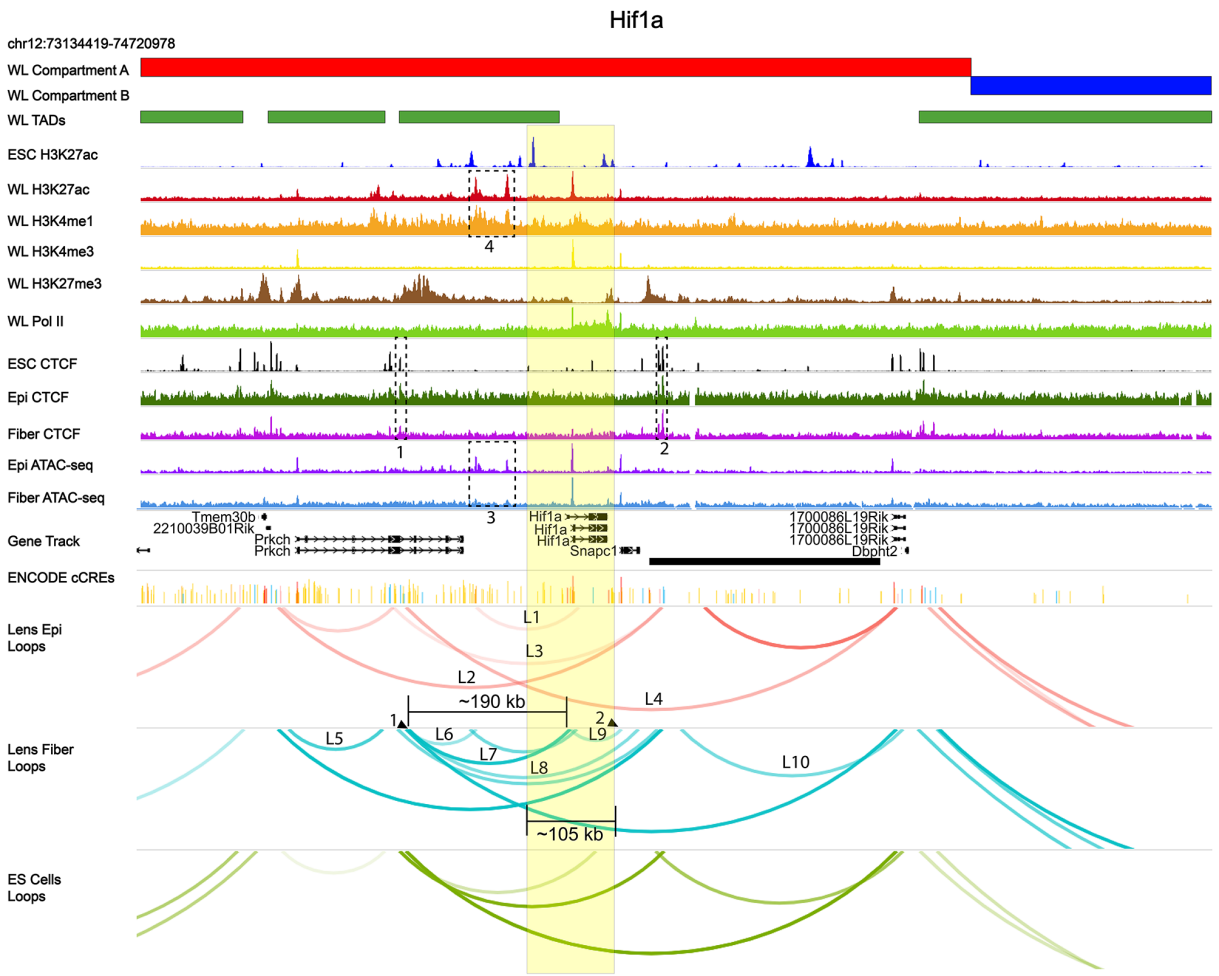
*Hif1a* locus, CTCF binding and its extensive chromatin looping in lens fiber cells. Hif1a locus including its 5’-flanking genomic regions is highlighted in yellow. Lens fiber cell chromatin looping patterns shows marked differences compared to lens epithelium. Two candidate upstream enhancers are boxed in the H3K27ac, H3K4me1 and ATAC-seq tracks. Specific CTCF peaks and loops discussed in the text are marked by numbered arrows and dotted boxes (1–10) and L1-L10, respectively. See Fig. 10 for individual track description

**Fig. 13:**
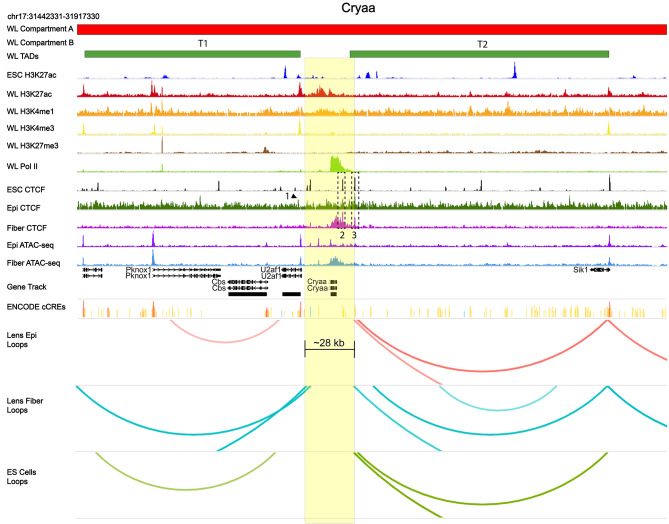
CTCF-binding, RNA polymerase II and other features of the *Cryaa* locus. The *Cryaa* locus including its 5’- and 3’-flanking genomic regions is highlighted in yellow and resides outside of large looping systems. Note two flanking upstream and downstream ~ 140 kb (T1) and ~ 160 kb (T2) TADs, respectively. Specific CTCF peaks discussed in the text are marked by numbered arrows and dotted boxes (1–3). Note an overlap between RNA polymerase II (Pol II) signal across the gene body in lens ChIP-seq and CTCF in fiber cell chromatin. See Fig. 10 for individual track description

**Fig. 14:**
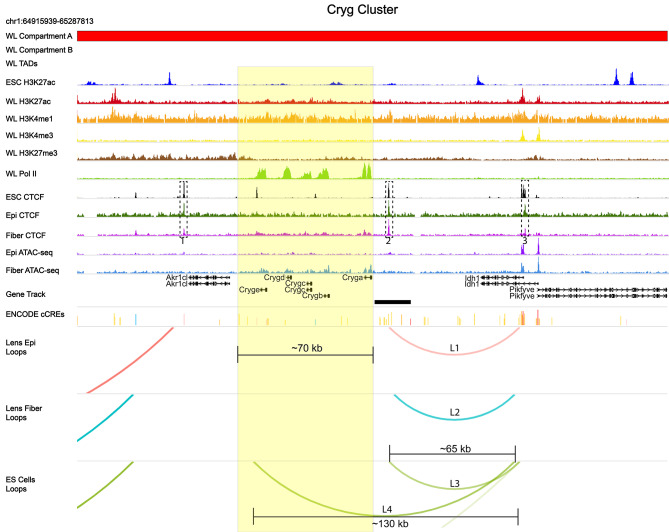
Limited large loop patterns, CTCF binding and other features of the γ-crystallin cluster. The γ-crystallin cluster occupies over 70 kb of DNA (yellow box) and is marked by high RNA polymerase II (Pol II) consecutive domains at the *Cryga*, *Crygb*, *Crygc*, *Crygd*, and *Cryge* loci in lens chromatin. Three CTCF peaks shared by lens epithelium and fibers are marked dotted boxes (1–3). Note increased CTCF broad binding corresponding to RNA polymerase II presence in lens chromatin. No proximal large loops are found through the entire γ-crystallin cluster in both lens chromatins. See Fig. 10 for individual track description

**Fig. 15:**
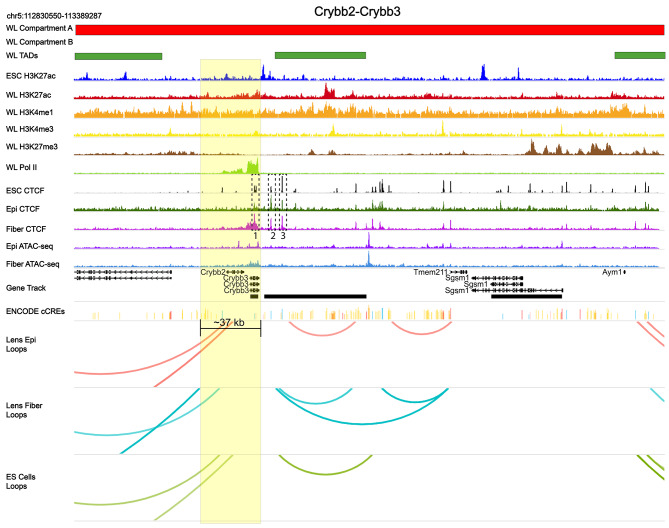
Large loop patterns, CTCF binding, RNA polymerase II, and other features of the *Crybb2*-*Crybb3* locus. Both gene bodies are located between ~ 70 kb upstream and ~ 110 kb long downstream TADs. Similar to the γ-crystallin cluster, no proximal large loop interactions were detected within these loci. Consistent with RNA-seq data [58], the *Crybb3* shows higher Pol II signal compared to the adjacent *Crybb2* locus. Multiple CTCF peaks are found upstream of the *Crybb3* locus (boxes 1–3). A large domain of CTCF overlaps with Pol II at the *Crybb3* gene. See Fig. 10 for individual track description

To fully harness power of the current data, several representative loci are shown together with additional tracks, including CTCF binding (ES cells, epithelium and fibers), our earlier “open” chromatin peaks determined by ATAC-seq [62] and ENCODE *cis*-regulatory elements [103]. In addition, histone PTMs and RNA polymerase II tracks are shown using our other data obtained from newborn lenses [53, 65] as well as H3K27ac data in ES cells [104]. Representative loci encoding major lens regulatory and structural proteins are shown in Figs. 10, 11, 12, 13, 14 and 15 and in Additional File 3. As a result of the initial analyses, a set of three contact maps in ES cells, lens epithelium and lens fibers for individual *Pax6*, *Sox1*, *Hif1a* and Cryaa loci as well as for the *Cryga*-*Crygb*-*Crygc*-*Crygd*-*Cryge* and *Crybb2*-*Crybb3* clustered crystallin loci are shown in Additional File 3: Figure S6-11. Notable 3D-chromatin differences are revealed by the contact maps between the ES and lens cells as shown earlier (see Figs. 2b and 3).

We first show this set of comprehensive tracks at the ~ 1.2 Mb *Pax6* locus (Fig. 10, chromosome 2).

Expression of Pax6 is higher in lens epithelium compared to lens fibers (Additional File 2: Fig. S5). The *Pax6* locus shows a shared complex distal looping network in lens epithelium and fiber cells that is markedly different compared to the ES cells. This loop network spans ~ 670 kb forming nested loop structures sharing multiple loop anchors bound by CTCF. We found three shared CTCF peaks between ES, lens epithelium, and fibers (boxes 1–3). Lens fibers also show three internal CTCF peaks (numbers 4–6) shared with ES cells [66] and peak 4 is shared with rod photoreceptors [58]. Both lens cell types share an upstream distal loop of ~ 50 kb in length bound by CTCF in lens fiber cell chromatin (loops L1-L2). In whole lens chromatin, a 40 kb domain of the *Pax6* locus shows H3K27ac activity (Fig. 10, horizontal bracket) spanning the gene, overlapping with “open” chromatin (see ATAC-seq tracks), and regions rich with candidate *cis*-regulatory elements (cCREs) that is absent in ES cells. Notably, we found two distinct internal loops in the 3’ region (loops L3 and L4) extending across the *Elp4* locus expressed in the opposite orientation only in lens epithelium and another loop extended even further into the *Dnajc24* gene (Fig. 10).

The *Sox1* locus (Fig. 11, chromosome 8) encodes another DNA-binding transcription factor that is more expressed in lens fibers compared to lens epithelium (Additional File 2: Fig. S5) and directly regulates γ-crystallin gene expression [90]. There is also an overlapping Sox1 other transcript (Sox1ot) long noncoding RNA (lnRNA) involved in neuronal differentiation [105]. Sox1 is encoded by a single ~ 1.2 kb exon, marked by H3K27ac domain in lens chromatin, and flanked by strong CTCF binding (Fig. 11, arrow 1, also found in ES cells) in lens fibers proximal to the 5’-promoter (-3.9 kb) region where multiple similar loops (L3-L5) are found in all three chromatin samples (Fig. 11).

Lens epithelium and fiber cells mostly share similar loop anchors but have marked differences between ES cells. One notable difference between ES cells and lens cells is the shared loop anchor near the Sox1ot promoter making a distal contact ~ 50 kb with cCRE enhancer site (loops L1 and L2). In fiber cells, the Sox1 promoter makes a loop formation with an unbound CTCF site ~ 400 kb upstream (loop L3). Five additional CTCF downstream peaks (arrows 1–2, 5–6, dotted boxes 3–4) were found and peak 2 overlaps with loop anchors L5 and L6 in lens fiber and ES cells. A similar sized loop is formed with the Sox1ot promoter near a cCRE region only in lens epithelium (loop L4). Both lens epithelium and fiber share the same CTCF bound distal downstream contact (arrow 7) but have different upstream contacts, one being near the last exon of Sox1ot (cCRE track, arrow 8) in lens epithelium (~ 130 kb), and the other within an intronic element of Sox1ot (~ 160 kb) in lens fiber (cCRE track, arrow 9). Overall, the present data show that the *Sox1* gene body is located between two large upstream and downstream TADs of 430 kb (T1) and 160 kb (T2) in size, respectively (Fig. 11).

It has been shown earlier that lens fiber cell differentiation occurs at hypoxia conditions [106]. The basic helix-loop-helix transcription factor Hif1α is the main regulator of hypoxia-regulated transcription [107, 108]. Depletion of Hif1α in mouse lens disrupts its growth and lead to lens degeneration [109]. *Hif1a* locus (Fig. 12, chromosome 12) shows distinct chromatin looping signatures between lens epithelium and lens fiber cells. Four loops found in lens epithelium are shared by lens fibers (loops L1-L4); however, lens fibers display six additional unique chromatin loops, L5-L10. Two notable lens fiber cell loops (loops L6 and L7) make contacts with *Hif1a* promoter and ~ 190 kb upstream intronic element (fiber cell loops, arrow 1) of the *Prkch* gene marked by CTCF binding (dotted box 1) and a unique downstream looping contact with the Snapc1 promoter region (fiber cell loop L9, arrow 2). Both CTCF (dotted boxes 1 and 2) and ATAC-seq data (dotted box 3) do in lens epithelium and fiber show similar peak profiles across the locus. Whole lens H3K4me1 and H3K27ac data (dotted box 4, distinct from ES cells) predict two potential distal 5’-enhancers of the *Hif1a* locus (loop L1) and overlap with open chromatin regions (dotted box 3), Most loops found here originate from CTCF binding in lens epithelium, fibers and ES cells (Fig. 12, dotted boxes 1 and 4). Thus, loops L6-L9 in lens fibers appear as potential regulatory mechanisms of Hif1a expression in hypoxic (1.5-2% O_2_) lens fiber cells [110].

The most highly expressed gene in the lens encodes the αA-crystallin (Cryaa, chromosome 17) and is directly regulated by Pax6, c-Maf, CREB, c-Jun and Etv5 transcription factors [111–113] and marked by abundant RNA polymerase II across over 12 kb of its coding region (Fig. 13). Two 3’-regions of CTCF-binding (dotted boxes 2 and 3) were found in all three chromatins while “proximal” CTCF-binding was only detected at the 5’-region of the *Cryaa* locus in lens epithelium (arrow 1). A striking difference between lens epithelium and fiber cells was increased presence of CTCF found in lens fiber cells across the entire *Cryaa* gene body and overlapping with RNA polymerase II. The internal “peak” of this CTCF domain corresponds to strong binding of CTCF (box 2) in ES cell chromatin.

A cluster of five γ-crystallin genes (*Cryga*, *Crygb*, *Crygc*, *Crygd* and *Cryge)* occupies over 70 kb of chromosome 1 in the absence of any major loops in both lens chromatins analyzed and is marked by CTCF binding at both flanking sides (Fig. 14, dotted boxes 1–2). Both lens cells and ES cells share distal loop structures (loops L1-L3) of ~ 60–65 kb in length outside of this cluster of five crystallin genes. Both anchors of this loop structure are bound by CTCF (dotted box 3). In contrast, in ES cells, not expressing crystallin genes, a large distal ~ 130 kb loop (L4) spans the entire γ-crystallin cluster.

Inspection of chromatin organization of the *Crybb2*-*Crybb3* locus (Fig. 15, chromosome 5) also shows large loops originating just downstream of the 3’-UTR of the *Crybb2* gene that is expressed at much lower level compared to the adjacent *Crybb3* in newborn lens [64] (Additional File 2: Fig. S5).

Three CTCF peaks are found upstream of the Crybb3 promoter in both lens chromatins as well as in ES cells (Fig. 15, dotted boxes 1–3) where other multiple outside loops originate. Most importantly, a marked overlap between RNA polymerase II and CTCF binding across the entire *Crybb3* locus and reduced amounts at the *Crybb2* locus are found (Fig. 15). For contact maps of each cell type, see Additional File 3: Figs. S6-S11.

Three additional groups of individual and/or clustered loci include genes encoding DNA-binding transcription factors regulating lens development (Foxe3, Gata3, Hsf4, Maf, Prox1, Pitx3, Rarb, Sox2 and Tfap2a), proteins involved in lens morphogenesis and differentiation (Bfsp2, Bmp4, Bmp7, Cryba4-Crybb1, Cryba1, Cryba2 and Rb1), and novel cataract genes identified by recent human genome-wide association studies (GWAS), such as *Casz1*, *Gstm2*, *Krtp2-Dpm3-Efna1* and *Sema4d* [80] as shown in Additional file 4, Figs. S12-20; file 5, Figs. S21-27; and file 6, Figs. S28-31; respectively. For example, the centrally located *Gstm2* [80] is a part of larger cluster of seven *Gstm* genes and within a single loop found only in lens fiber cells. The *Rb1* locus encodes the retinoblastoma protein (pRb) highly expressed in lens fibers and both controlling their cell cycle exit-coupled terminal differentiation [114] and binding to Pax6 proteins [115, 116]. It shows a unique fiber-cell specific long-range loop marked by CTCF binding sites in all three chromatins. The *Sema4d* locus [80] encoding plasma membrane receptor protein semaphorin 4D is also located within a region lacking large loops described above. Taken together, detailed analyses of chromatin looping show unique organization of multiple crystallin loci as well as other loci encoding important lens regulatory and structural proteins. The data suggest an intriguing possibility that clustered CTCF proteins participate in RNA polymerase II convoys and/or formation of the condensates supported by our earlier finding of colocalization of nascent RNA transcription detected using single molecule RNA FISH and immunofluorescent visualization of transcriptionally active RNA polymerase II [51].

## Discussion

The present Hi-C data show for the first-time 3D-chromatin organization in mouse newborn lens chromatin separated into lens epithelial and lens fiber cells and direct comparisons with chromatin organization of ES cells. In addition, these data are analyzed in the context of CTCF binding determined by ChIP-seq as well as our earlier studies of chromatin landscape by ATAC-seq and ChIP-seq studies of histone PTMs and RNA polymerase II. These lens data can serve for comparative purposes with other mouse tissues and provide potential insights into distal non-coding variants associated with abnormal lens development, cataracts, and other diseases involving ocular lenses.

Hi-C experiments have already been conducted using multiple mouse and human cells and tissues of different complexity. Multiple similarities exist between erythroid maturation and differentiating lens fiber cells. Mammalian erythrocytes are marked by high levels of α- and β-globin gene expression that are directly comparable to crystallin gene expression at the quantitative levels (comparing bulk RNA-seq data) with lens fiber cells [53]. In addition, chromatin condensation and transfer of nuclear proteins into the cytoplasm occurs in maturing red blood cells followed by nuclear extrusion [117, 118]. In contrast, lens fiber cell nuclei disintegrate within the individual lens fiber cells as described above (see Fig. 9). Our data using RNA FISH to detect nascent RNA expression show that *Cryba4* and *Crybb1* genes can be simultaneously transcribed from adjacent alleles [119]. It has been shown earlier that low insulation scores have been associated with upregulation of genes related to terminal differentiation [10, 66].

The unexpected presence of CTCF and overlap with RNA polymerase II signals in *Cryaa* and *Crybb3*-*Crybb2*, *Cryba4*-*Crybb1* and *Cryga*-*Cryge* gene clusters represents potentially a new research avenue to understand composition of condensates formed at late stages of lens fiber cell differentiation, just prior their denucleation, originally visualized through co-localization studies of nascent crystallin mRNA transcription and transcriptionally active RNA polymerase II [51]. To assess the association of RNA Polymerase II and CTCF signals using an unbiased approach, we analyzed genome-wide both ChIP-seq signals. We specifically evaluated the mean Pol II and CTCF signals across all gene bodies, focusing on protein-coding genes, and comparing them with gene expression data (see Materials and Methods for details). Our genome-wide analysis shows that the crystallin family of genes indeed both had the highest RNA Polymerase II-CTCF signal overlap and expression levels (Additional File 12: Fig. S32a). Outside of the crystallin genes, we found other highly expressed lens genes with high RNA Polymerase II-CTCF signal overlaps. Some of these genes include Mip/Aqaporin 0, Gja8, and Vim, all genes are involved in lens fiber cell differentiation and implicated in cataract formation (Additional File 12: Fig. S32b). The most highly expressed αA-crystallin locus [53, 64] show highly specific CTCF binding in lens fiber cells that overlaps with transcriptionally active RNA polymerase II detected in whole lens chromatin (Fig. 13). Similar patterns are also found at both *Crybb2*-*Crybb3* and *Cryba4*-*Crybb1* clusters and individual *Cryba1 and Cryba2* regions (Additional File 5 Figs. S24 and S25). This trend is even detectable at the γ-crystallin gene cluster (Fig. 14). Recent studies have shown presence of RNA polymerase II and CTCF via their intrinsically disordered domains in formation of phase-separated droplets [120]. It is thus possible that the presence of CTCF in these highly transcribed regions is indirect and related to the protein-protein interactions within the phase separated structures [121]. Our earlier studies of nascent expression of *Cryaa*, *Cryba4*, *Crybb1*, *Crybb3* and *Cryga* genes evaluated by single molecule RNA FISH showed correlation with the largest foci containing transcriptionally active RNA polymerases II [51, 119]. Thus, these findings may relate to a broad multifunctionality of CTCF outside of organizing TADs and forming clusters of 2–8 CTCF molecules [32] that may even at much larger quantities assist large RNA polymerase II convoys transcribing crystallin loci as found in lens chromatin [46, 53]. Another possibility is that the CTCF proteins, through their RNA binding zinc-finger domains [22], can also bind nascent crystallin mRNAs. This possibility is not mutually exclusive with the other mechanisms described above. Finally, recent studies using cancer cell line have shown presence of CTCF in phase-separated condensates and their integrity requires presence of CTCF suggesting instructive function of these proteins for condensate formation [122]. Thus, future studies of these phenomena in lens fiber cell chromatin are highly warranted.

In addition to the representative loci discussed above (Figs. 10, 11, 12, 13, 14 and 15), three groups of loci are included for comparative purposes. For example, Sox2 (Additional file 4, Fig. S19) regulates pluripotency of ES cells [123] as well as early stages of lens placode morphogenesis [124, 125]. The large *Sox2*-Sox2ot locus includes multiple proximal and distal enhancers our recent studies demonstrated different DNA methylation patterns between ES and both types of lens cells [63]. FoxE3 (Additional File 4, Fig. S13) is an another DNA-binding transcription factor regulating early lens morphogenesis [126]. The second group includes *Cryba4-Crybb1*, *Cryba1* and *Cryba2* loci (Additional File 5, Figs. S24-26), *Bfsp2*, encoding lens-specific intermediate beaded filament protein [127], also shows co-localization of RNA polymerase II with CTCF region in lens fibers (Additional File 5, Fig. 21). Finally, four novel cataract loci, including *Casz1*, *Gstm2*, *Krtp2-Dpm3-Efna1* and *Sema4d*, identified by recent human GWAS studies [80], are also shown in Additional file 6, Figs. S28-31, that might help in the interpretation of non-coding variants if found in evolutionarily conserved distal regions marked by looping anchors. Indeed, GWAS found that most variants in a wide spectrum of human diseases are located outside of protein-coding regions [128]. Identification of truly causal GWAS variants is challenging given their repertoire and distal enhancers representing the most challenging tasks [128]. Thus, the present Hi-C studies will not only aid in interpretation of GWAS studies of cataract genes [80] and of the WAGR syndrome [74, 75] but also aid studies of the microphthalmia-anophthalmia-coloboma (MAC) syndrome caused by mutations in genes with multiple tissue-specific distal enhancers such as *PAX6*, *SOX2*, *ATOH7*, *OTX2*, *VSX2*, *FOXE3*, *BMP4*, *MAB21L1* and other loci [129–131] due to high evolutionary conservation of transcriptional control of these genes between human and mouse. For example, Hi-C maps of promoter-enhancer interactions in neural tissues [132], multiple sclerosis [133] and age-related macular degeneration [59] have already been used to analyze the GWAS data.

Multiple roles of CTCF in DNA- and RNA-binding can be also inferred from different subnuclear localization related to lens differentiation. We found marked difference of CTCF nuclear localization in lens epithelium and fiber cells that is both temporally and spatially regulated (Fig. 9). Outside of being an insulator protein regulating genome organization, previous studies have shown CTCF to be involved in epigenetic control of rDNA and enhancement of rRNA transcription catalyzed by RNA polymerase I within the nucleoli [134, 135]. Other studies also show CTCF regulates myeloid and erythroid differentiation in human cell lines [136, 137]. Taken together, high abundance of CTCF within nucleoli in differentiating lens epithelium and fiber cells may be a cellular mechanism to augment ribosomal biogenesis to meet high demands for translational output of crystallin proteins in maturing lens fiber cells [40].

Lens chromatin landscape is regulated by various chromatin remodeling complexes as show by lens-specific depletions of Brg1 (Smarca4) [138], Snf2h (Smarca5) [49], CBP and p300 [139], Ncoa6 [140] and Znhit1 [141] proteins in mouse models. We have shown localization of Brg1, Snf2h, p300 and CBP at the 16 kb Cryaa locus using qChIPs in mouse lens chromatin [112, 142] and that Pax6 forms complexes with BAF complexes in neurons [143], retina [144] and lens [112, 145], Recent studies have shown co-localization of Snf2h/Smarca5 with CTCF in human cell lines [146]. Thus, Snf2h proteins found at the *Cryaa* locus [112] might be also involved in our findings of CTCF across the *Cryaa* locus in lens fiber cell chromatin. This another mechanism is not mutually exclusive with those described above.

Future studies will be aimed to pursue parallel opportunities to employ living cells to study cohesin and condensin molecular machines governing ATP-dependent loop extrusion [28], use of single cells combined with Hi-C [147], detailed analysis of the *Pax6* locus using 4 C-seq [148], mapping of enhancer RNAs via PRO-seq [149], deletion of candidate enhancers in the *Pax6*, *Prox1*, and *Hif1a* loci together with transgenic reporter assays, and conditional inactivation of CTCF [150] using lens-specific Cre lines acting in more advanced stages of lens differentiation [151]. Interesting loci can be selected from the present studies and subjected to both 3 C studies to map looping patterns with much improved resolution and directly visualize these interactions using DNA FISH. Finally, chromatin structural modeling (HiP-HoP) based on a combinatorial use of ATAC-seq, H3K27ac, and CTCF data [13] just requires mapping of the H3K27ac landscape in microdissected P0.5 lenses.

In conclusion, the present study has expanded earlier transcriptomics and epigenomics data on mouse lens embryonic development and differentiation that now includes chromatin loops and paves the road for similar studies using human lens cells. Future studies will probe chromatin condensation within lens fiber cells undergoing early stages of their denucleation while preserving the maximal transcriptional output of β- and γ-crystallin genes just prior their abrupt disintegration. Super-resolution microscopy now allows quantification of transcriptionally active RNA polymerase II, CTCF and other proteins in parallel with direct visualization of nascent crystallin gene expression detected by single molecule RNA FISH within individual nuclei of differentiating lens fiber cells [51, 119].

## Materials and methods

### ES cells and lens tissues

To study chromatin interactions of differentiating lens cells, newborn (P0.5) CD-1 mice and mouse ES cells were used. Two biological replicates of lens epithelium, lens fiber and ES cells were used for statistical power. Lenses were dissected at P0.5 and then micro-dissected into lens epithelium and lens fiber under a dissection microscope. Each replicate of lens tissue was comprised of 30 lens epithelium and fiber samples. Samples were kept on dry ice for the duration of the dissection process. Samples were then homogenized using a disposable pestle tissue grinder. Homogenized tissues were fixed in 2.0% formaldehyde for 10 min at room temperature. Formaldehyde was quenched with 0.125 M glycine solution. Mouse ES cells v6.5 (mixed 129/B6, male) were provided by Dr. Meelad Dawlaty [13]. Cells were grown under feeder-free conditions on 0.2% gelatin and supplemented with LIF (24 ng/mL). Each replicate contained ~ 2.0 × 10^6^ cells and were harvested near ~ 80% confluency. The crosslinking of ES cells was performed as described above.

### Generation of Hi-C library and sequencing

The Hi-C library was generated using the Arima-HiC kit according to the manufacturers protocols (A510008) and performed by the NYU Langone Health Genome Technology Center (New York, NY). DNA libraries were sequenced on the Illumina NovaSeq 6000 with ~ 700 × 10^6^ reads per sample with mean quality score Q > 36.

### Quantitative Hi-C analyses and statistics

Read alignment and computation of Hi-C contact maps were performed using the ENCODE Hi-C pipeline (code available at https://github.com/ENCODE-DCC/hic-pipeline). Alignment was performed within the pipeline using bwa-mem (Li 2013 https://arxiv.org/abs/1303.3997). Contact map computation was performed with maximum resolution = 5 kb within the pipeline using Juicer [77, 152]; contact maps created from filtered reads aggregated across two biological replicates of two lanes each per cell type with alignment quality score MAPQ > = 30 were used for subsequent per-cell type analyses. For whole-lens contact maps, reads from all replicates from both epithelium and fiber were pooled. Loop and TAD calling were done with Juicer Tools using HiCCUPS and Arrowhead, respectively [77]. Default parameters were used for all pipeline analysis stages. Whole-lens contact maps and subsequent analyses were created from combined lens epithelium and fiber contact maps.

For A/B compartment analysis, dcHiC [68] was used to calculate the first two principal components (PCs) of contact maps at 10 kb resolution, select the appropriate PC for downstream analysis, normalize, sign-correct, and comparatively analyze the selected PC between cell types. Association of genes with compartmentalization was performed as follows. Transcriptional start sites were obtained from the RefSeq database [153]. All transcriptional isoforms were kept for each gene ID. Promoter regions were defined as 2 kb upstream/500 bp downstream of transcriptional start sites. Intersections between promoter regions and A/B compartment score bins were obtained using bedtools intersect. For promoters that intersected with more than one compartment score bin, the bin with the largest overlap was kept, and its compartment label used to annotate the gene associated with the promoter. Visualizations of contact maps were created using WaSHU Epigenome Browser and Juicebox [152, 154].

### CTCF ChIP-seq and motif analysis

P0.5 lenses (*n* = 200) were obtained from CD-1 mice, micro-dissected into lens epithelium and lens fibers and stored in liquid nitrogen prior the use as we described earlier [53]. Preparation for ChIP-seq was provided by ActiveMotif (Carlsbad, CA, U.S.A.). Briefly, immunoprecipitation was performed on 12 µg chromatin from microdissected lens cells with 5 µl anti-CTCF antibody (ActiveMotif cat. # 61,311, lot #11,219,006), *n* = 2 biological replicates. The 75-nt single-end (SE75) sequence reads generated by Illumina sequencing (using NextSeq 500) were mapped to the genome using the BWA algorithm (“bwa aln/samse” with default settings) [155]. Uniquely mapped reads passing Illumina’s purity filter with < = 2 mismatches were retained for downstream analyses. Duplicate reads were removed. Peaks were called using MACS2 [156]. Methylation profiles within CTCF peaks were plotted using deeptools 3.5.1 [157].

### Analysis of RNA expression in lens cells

Bulk mouse lens RNA-seq data were generated earlier [64]. Boxplot showing top differentially expressed genes with fragments per kilobase of transcript per million mapped reads (FPKM). FPKM values were Log2 transformed for scaling purposes. Callout boxes were used to highlight the most upregulated genes.

### Cross-analysis of ChIP-seq and mRNA expression

Genes were ranked by their expression levels according to the earlier RNA-seq data [64]. The top 100 most expressed genes in fiber cells were selected. The longest transcripts associated with each gene ID were obtained from the RefSeq All database. Mean epithelium-specific and fiber-specific CTCF ChIP-seq signals and whole-lens RNA Polymerase II ChIP-seq signals were calculated across each transcript using multiBigwigSummary from the deeptools package. The data were merged with expression data of the top 100 expressed genes. Non-coding genes were manually removed to obtain the final list (*n* = 63) associating expression, CTCF, and RNA Polymerase II signals in top-expressed genes in lens fiber.

### Immunofluorescence analysis of lens nuclei

Eyes were fixed in 4.0% paraformaldehyde for 2 h at room temperature, transferred into 30% sucrose for cryopreservation and embedded in OCT (Tissue Tek). Tissue was stored at -80^o^ C until used. Cryostat sections were cut in the transverse plane at 10 microns and stored at -20^o^ C until IF protocol. Slides were permeabilized with 0.5% Triton-X-100 in PBS (PBS-T) for 30 min at room temperature. Slides were washed 3x PBS for 5 min each. Following permeabilization, slides were blocked with 4.0% BSA for 1 h at room temperature. Slides were washed 3x PBS for 5 min each. Slides were incubated for 24 h at 4 C with primary antibodies diluted in 1.0% BSA and PBS-T in a humidity chamber. Slides were then washed 4x PBS for 10 min each. Slides were incubated with secondary antibodies diluted in 1.0% BSA in PBS-T for 2 h at room temperature in humidity chamber at room temperature then washed 3x PBS for 10 min each, then a final wash in PBS with Hoechst (1:2,000) for 10 min. Slides were imaged on Leica SP8 at 63x magnification. Antibodies and dyes: CTCF (Santa Cruz sc-271,514, 1:100), Alexa Flour 488 (Jackson 115-547-185, 1:250) and Hoechst 33,342 (Fisher H3570, 1:2,000).

### Electronic supplementary material

Below is the link to the electronic supplementary material.

Supplementary Material 1

Supplementary Material 2

Supplementary Material 3

Supplementary Material 4

Supplementary Material 5

Supplementary Material 6

Supplementary Material 7

Supplementary Material 8

Supplementary Material 9

Supplementary Material 10

Supplementary Material 11

Supplementary Material 12

## Data Availability

Hi-C and CTCF ChIP-seq data were deposited into Gene Expression Omnibus (GEO) with accession ID GSE243851. ATAC-seq (GSE124497; [62]), RNA-seq (GSE113887; [64]), WGBS (GSE213901; [63]), ChIP-seq (GSE66961; [53, 65]).

## Data availability

No datasets were generated or analysed during the current study.

## Abbreviations

ATAC-seq: Assay for transposase-accessible chromatin with high-throughput sequencing
ChIP: Chromatin immunoprecipitation
ES: Embryonic stem
cCREs: ENCODE candidate *cis*-regulatory elements
FISH: Fluorescence in situ hybridization
GO: Gene ontology
GWAS: Genome-wide association studies
kb: Kilo base pairs
IDRs: Intrinsically disordered regions
LCR: Locus control region
OFZ: Organelle free zone
PTM: Posttranslational modification
PCA: Principal component analysis
SMCs: Structural maintenance complexes
TADs: Topologically associated domains
3D: 3-dimensional
WGBS: Whole genome bisulfite sequencing
