# Supplementary material for: Analysis of long-range chromatin contacts, compartments and looping between mouse embryonic stem cells, lens epithelium and lens fibers
Source: Epigenetics Chromatin. 2024 Apr 20;17:10. doi: 10.1186/s13072-024-00533-x (PMC11031936; doi:10.1186/s13072-024-00533-x)
Supplement: Supplementary file 2 — Supplementary Material 2 [file 13072_2024_533_MOESM2_ESM.docx]

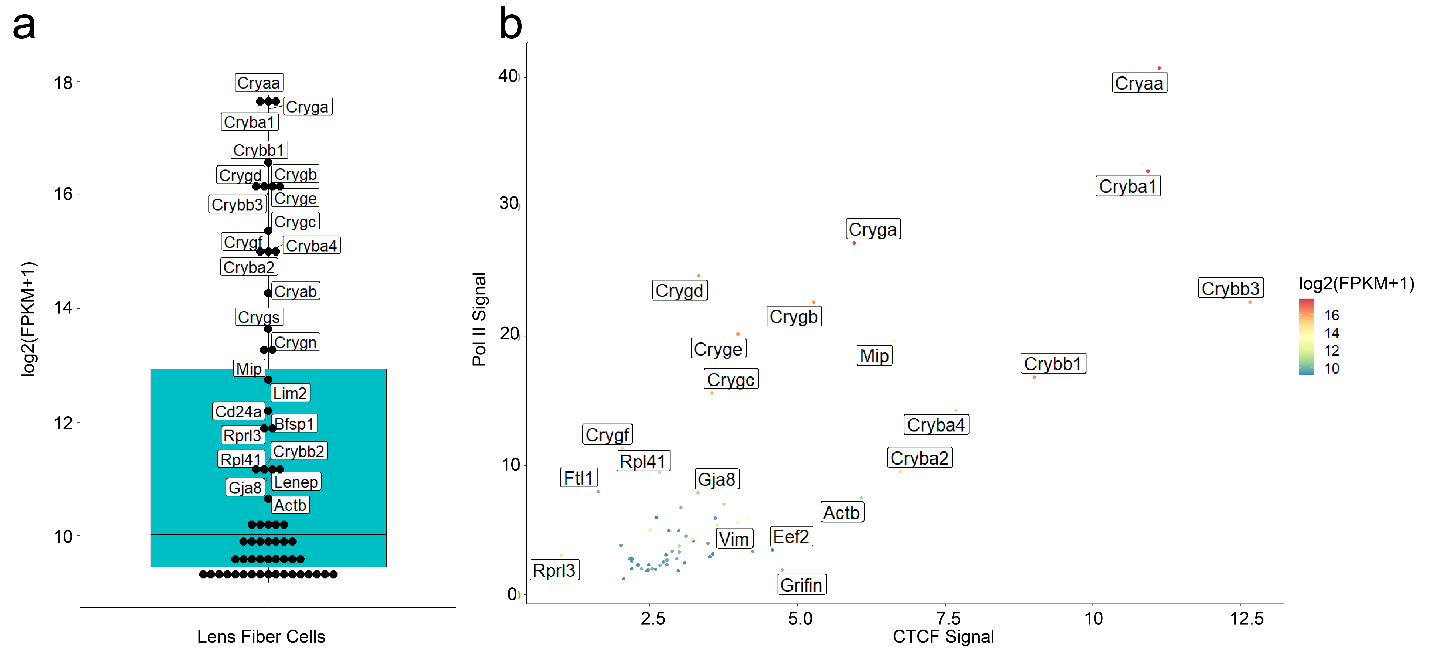


**Fig. S32: Genome-wide CTCF and RNA Polymerase II overlap analysis at gene bodies encoding highly expressed protein coding mRNAs.**

a) Expression levels of protein coding genes in lens fiber cells with the highest CTCF and RNA Polymerase II signals shows an abundance of mRNA encoding individual crystallins, lens fiber-specific intermediate filament proteins, gap junction proteins, and ribosomal proteins. b) Scatter plot of genes shown in panel a) with CTCF (x) and Pol II (y) signal. Gene expression level is color coded with log scale in the legend.
