# Supplementary material for: Analysis of long-range chromatin contacts, compartments and looping between mouse embryonic stem cells, lens epithelium and lens fibers
Source: Epigenetics Chromatin. 2024 Apr 20;17:10. doi: 10.1186/s13072-024-00533-x (PMC11031936; doi:10.1186/s13072-024-00533-x)
Supplement: Supplementary file 4 — Supplementary Material 4 [file 13072_2024_533_MOESM4_ESM.docx]

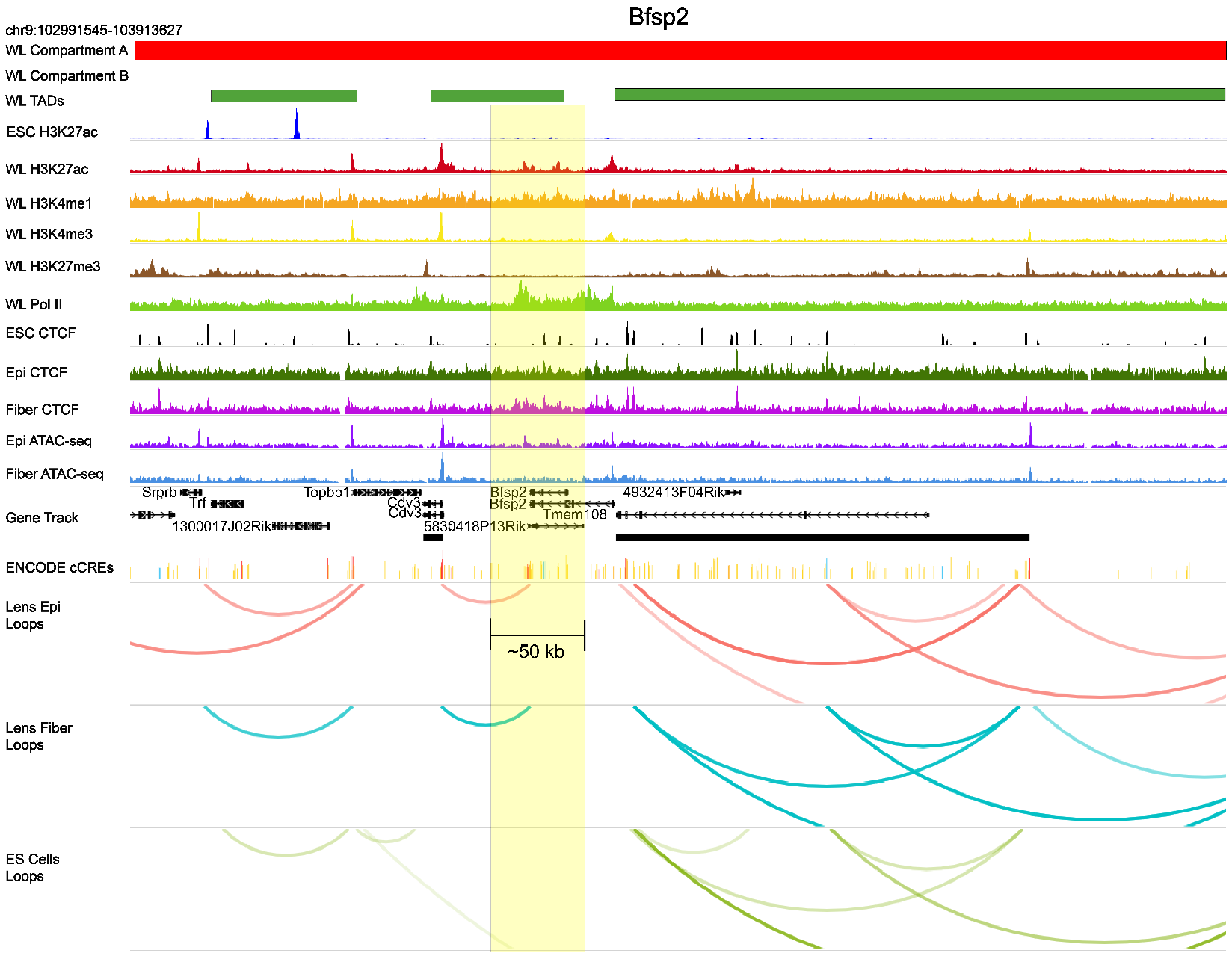


**Fig. S21: Chromatin loops, CTCF binding and other features of the Bfsp2 locus.**

Bfsp2 (yellow box) encodes an abundant lens-specific intermediate filament protein. Relatively short-range loops in epithelium and fibers were found close to the 3’-UTR region. The Bfsp2 locus shows a broad region of RNA Polymerase II signal across the gene body and increase of CTCF presence in lens fiber chromatin. A large TAD (~520 kb) located upstream of Bfsp2 transcriptional start site contains multiple shared CTCF-bound loop structures between lens epithelium and fiber cells. See Fig. 10 for individual track description.


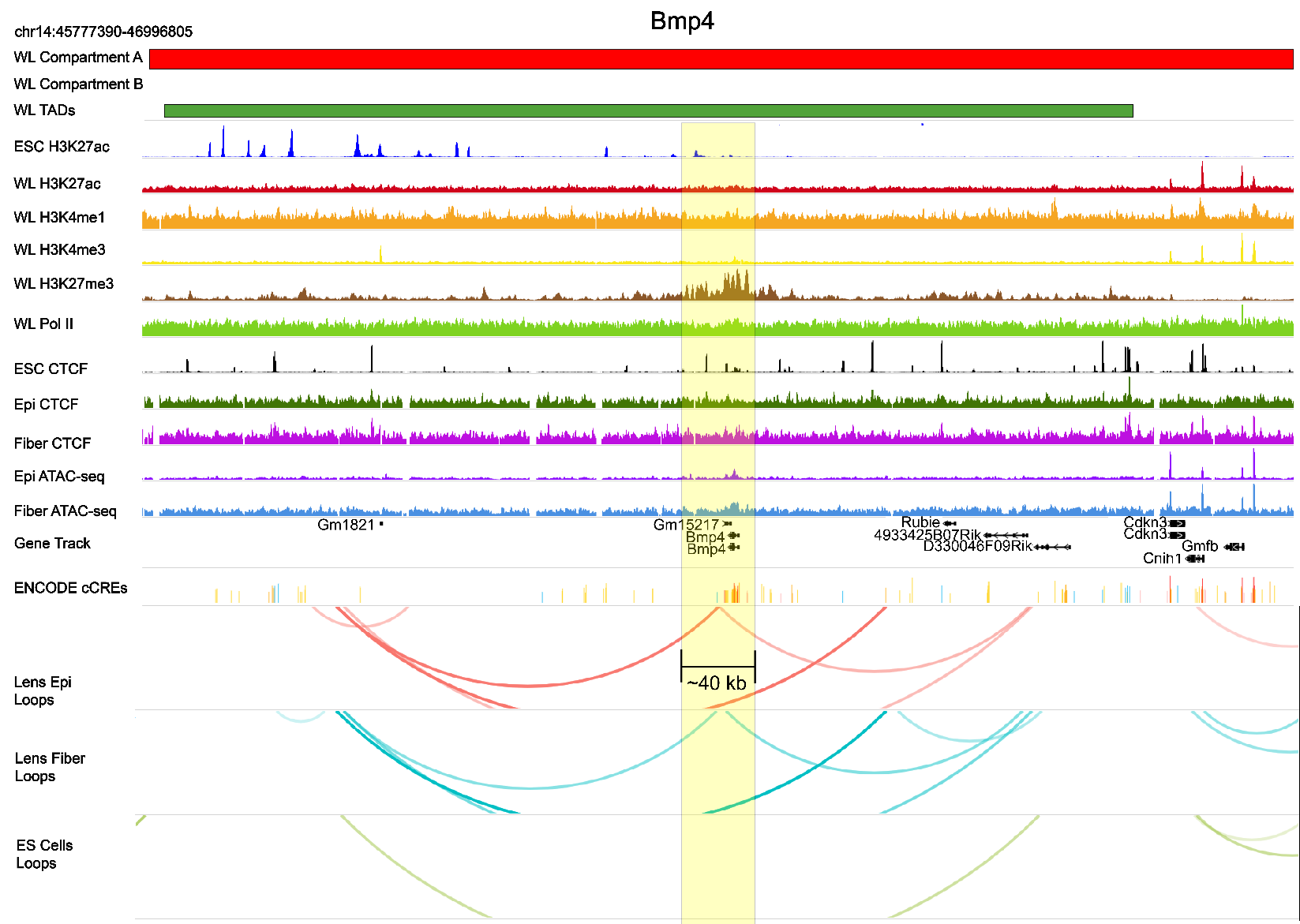
 **Fig. S22: Chromatin loops, CTCF binding and other features of the Bmp4 locus.**

Bmp4 secreted proteins are required for both lens induction and proper optic cup formation. The Bmp4 locus (yellow box) shows a complex loop network that spans both upstream and downstream of the locus in both lens epithelium and fiber cells. See Fig. 10 for individual track description.


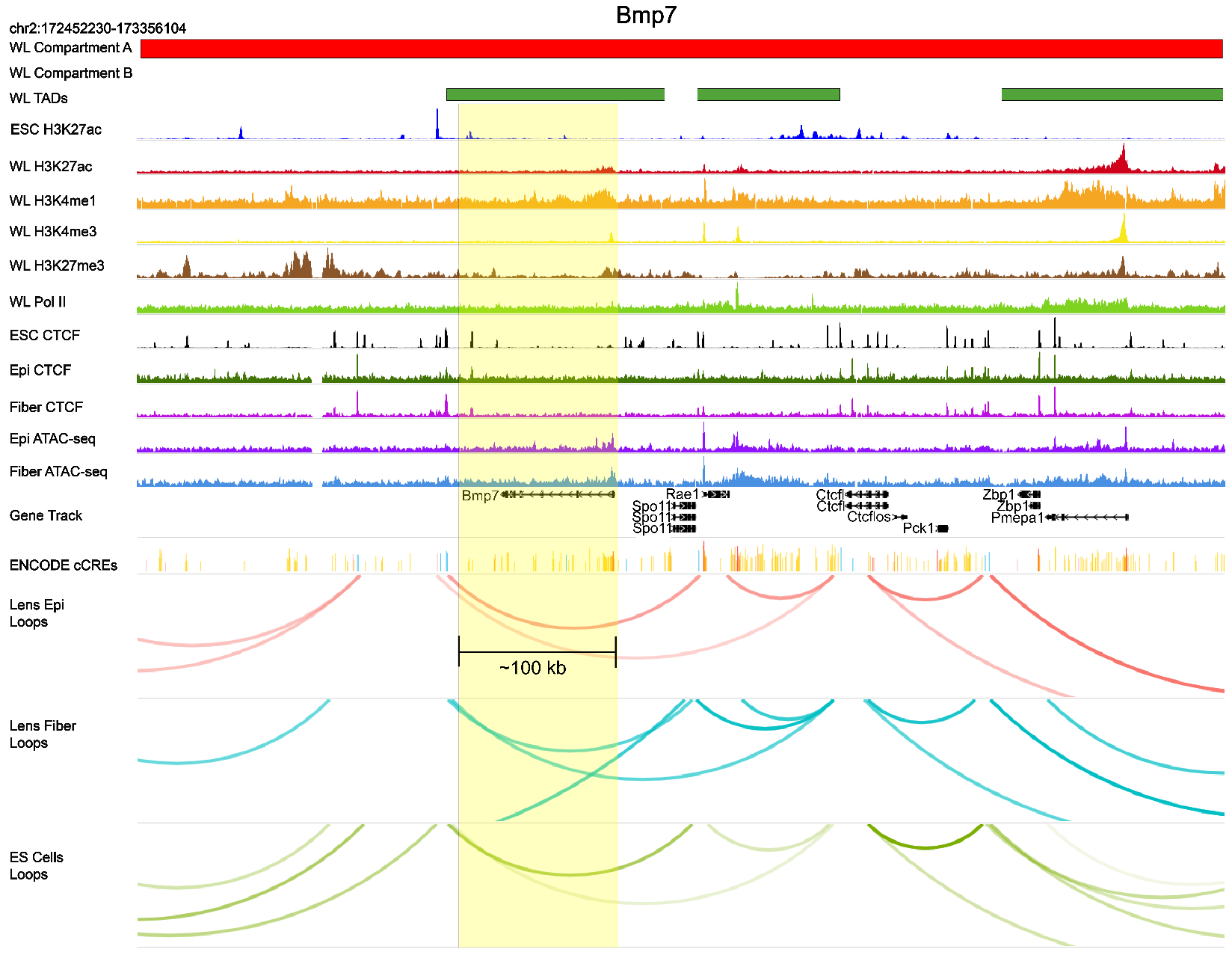


**Fig. S23: Chromatin loops, CTCF binding and other features of the Bmp7 locus.**

Like Bmp4, Bmp7 proteins also regulate lens development. A single TAD of ~150 kb was detected that encompasses Bmp7 locus (yellow box). Both H3K27ac and H3K4me1 domains were detected at the Bmp7 promoter. Chromatin loop structures are mostly shared between lens epithelium and fiber cells, forming contacts near to multiple shared CTCF sites. See Fig. 10 for individual track description.


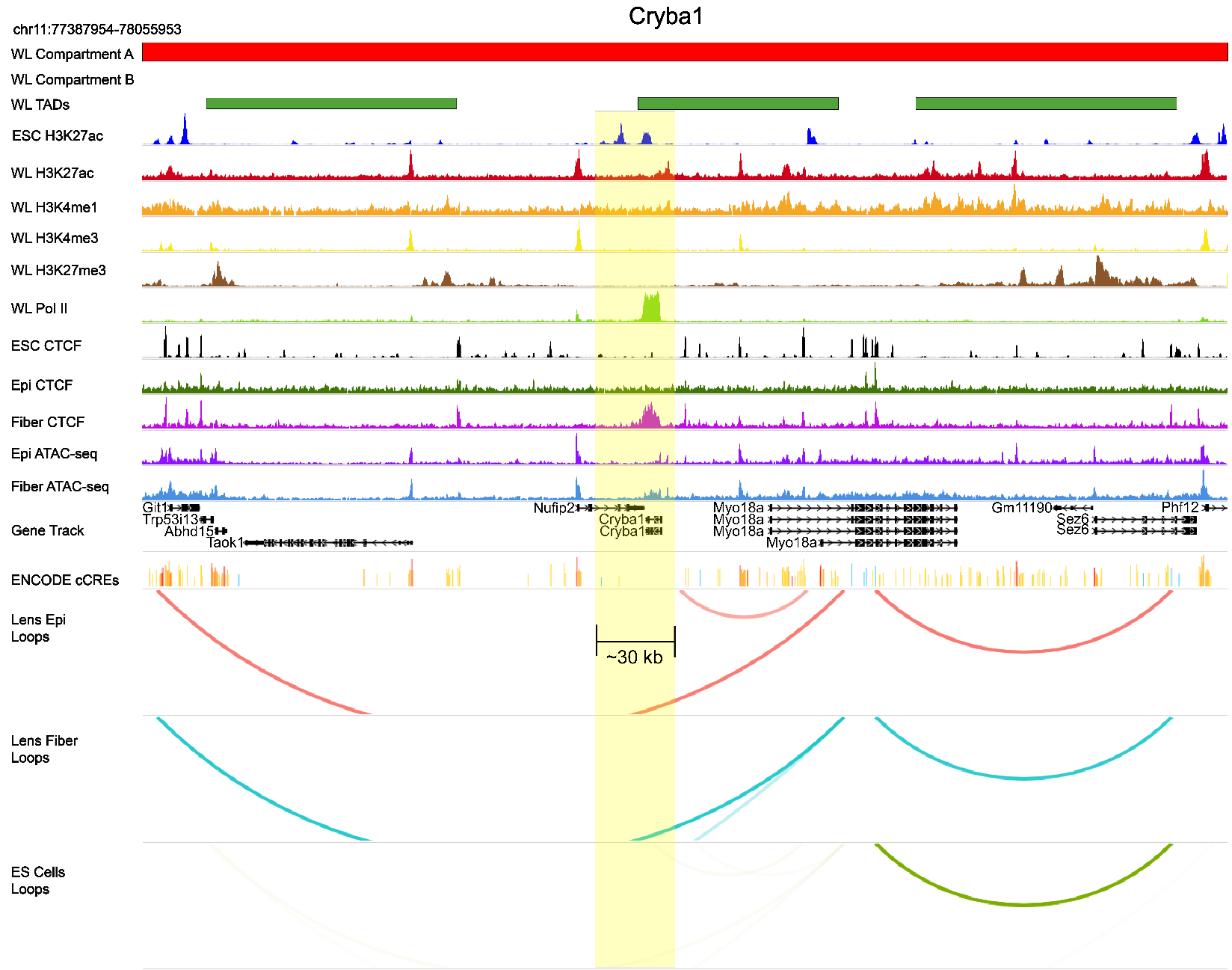


**Fig. S24: Chromatin loops, CTCF binding and other features of the Cryba1 locus.**

The Cryba1 locus (yellow box) is located on the edge of a TAD boundary. Lens epithelium shows a long-range interaction ~10 kb upstream with distal loop of ~60 kb. Note an overlap between Pol II and CTCF bound region in lens fiber cell chromatin found in other crystallin loci. See Fig. 10 for individual track description.


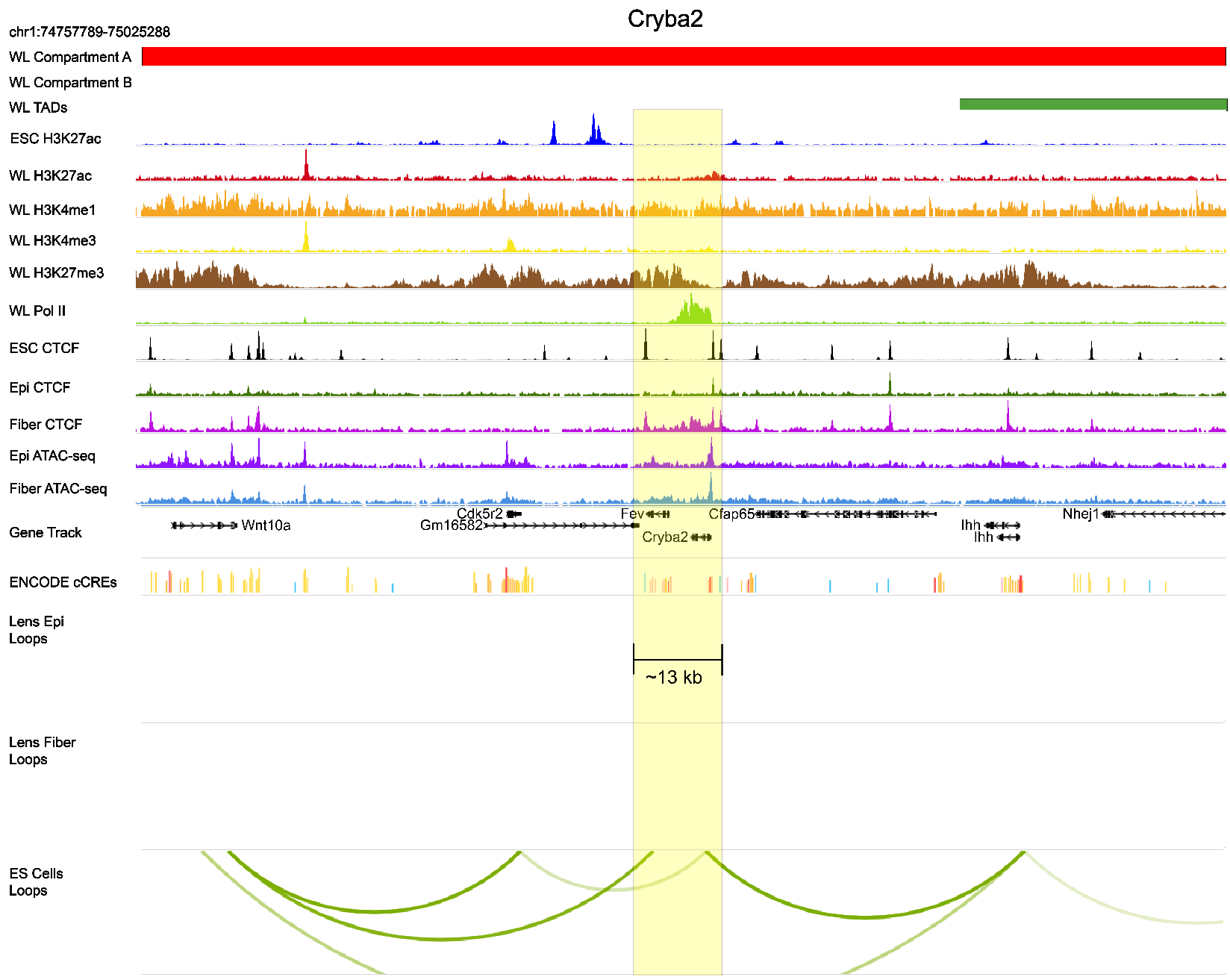


**Fig. S25: Chromatin loops, CTCF binding and other features of the Cryba2 locus.**

The Cryba2 locus (yellow box) is also marked by absence of large looping systems. Both lens epithelium and fiber share multiple similar CTCF peaks closely upstream of the Cryba2 transcriptional start site. Lens fiber cells show broad CTCF binding throughout the gene body with an overlap with RNA Polymerase II. See Fig. 10 for individual track description.


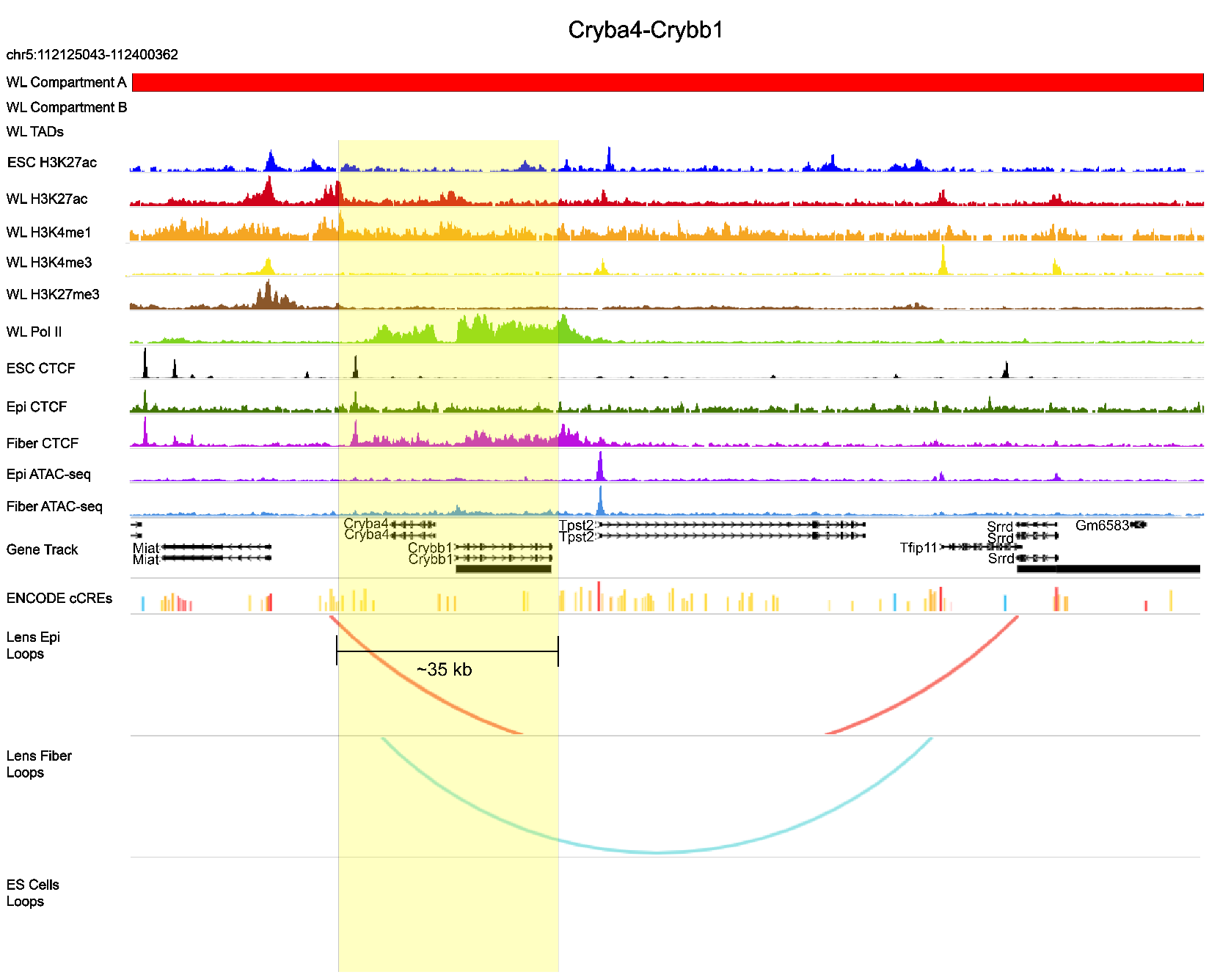


**Fig. S26: Chromatin loops, CTCF binding and other features of the Cryba4-Crybb1 bi-directional loci.**

Cryba4 and Crybb1 crystallin genes (yellow box) are transcribed in a bi-directional manner in lens [119]. Both loci span ~25 kb and are not located within or near any upstream/downstream TADs. Both gene bodies have high RNA Polymerase II and H3K27ac broad regions in their intergenic region including their “head-to-head” promoters separated by 3.3 kb of DNA. Shared CTCF epithelium and fiber cell peaks are found ~10 kb downstream of Cryba4. Like in other crystallin loci, a broad CTCF domain is detected across both gene bodies in fiber cell chromatin. See Fig. 10 for individual track description.


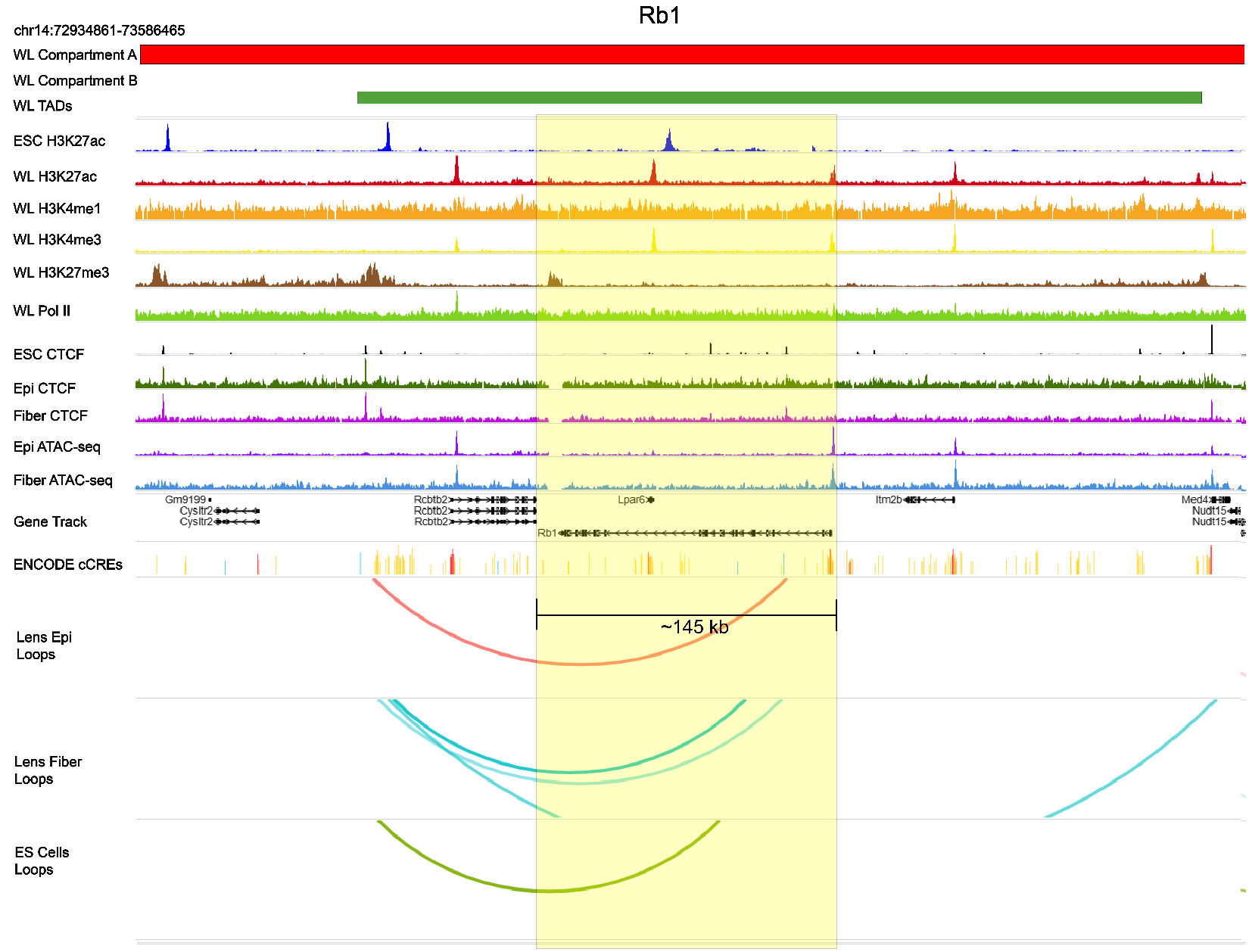


**Fig. S27: Chromatin loops, CTCF binding and other features of the Rb1 locus.**

Depletion of Rb1 affects lens cell cycle exit and causing disruption in lens fiber cell formation. The Rb1 locus (yellow box) shows shared chromatin loop contacts between lens epithelium and fiber to an intronic CTCF-bound contact, spanning to a downstream CTCF-bound contact. See Fig. 10 for individual track description.
