# Supplementary material for: Analysis of long-range chromatin contacts, compartments and looping between mouse embryonic stem cells, lens epithelium and lens fibers
Source: Epigenetics Chromatin. 2024 Apr 20;17:10. doi: 10.1186/s13072-024-00533-x (PMC11031936; doi:10.1186/s13072-024-00533-x)
Supplement: Supplementary file 8 — Supplementary Material 8 [file 13072_2024_533_MOESM8_ESM.docx]

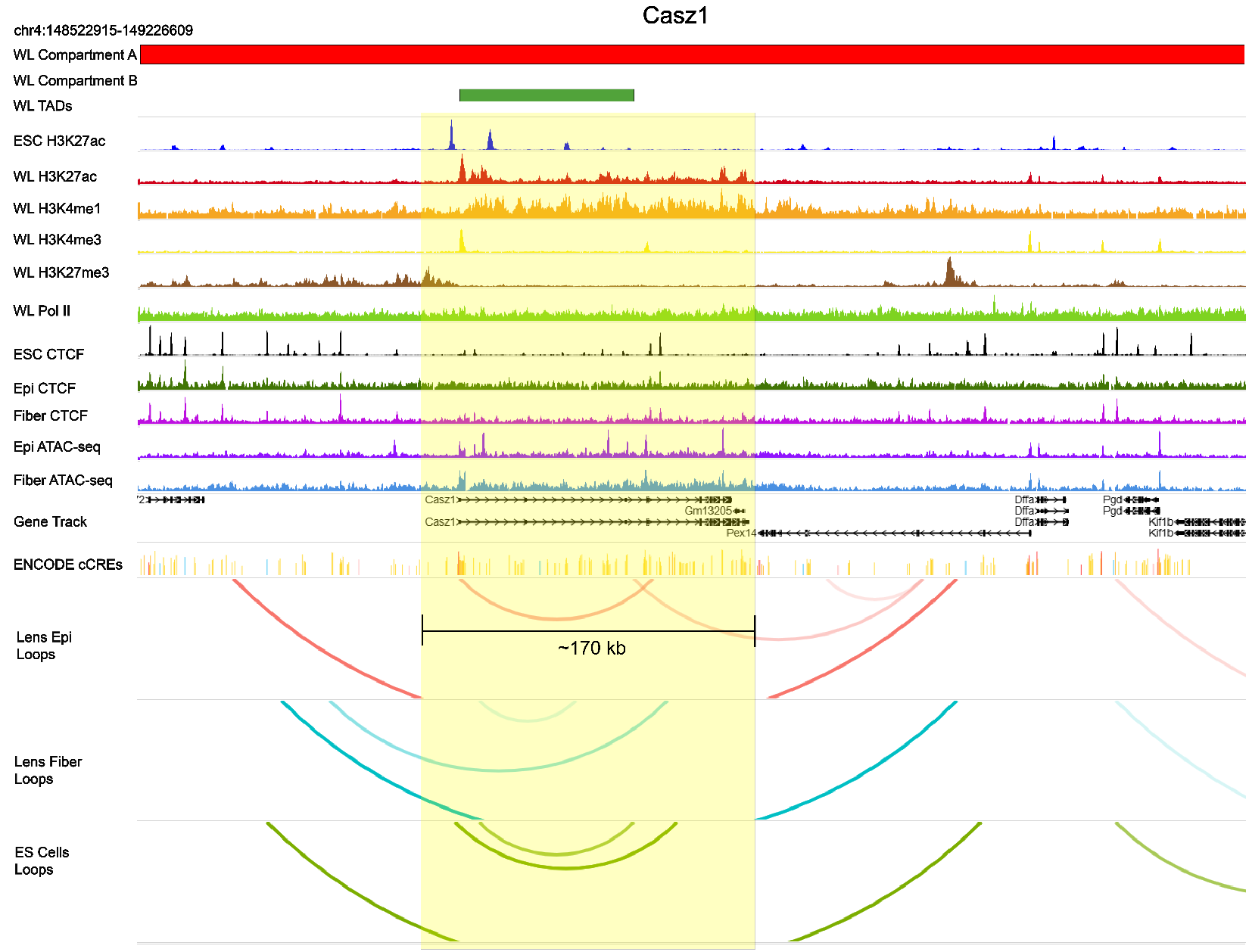


**Fig. S28: Chromatin loops, CTCF binding and other features of the Casz1 locus.**

Casz1 is a zinc finger transcription factor that is implicated lens fiber development. Multiple variants of CASZ1 have been shown to be associated with cataract formation. Lens epithelial, lens fiber, and ES cells all show a similar sized loop spanning the gene body making distal contacts both upstream and downstream of the gene. All three cell types have loop contacts within intronic elements of the gene. See Fig. 10 for individual track description.

**
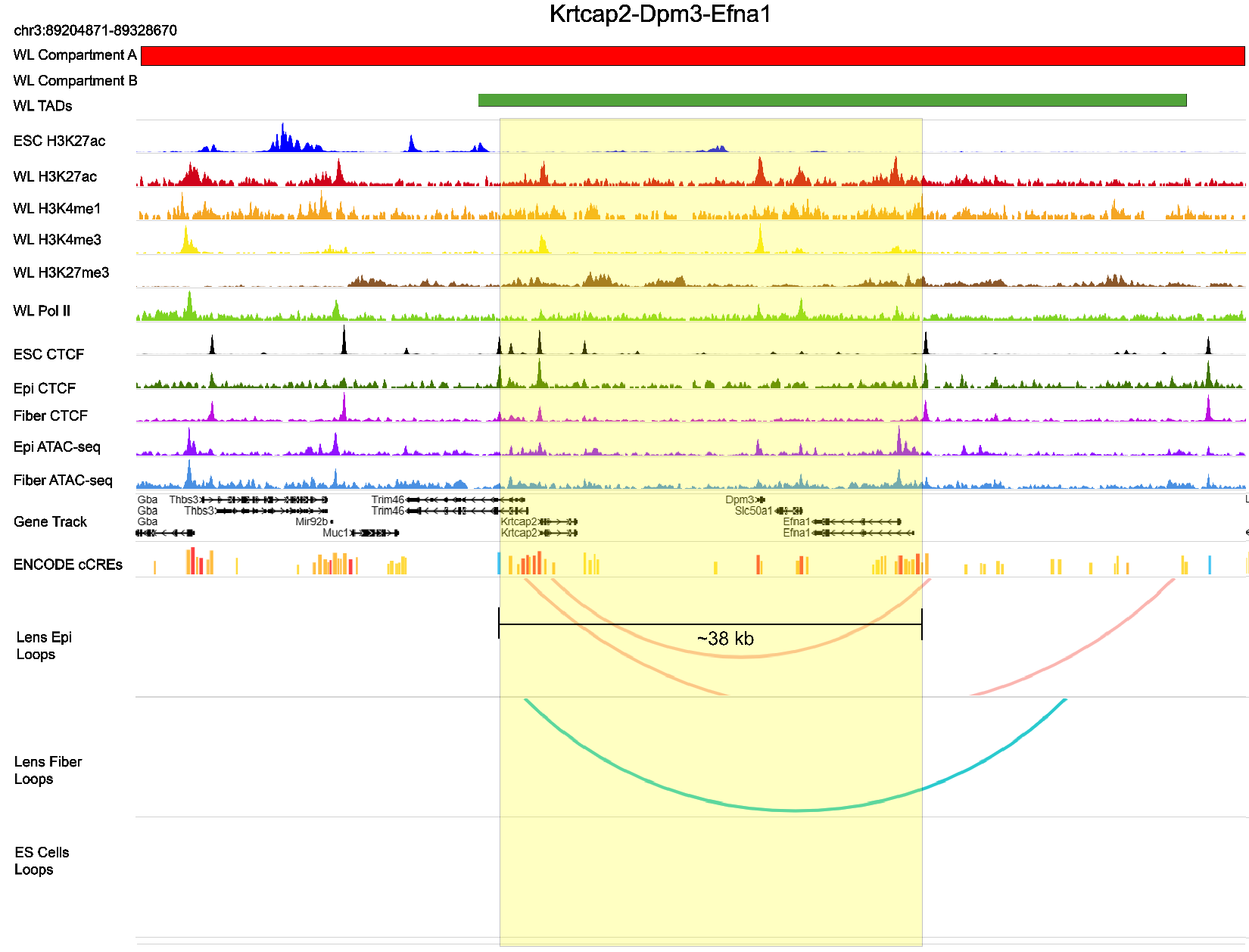
**

**Fig. S29: Chromatin loops, CTCF binding and other features of the Krtcap2-Dpm3-Efna1 gene cluster.**

The KRTCAP2-DPM3-EFNA1 cataract-associated gene cluster is shown in syntenic region of the mouse genome (yellow box) and shows markedly different loop patterns between lens epithelium and fiber cells. Loop structures are completely absent in ES cells. Both lens epithelium and lens fiber cells share the same loop contact at a promoter cCRE upstream of Krtcap2 but make different downstream distal contacts. See Fig. 10 for individual track description.

**
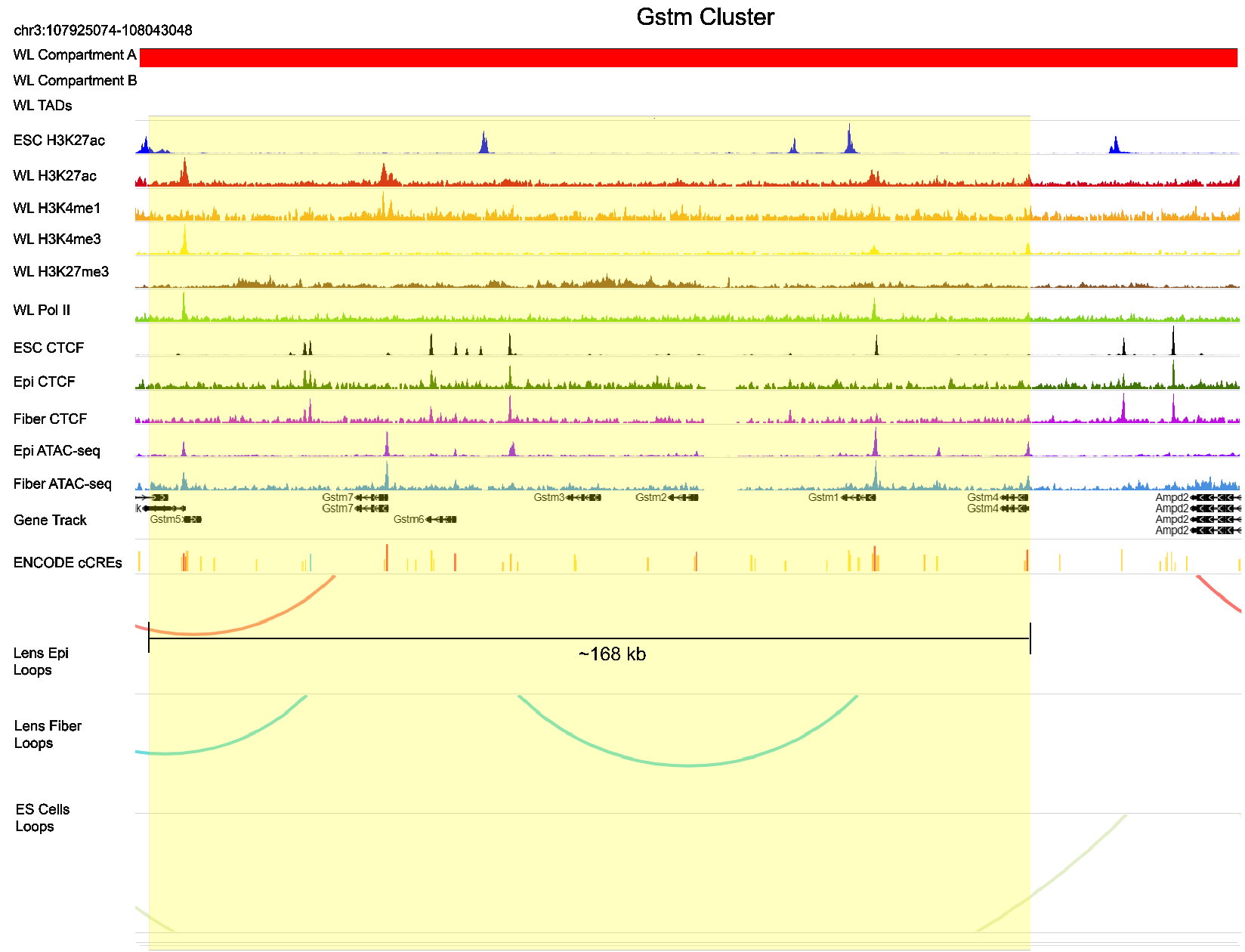
**

**Fig. S30: Chromatin loops, CTCF binding and other features of the Gstm cluster.**

The Gstm cluster (yellow box) consists of 7 paralogs within a 280 kb region. Mutations in human GSTM2 have been significantly linked to cataract formation in women. The Gstm cluster in lens epithelial cells is void of large loops. In lens fiber cells, a chromatin loop spans the center of the cluster and makes a CTCF-bound contact 9 kb downstream of Gstm3. In ES cells, a loop spans the entire Gstm cluster. See Fig. 10 for individual track description.

**
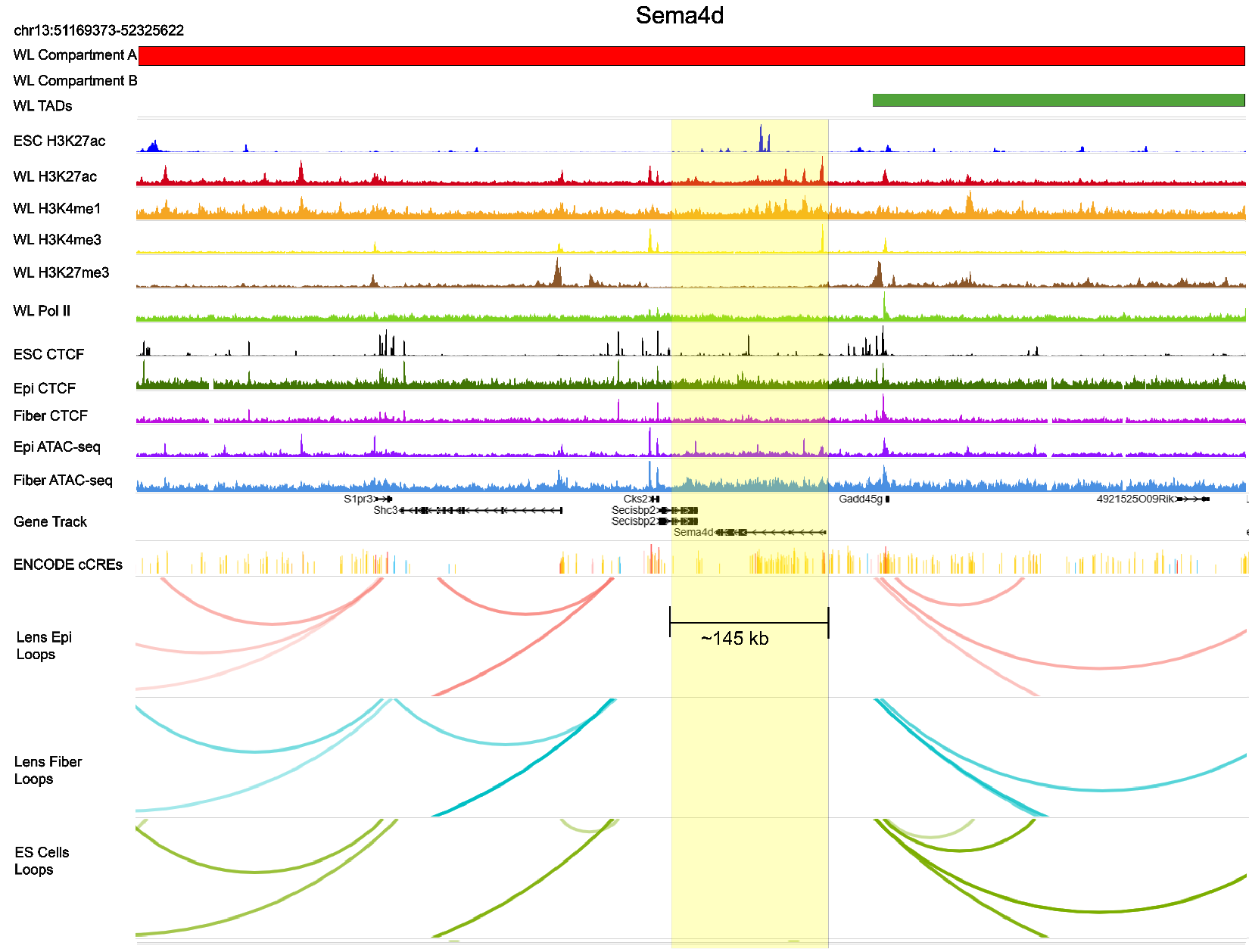
**

**Fig. S31: Chromatin loops, CTCF binding and other features of the Sema4d locus.**

Mutations in Semad4d have been significantly linked to cataract formation in men. The Sema4d locus (yellow box) is located within a region void of large chromatin looping with CTCF-bound chromatin both up and downstream of the gene. See Fig. 10 for individual track description.
