# Supplementary material for: Analysis of long-range chromatin contacts, compartments and looping between mouse embryonic stem cells, lens epithelium and lens fibers
Source: Epigenetics Chromatin. 2024 Apr 20;17:10. doi: 10.1186/s13072-024-00533-x (PMC11031936; doi:10.1186/s13072-024-00533-x)
Supplement: Supplementary file 9 — Supplementary Material 9 [file 13072_2024_533_MOESM9_ESM.docx]

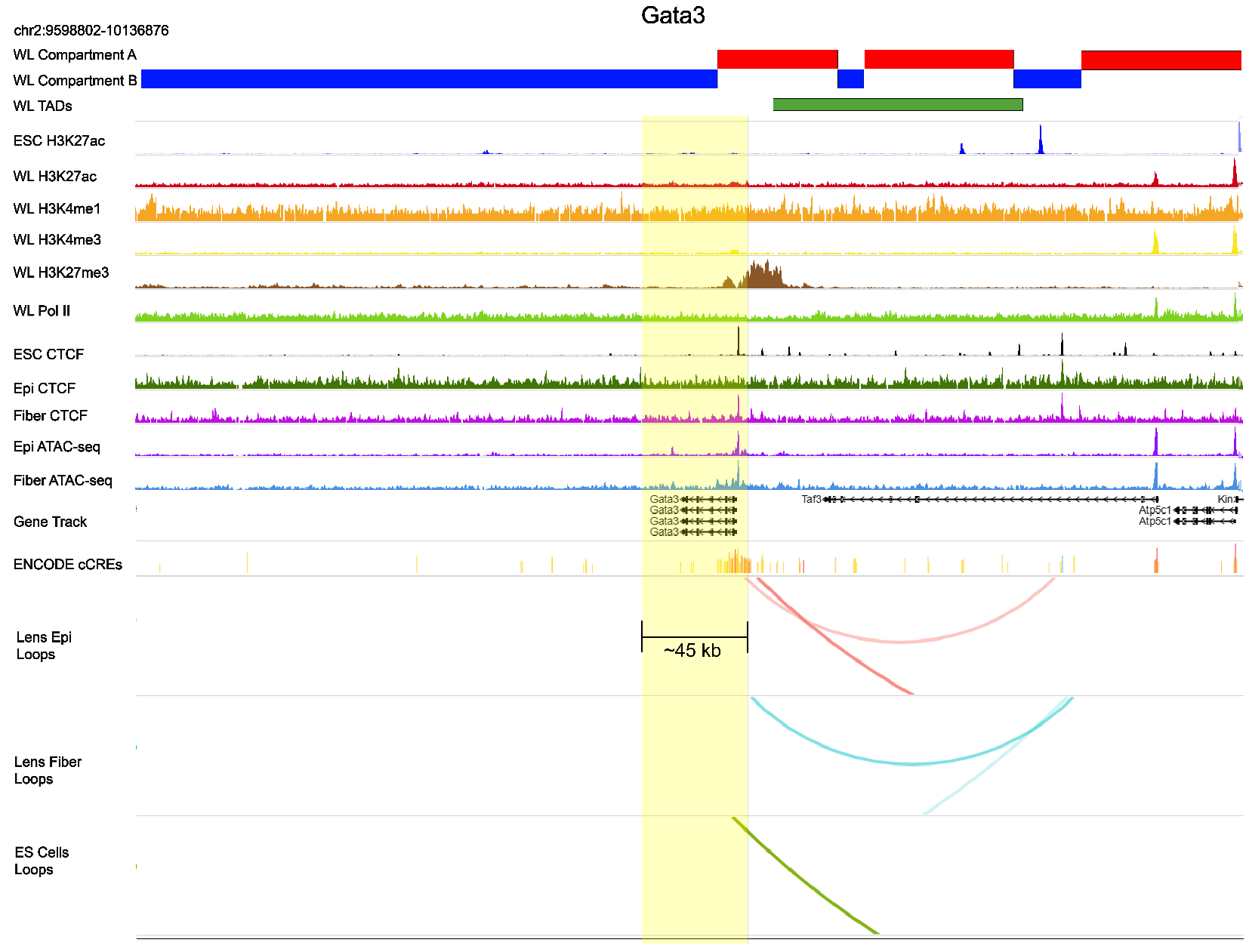


**Fig. S12: Chromatin loops, CTCF binding and other features of the Gata3 locus.**

Gata3 is a zinc finger-containing DNA binding transcription factor that is involved in primary lens fiber differentiation through the regulation of cell-cycle exit. The Gata3 locus (yellow box) has chromatin looping near the proximal 5’ end of the gene, contacting a CTCF-bound site. ES cells have a similar proximal contact but make further upstream distal contact. See Fig. 10 for individual track description.

**
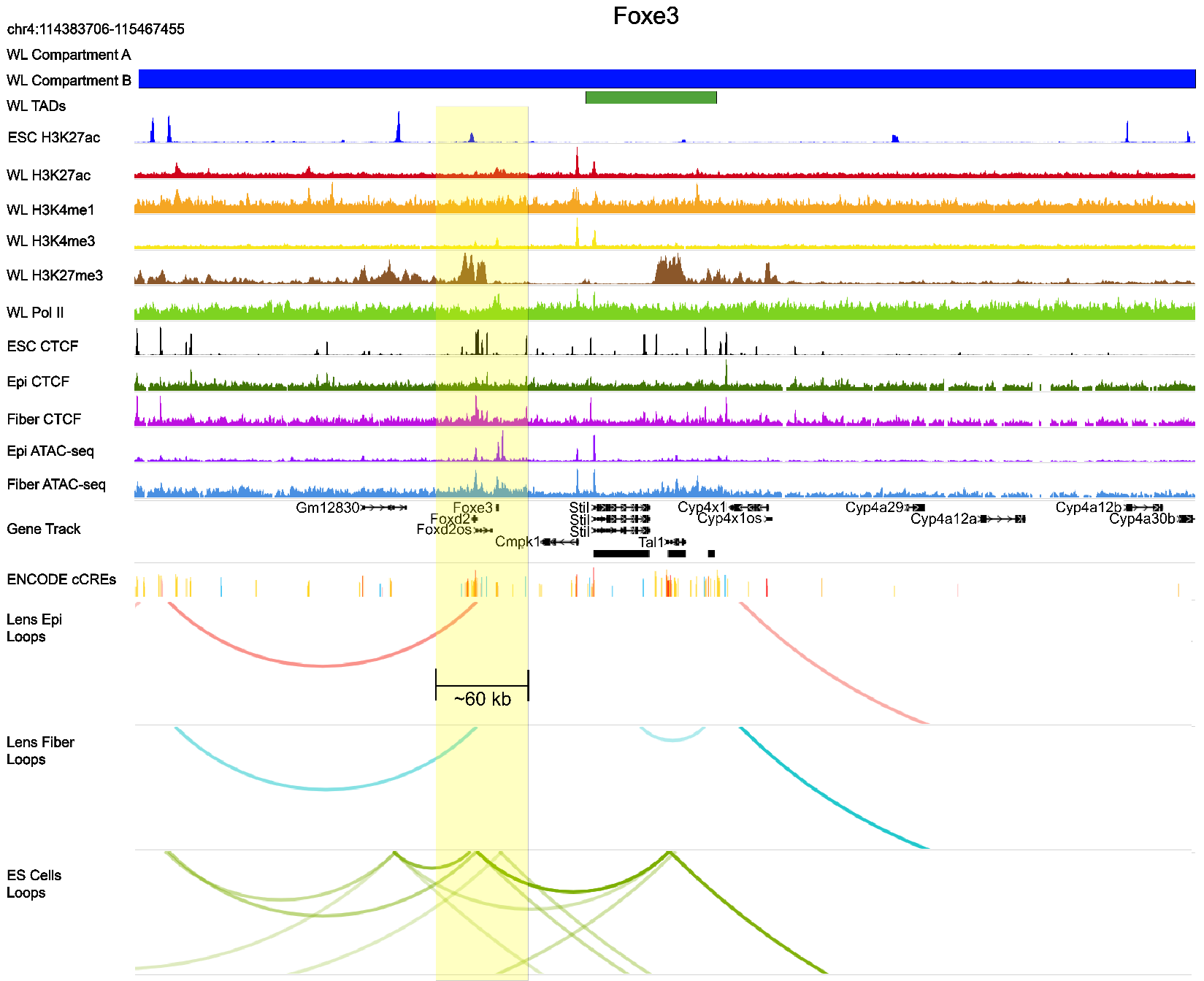
**

**Fig. S13: Looping patterns, CTCF binding and other features of the Foxe3 locus.**

Foxe3 encodes DNA-binding transcription regulating formation of the lens epithelium and other features of lens differentiation. The Foxe3 locus (yellow box) shows no large chromatin looping structures near the proximal regions of the gene body. Chromatin looping structures that spanned the Foxe3 locus are notably more complex in ES cells compared to lens cells. See Fig. 10 for individual track description.

**
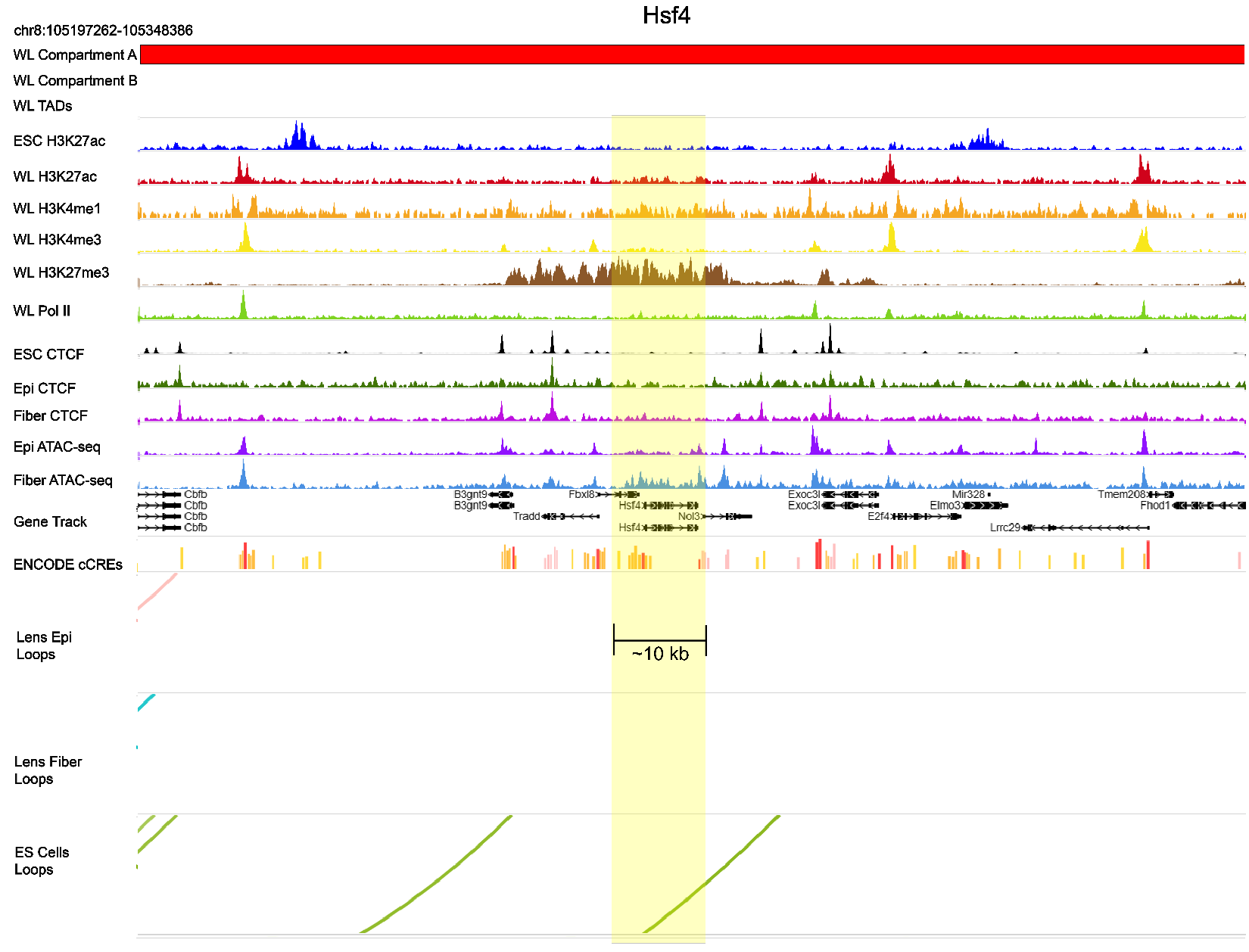
**

**Fig. S14: Chromatin loops, CTCF binding and other features of the Hsf4 locus.**

Hsf4 is a heat-shock transcription factor that plays a major role in organelle degradation via direct regulation of DNase IIβ and formation or the organelle-free zone. The Hsf4 locus (yellow box) shows no large proximal chromatin contacts. ES cell data shows two loop contacts at CTCF-bound chromatin. See Fig. 10 for individual track description.

**
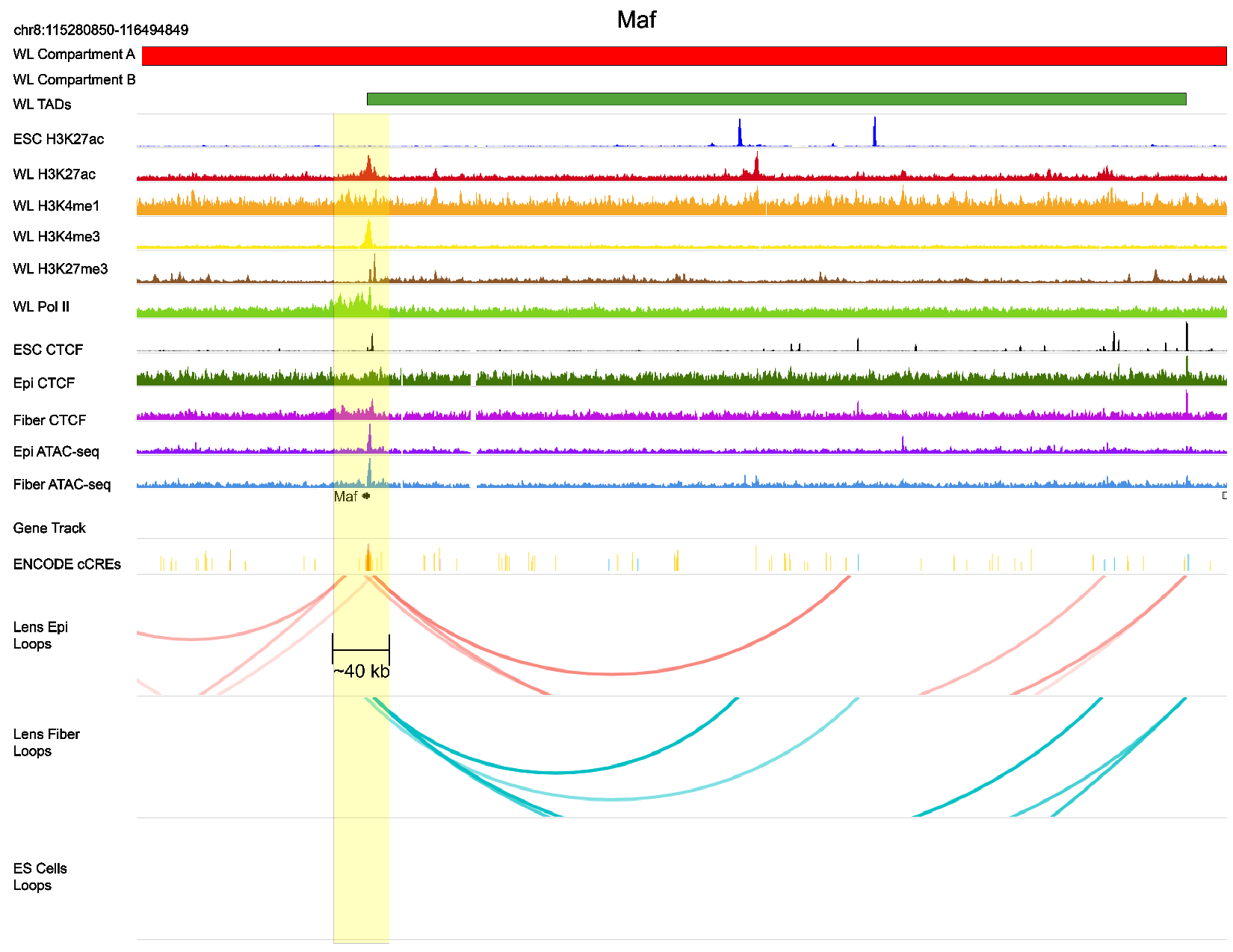
**

**Fig. S15: Chromatin loops, CTCF binding and other features of the Maf locus.**

Maf is a basic-leucine zipper containing transcription factor that is involved in crystallin production and is a downstream target of Pax6. The Maf locus (yellow box) shows similar upstream distal chromatin loop contacts in both lens epithelium and lens fiber cells bound by CTCF. Lens epithelium has unique distal contacts downstream of the locus. ES cell data is absent of loops within the Maf locus. See Fig. 10 for individual track description.

**
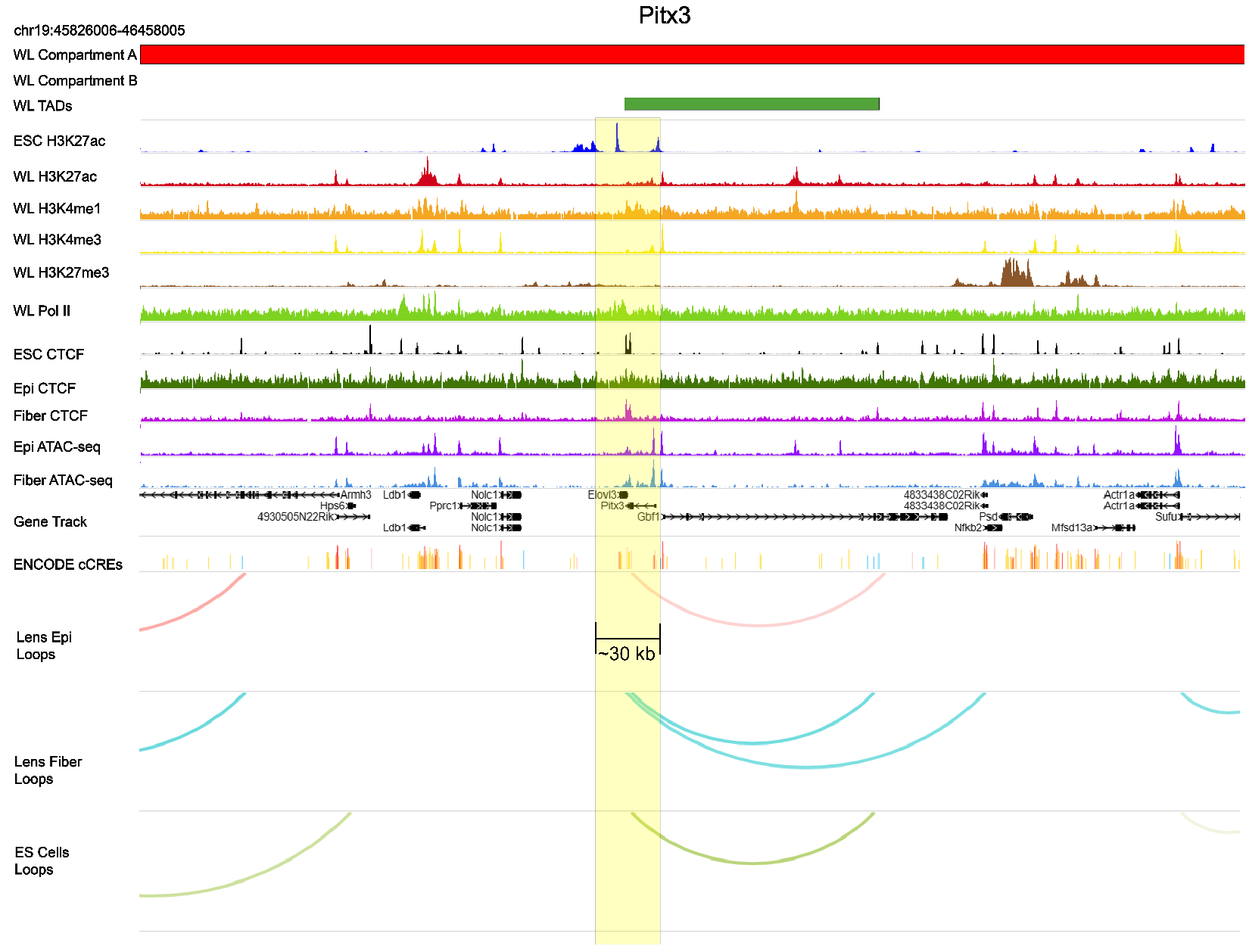
**

**Fig. S16: Chromatin loops, CTCF binding and other features of the Pitx3 locus.**

Pitx3 is a DNA binding transcription factor and directly regulates Foxe3. Pitx3 is implicated lens maturation and cataract formation. The Pitx3 locus (yellow box) has a loop that spans the gene body and makes an upstream contact bound by CTCF in lens epithelium, lens fiber, and ES cells. See Fig. 10 for individual track description.

**
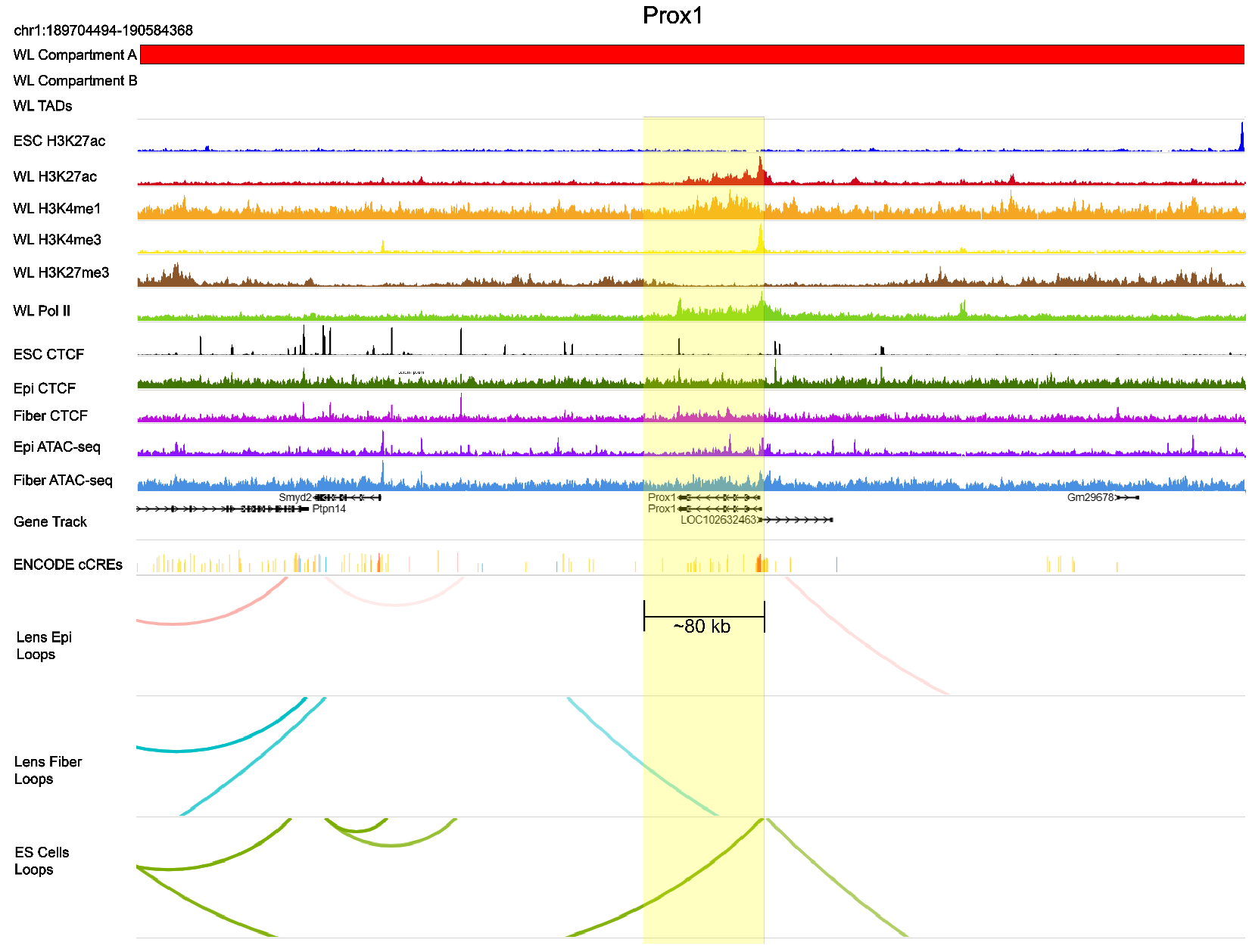
**

**Fig. S17: Chromatin loops, CTCF binding and other features of the Prox1 locus.**

Prox1 is a homeodomain-containing DNA binding transcription that is a direct target of Pax6 and regulator of crystalline gene expression. Prox1 is implicated in lens fiber cell maturation through mediation of FGF signaling. Lens fiber and lens epithelial cells have distinct differences in chromatin loop structure near the Prox1 locus (yellow box), showing a proximal upstream contact in lens epithelium and a downstream contact bound to CTCF in lens fiber cells. See Fig. 10 for individual track description.

**
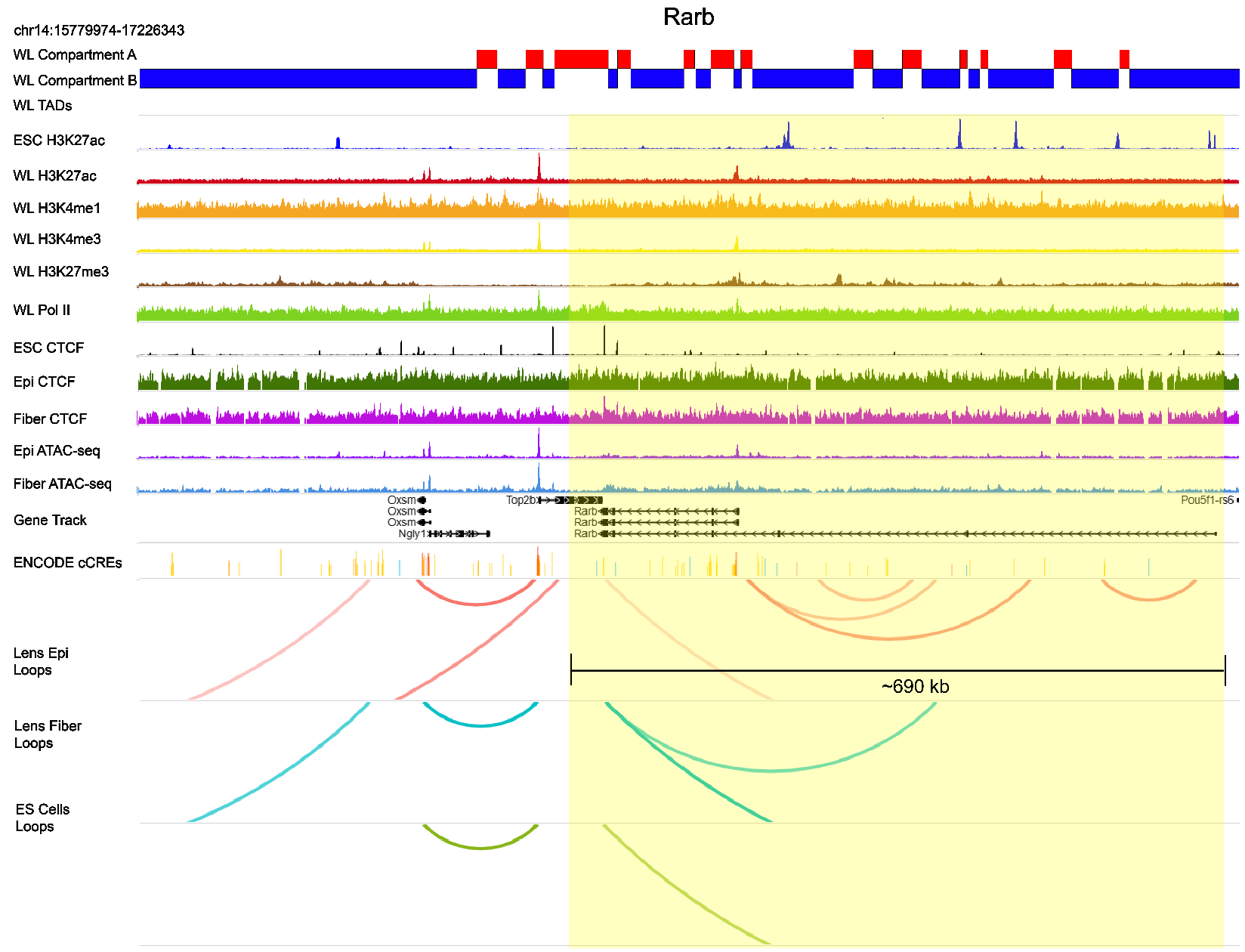
**

**Fig. S18: Chromatin loops, CTCF binding and other features of the Rarb locus.**

Rarb is a transcription factor that is involved in pan-ocular developmental processes regulated by retinoic acid signaling. Mutations in Rarb are associated with microphthalmia, anophthalmia and coloboma. The Rarb locus (yellow box) show a complex network of loops upstream of the TSS in lens epithelium, with fewer contacts in lens fiber cells. Lens epithelium, lens fiber, and ES cells share the same loop contact near the TTS. See Fig. 10 for individual track description.

**
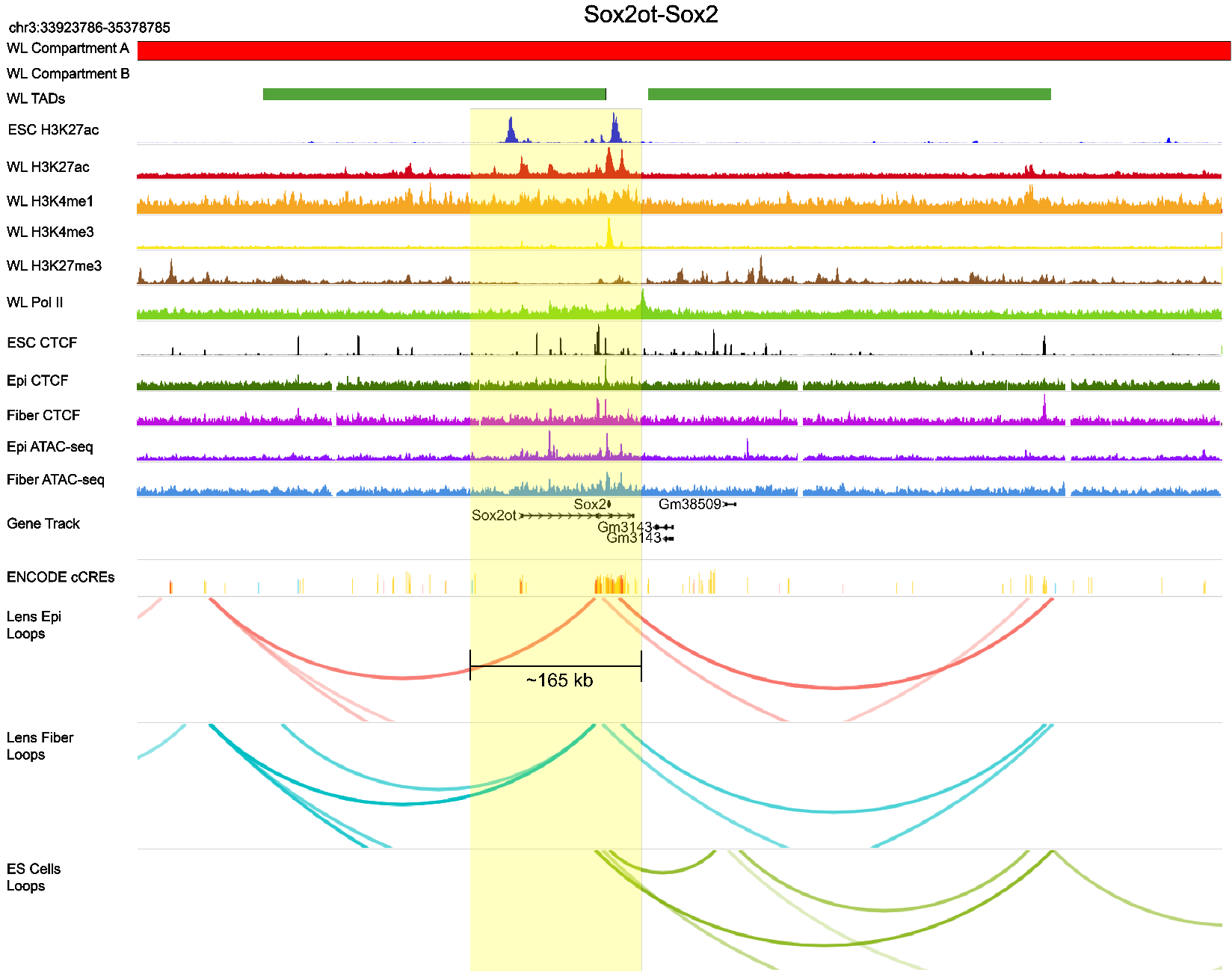
**

**Fig. S19: Chromatin loops, CTCF binding and other features of the Sox2ot-Sox2 locus.**

The transcription factor Sox2 controls pluripotency of ES cells and regulates early stages of lens embryogenesis. Thus, marked differences in looping patterns are found between ES and lens cells. The ES cells show no chromatin loop structures upstream of the Sox2 locus when compared to multiple nested loop structures in lens epithelium and fiber cells. Lens epithelium and lens fiber cells share TADs of 355 kb and 420 kb in length, upstream and downstream respectively. Both cell types share a CTCF bound loop anchor ~2.5 kb upstream of the Sox2 TSS. See Fig. 10 for individual track description.


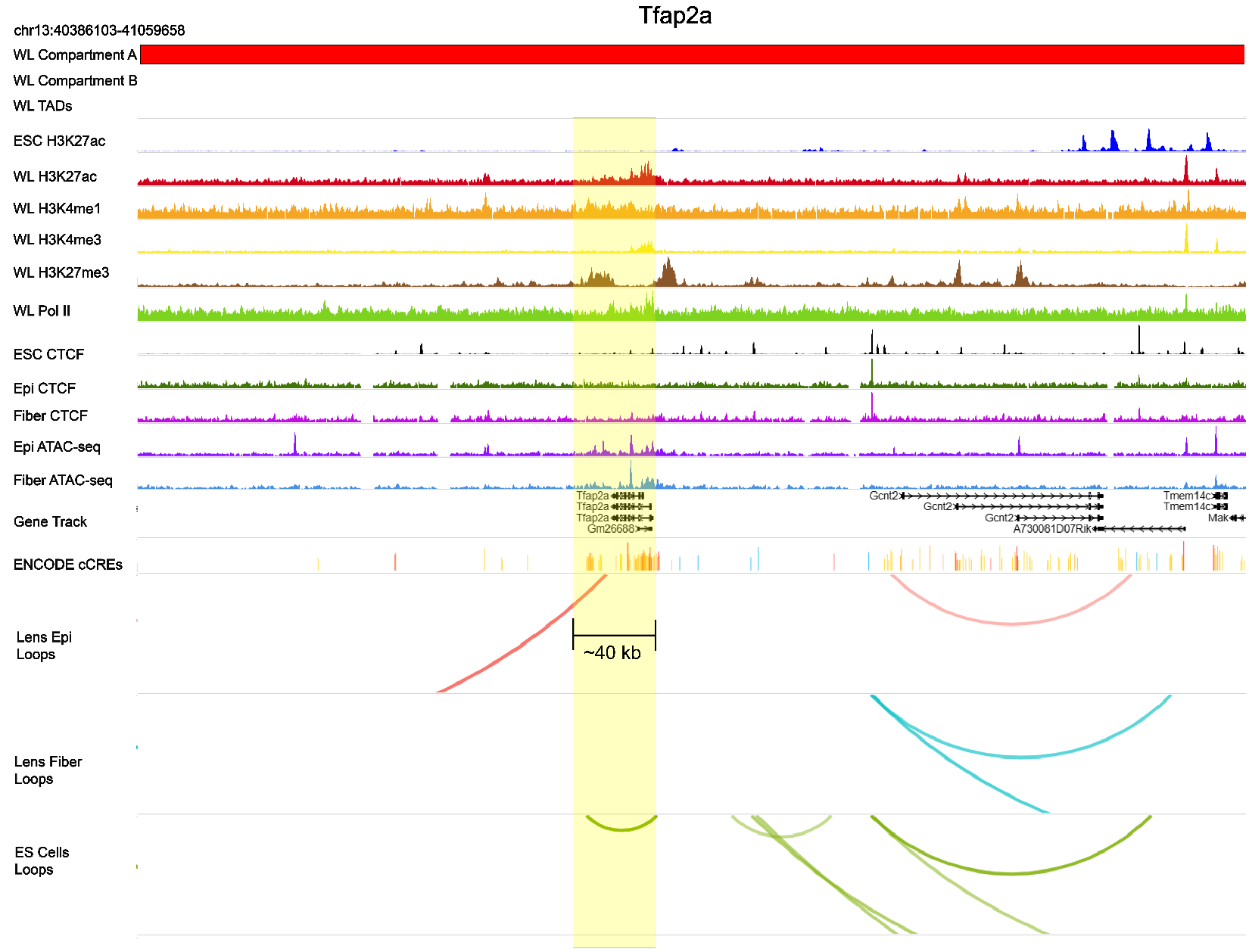


**Fig. S20: Chromatin loops, CTCF binding and other features of the Tfap2a locus.**

Tfap2a is a DNA-binding transcription factor implicated pan-ocular diseases, including lens malformations, retinal defects, and optic fissure defects. The Tfap2a locus (yellow box). Lens epithelial cells have a downstream contact near the TTS that spans the downstream intergenic region, whereas lens fiber cells have an absence of proximal loops. ES cells contain one loop that spans the Tfap2a locus, making contacts with both promoter and proximal enhancer cCREs. See Fig. 10 for individual track description.
