# Supplementary material for: Analysis of long-range chromatin contacts, compartments and looping between mouse embryonic stem cells, lens epithelium and lens fibers
Source: Epigenetics Chromatin. 2024 Apr 20;17:10. doi: 10.1186/s13072-024-00533-x (PMC11031936; doi:10.1186/s13072-024-00533-x)
Supplement: Supplementary file 10 — Supplementary Material 10 [file 13072_2024_533_MOESM10_ESM.docx]

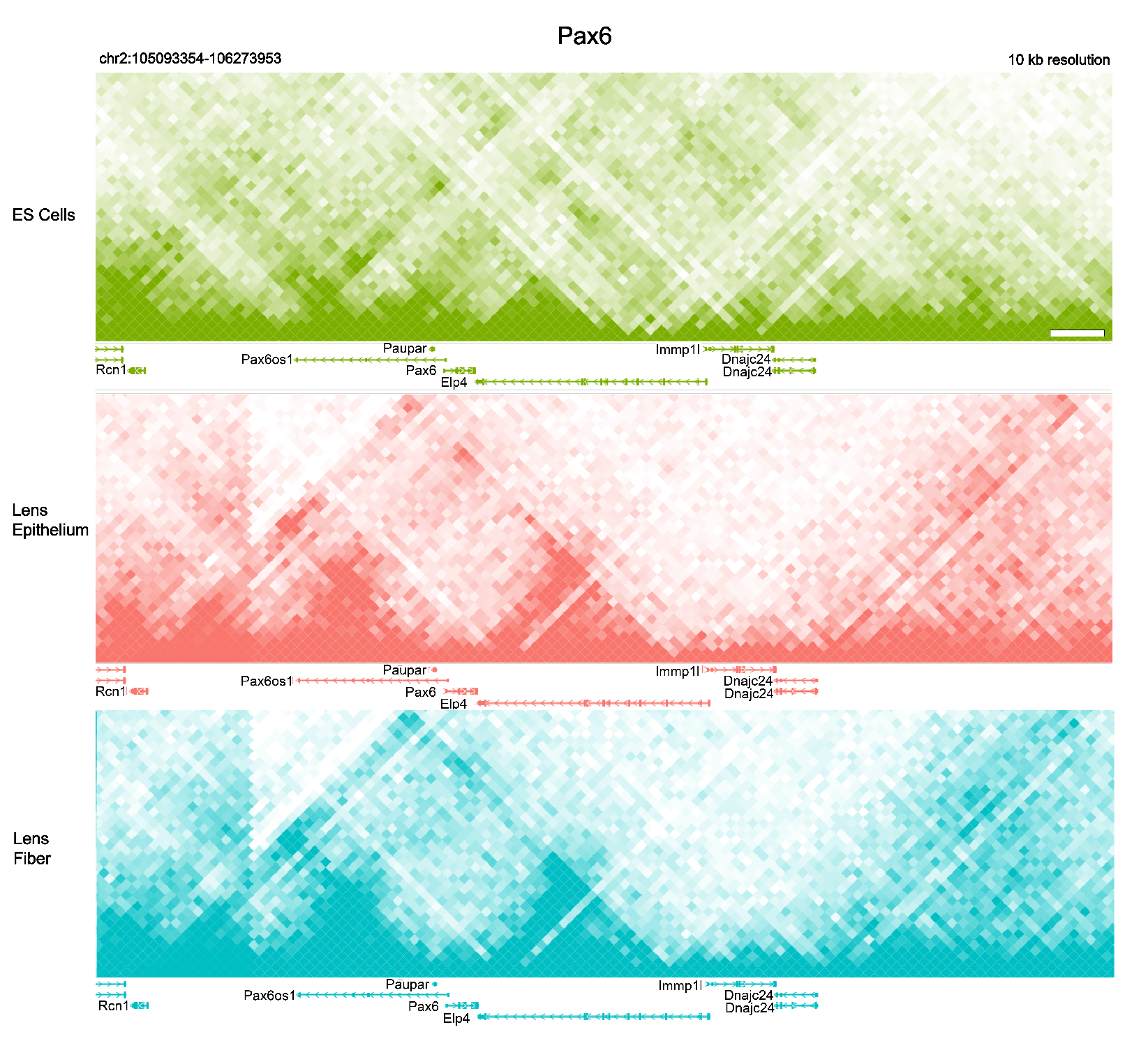
**Fig. S6: Hi-C contact map of *Pax6* locus in ES and lens cells**

Three separate contact maps shown at 10 kb resolution of the *Pax6* locus in ES, lens epithelium, lens fiber cells. Scale bar indicates 50 kb in length. For loops, TADs, and further annotations, see Fig. 10 in main text.


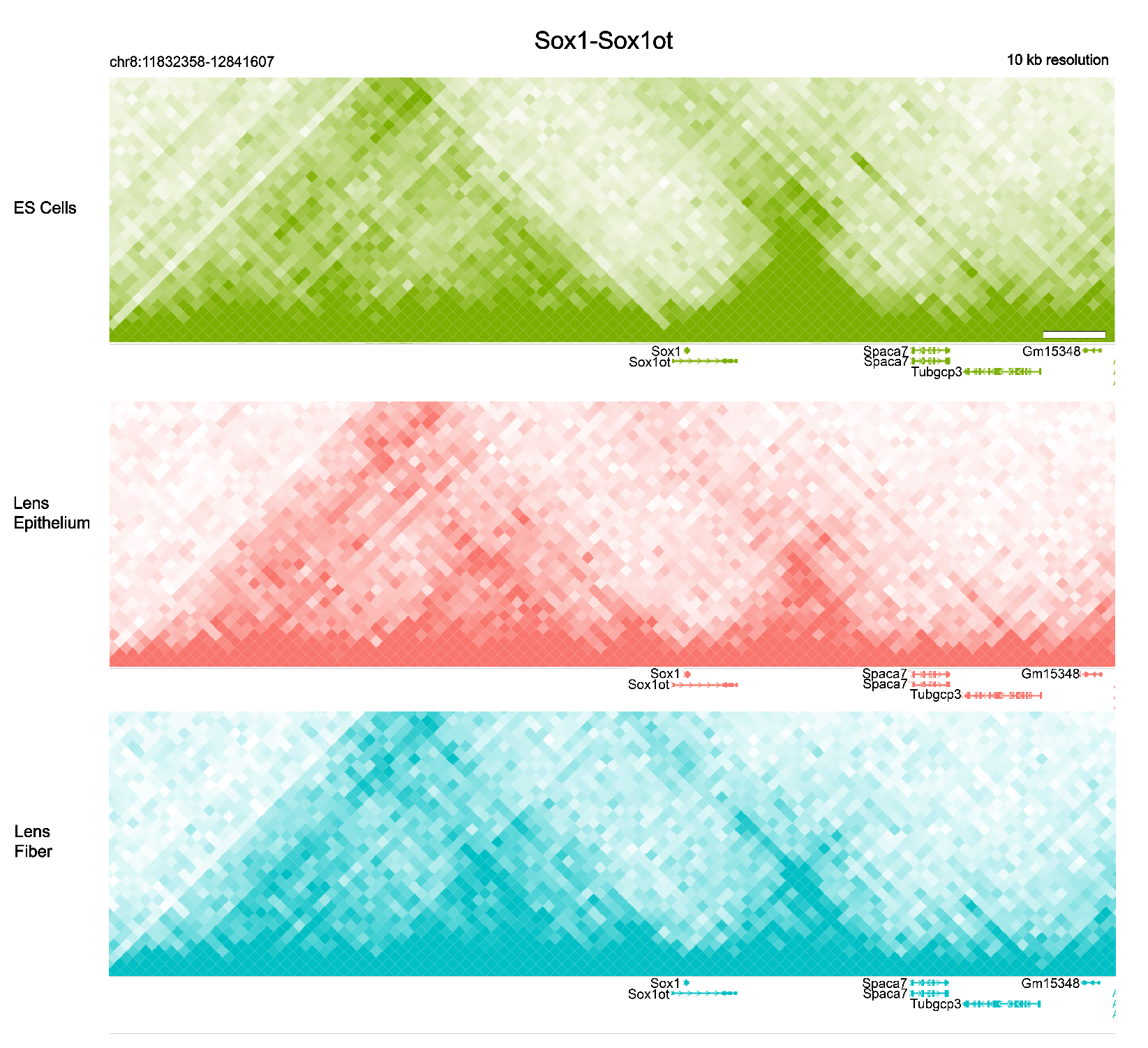


**Fig. S7: Hi-C contact map of Sox1-Sox1ot locus in ES and lens cells**

Three separate contact maps shown at 10 kb resolution of the Sox1-Sox1ot locus in ES, lens epithelium, lens fiber cells. Scale bar indicates 50 kb in length. For loops, TADs, and further annotations, see Fig. 11 in main text.


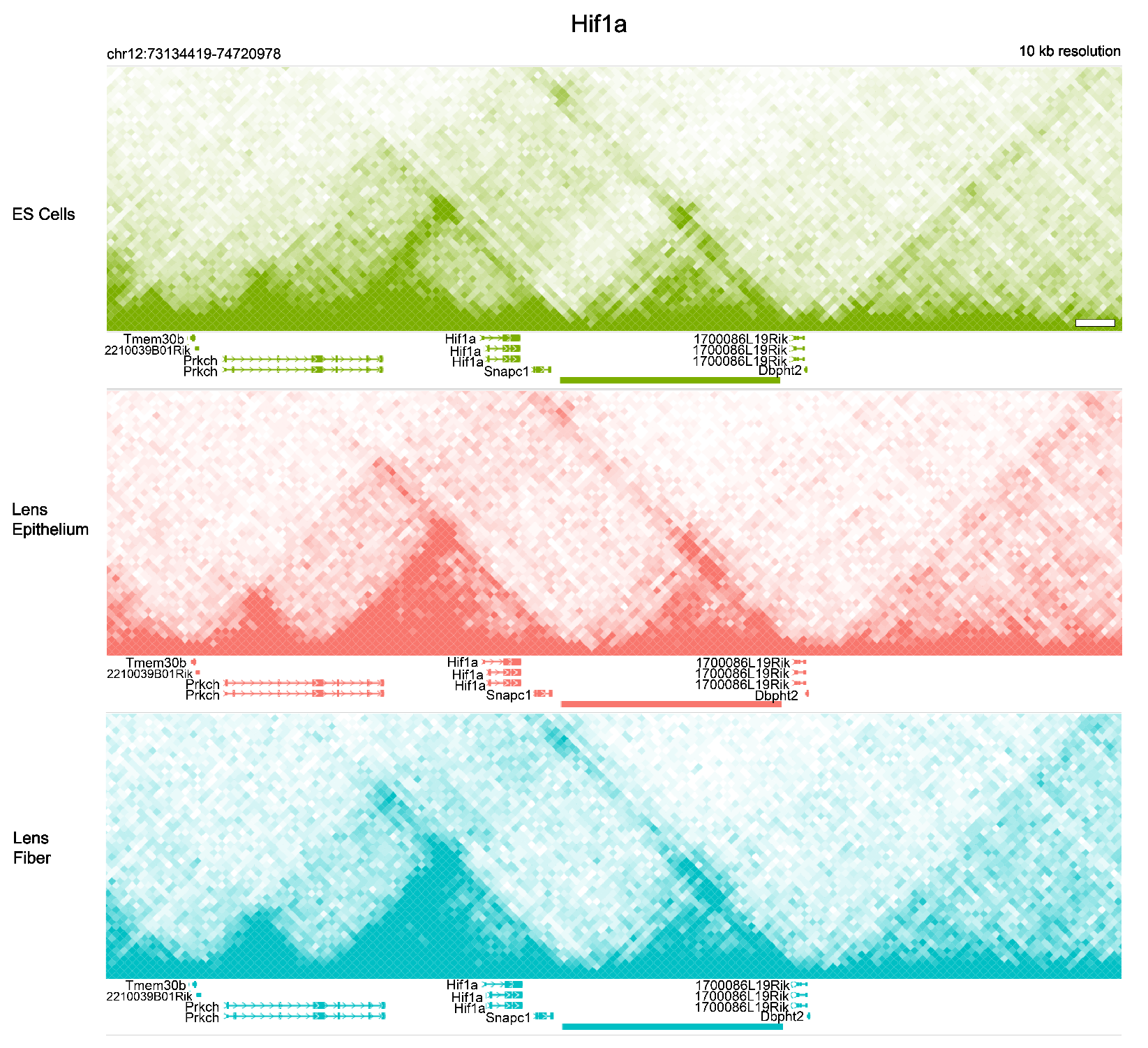


**Fig. S8: Hi-C contact map of Hif1a locus in ES and lens cells**

Three separate contact maps shown at 10 kb resolution of the Hif1a locus in ES, lens epithelium, lens fiber cells. Scale bar indicates 50 kb in length. For loops, TADs, and further annotations, see Fig. 12 in main text.


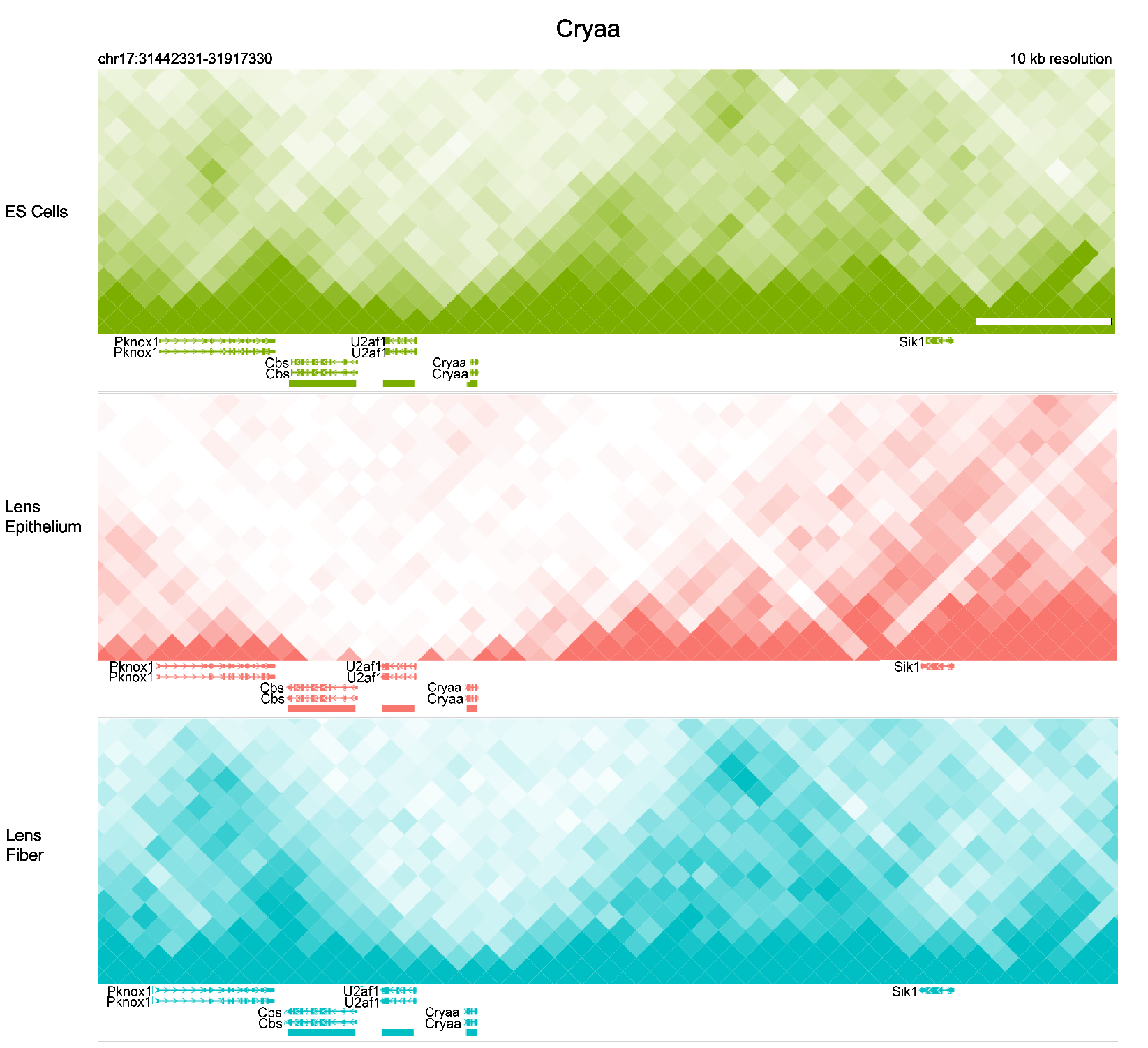


**Fig. S9: Hi-C contact map of Cryaa locus in ES and lens cells**

Three separate contact maps shown at 10 kb resolution of the Cryaa locus in ES, lens epithelium, lens fiber cells. Scale bar indicates 50 kb in length. For loops, TADs, and further annotations, see Fig. 13 in main text.


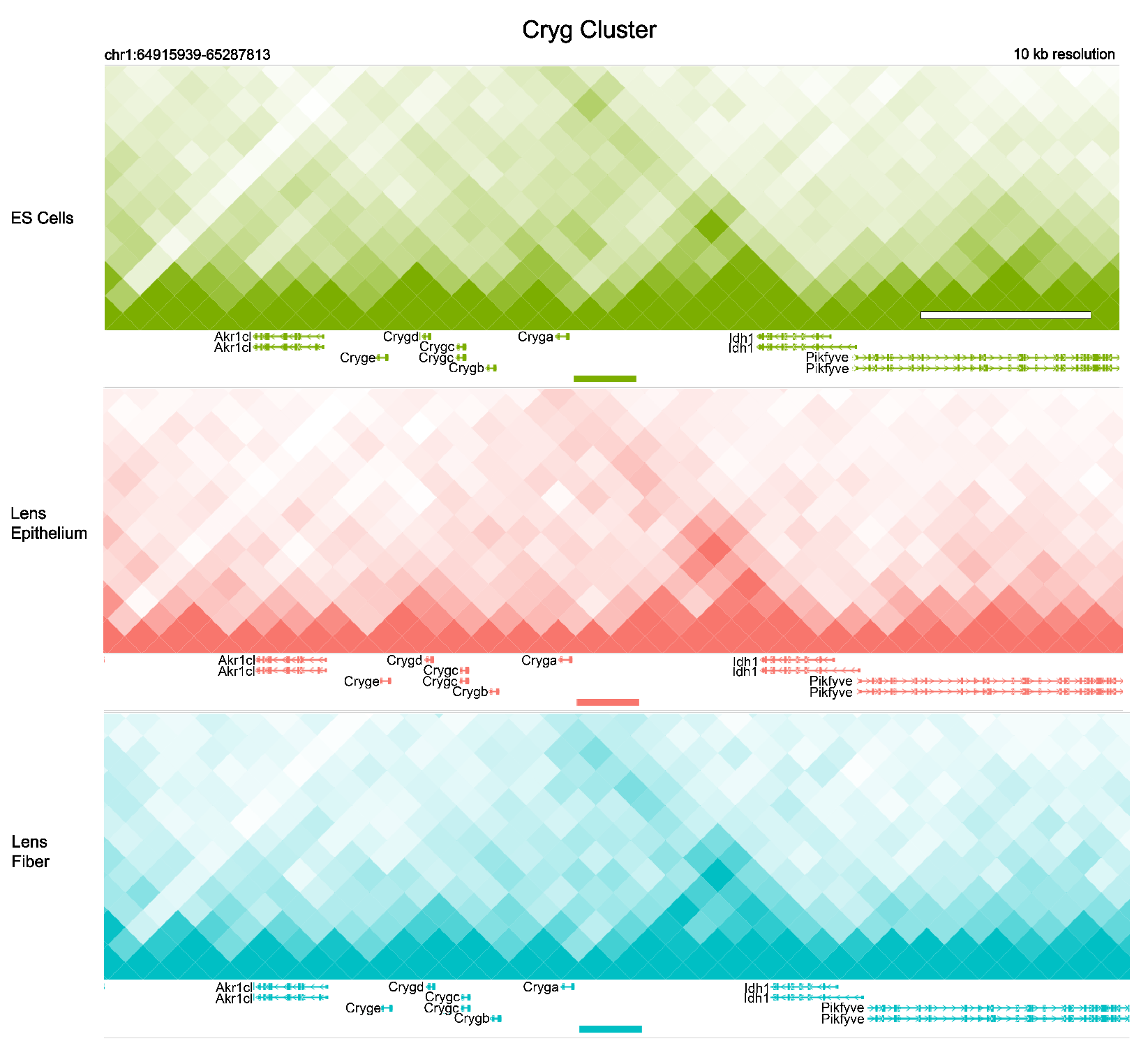


**Fig. S10: Hi-C contact map of Cryg cluster in ES and lens cells**

Three separate contact maps shown at 10 kb resolution of the Cryg cluster in ES, lens epithelium, lens fiber cells. Scale bar indicates 50 kb in length. For loops, TADs, and further annotations, see Fig. 14 in main text.


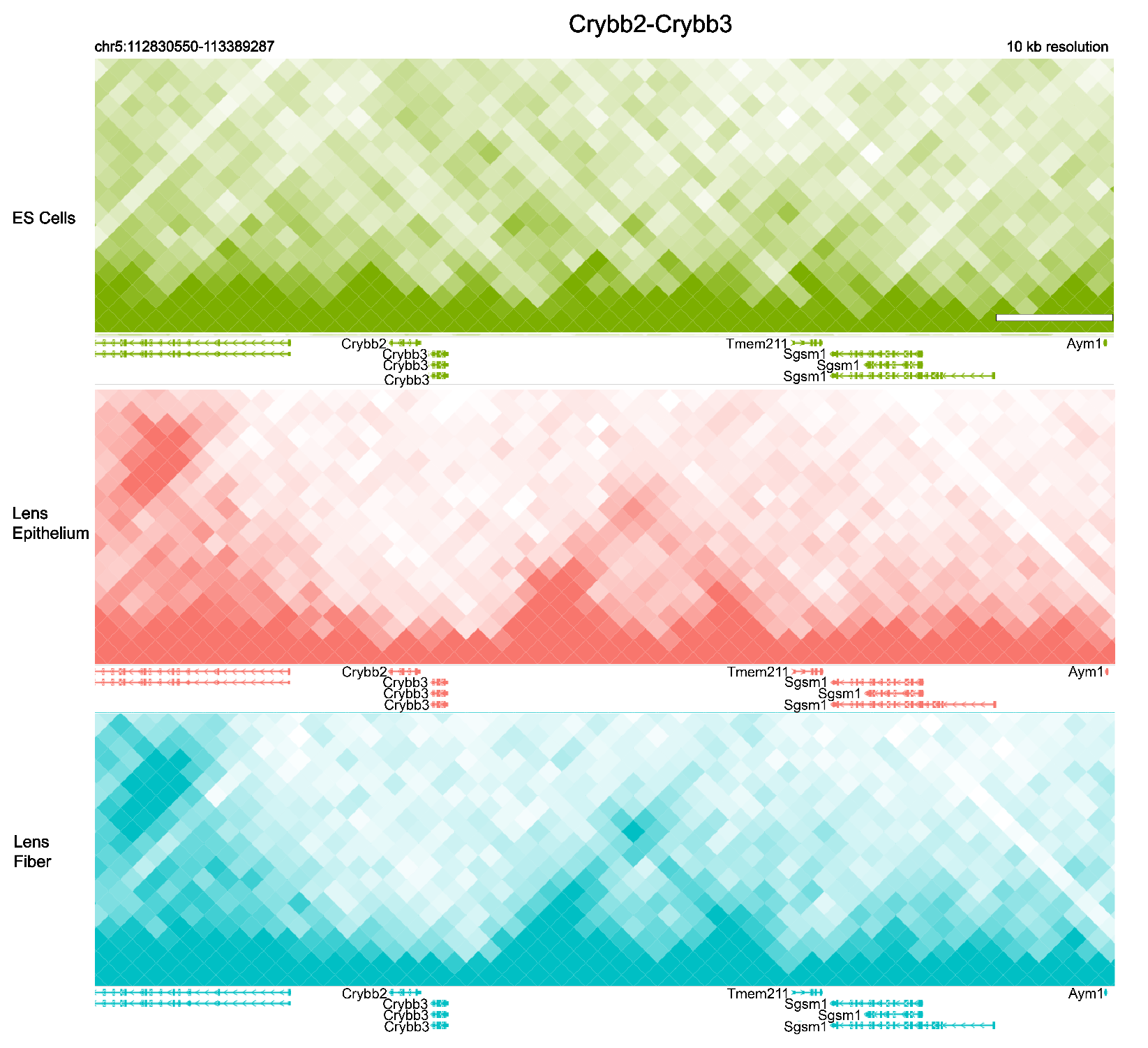


**Fig. S11: Hi-C contact map of Crybb2-Crybb3 locus in ES and lens cells**

Three separate contact maps shown at 10 kb resolution of the Crybb2-Crybb3 locus in ES, lens epithelium, lens fiber cells. Scale bar indicates 50 kb in length. For loops, TADs, and further annotations, see Fig. 15 in main text.
