# Supplementary material for: Analysis of long-range chromatin contacts, compartments and looping between mouse embryonic stem cells, lens epithelium and lens fibers
Source: Epigenetics Chromatin. 2024 Apr 20;17:10. doi: 10.1186/s13072-024-00533-x (PMC11031936; doi:10.1186/s13072-024-00533-x)
Supplement: Supplementary file 12 — Supplementary Material 12 [file 13072_2024_533_MOESM12_ESM.docx]

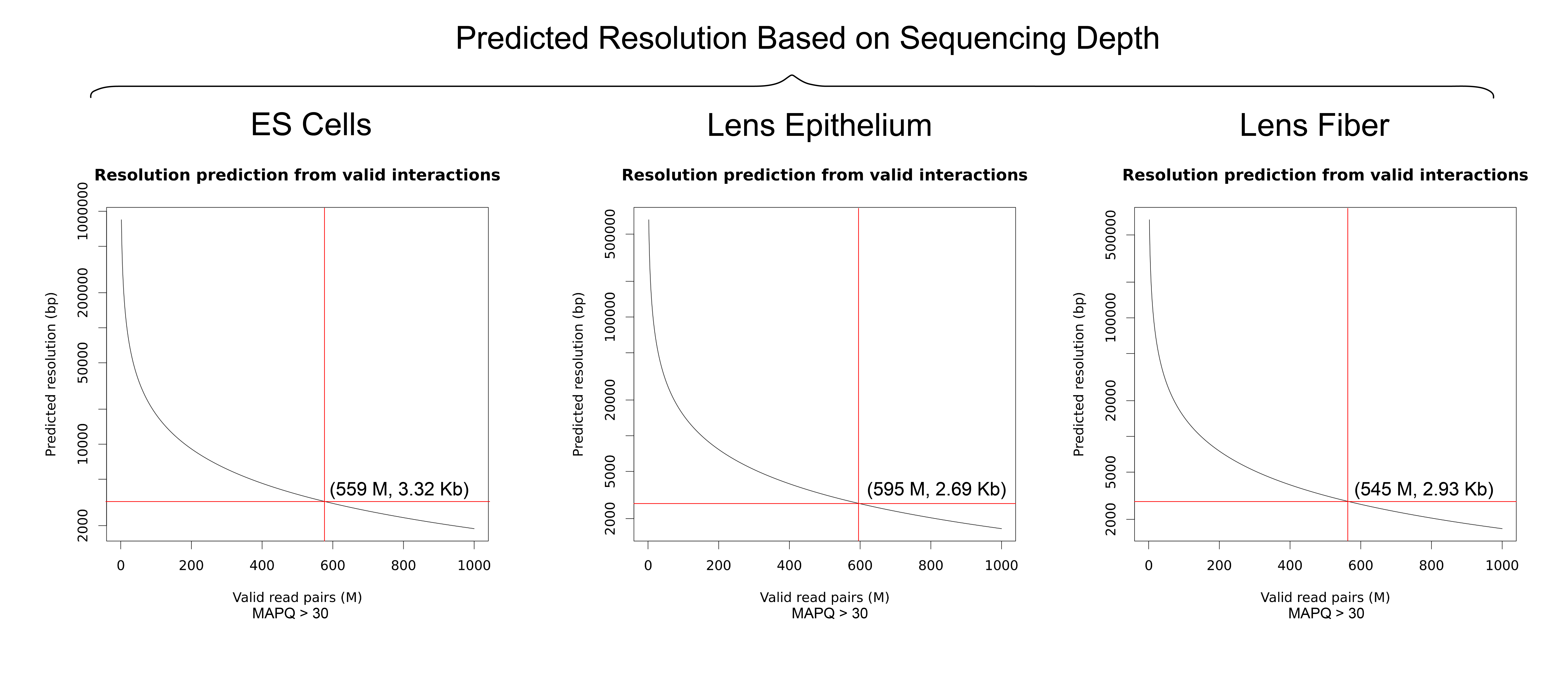


**Fig. S1: Predicted resolution of Hi-C contact maps.**

Predicted resolution of the HiC contact maps for ES cells, lens epithelium and lens fibers, calculated by HiCRes as described in Materials and Methods.





**Fig. S2: Mean loop and TAD size sorted by individual chromosomes.**

**a)** Average loop sizes by chromosome. A general trend of decreasing size based on chromosome length with exception of chromosome 2 and 13 was found. ES cells have significantly larger loop sizes than lens cells. **b)** Average TAD size by chromosome. Lens cells have larger TADs than ES cells. TAD size was less affected by chromosome length than loop size. For complete data and statistical comparisons, see Additional File 3: Table S2.


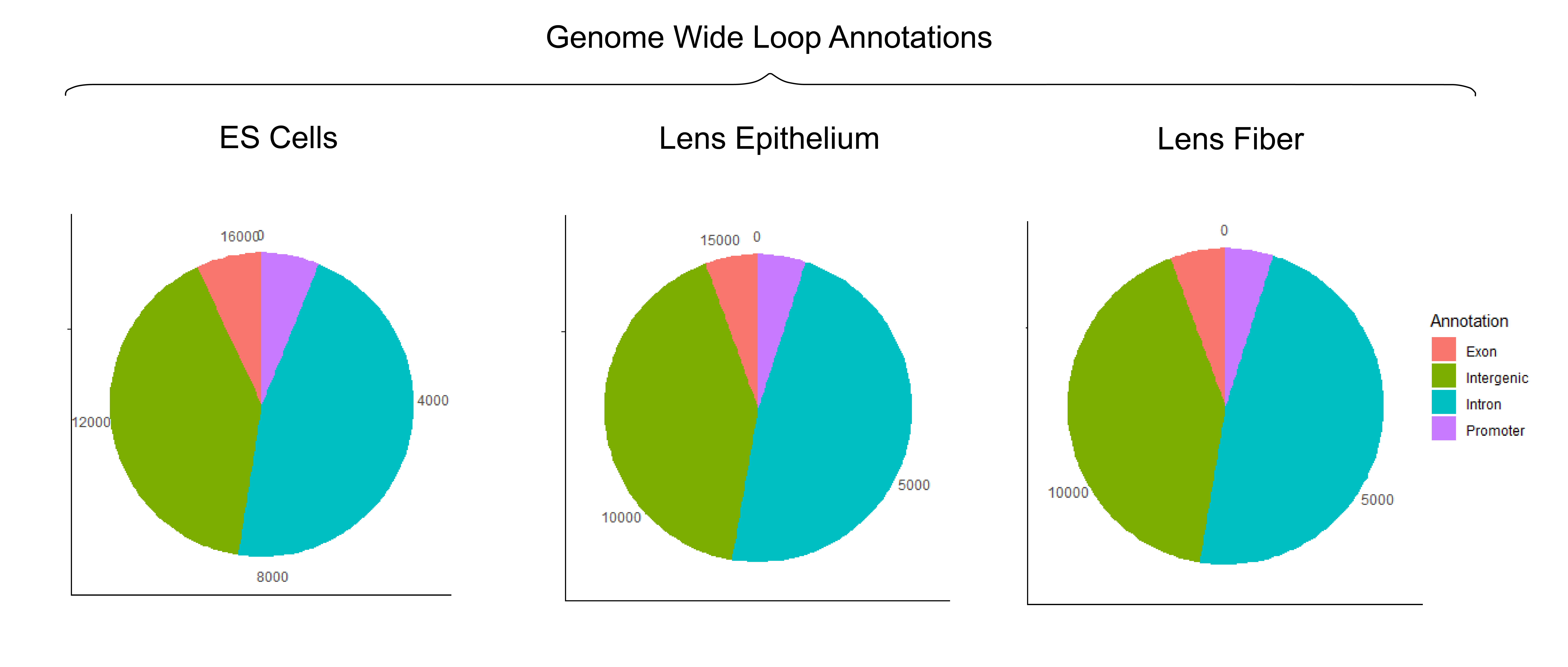


**Fig. S3: Genome-wide chromatin loop annotations.**

Loops anchors were annotated using HOMER annotate function to determine the proportion of loop anchors associated with gene structure. Overall proportions of genome wide chromatin loop annotations were similar between all three cell types examined. ES cells: exon = 1138, intergenic = 6618, intron = 7566, promoter = 1000. Lens epithelium: exon = 864, intergenic = 6481, intron = 7417, promoter = 776. Lens fiber cells: exon = 845, intergenic = 6210, intron = 7109, promoter = 722.


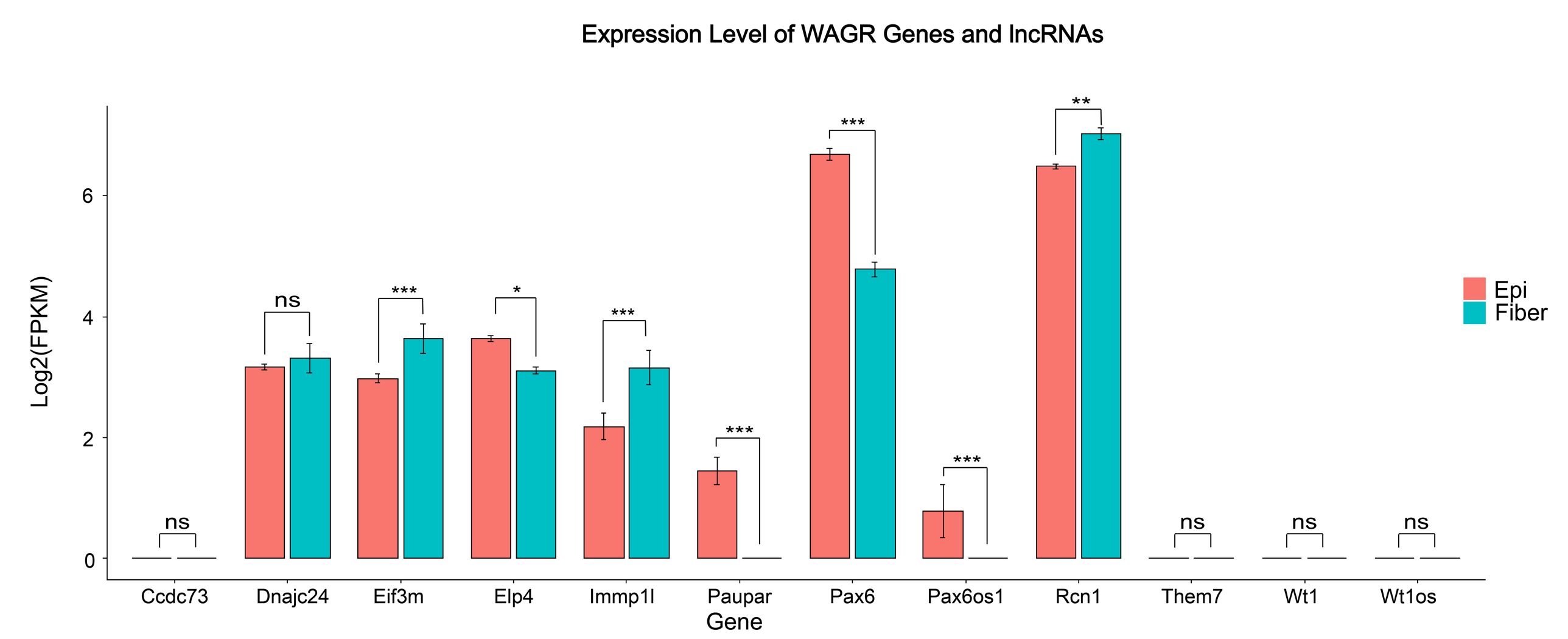


**Fig. S4: Expression levels of WAGR genes and lncRNAs.**

Expression data shown are for genes and lncRNAs within the ~1.1 Mb region (chr2:104879458-106021575) corresponding to human WAGR syndrome locus determined by RNA-seq in P0.5 lens epithelium and fiber cells [64]. Pax6 candidate auto-regulatory lncRNAs, Paupar and Pax6os1 [158, 159], show a reduction of expression to near zero. Ccdc73, Them7, Wt1, and Wt1os expression levels are nearly absent in lens cells. For transcriptional data, see Additional File 8: Table S3 and Additional File 9: Table S4. Significance bars denoted with asterisks: p ≤ 0.05, p ≤ 0.01 and p ≤ 0.001 denoted as *, **, *** respectively.


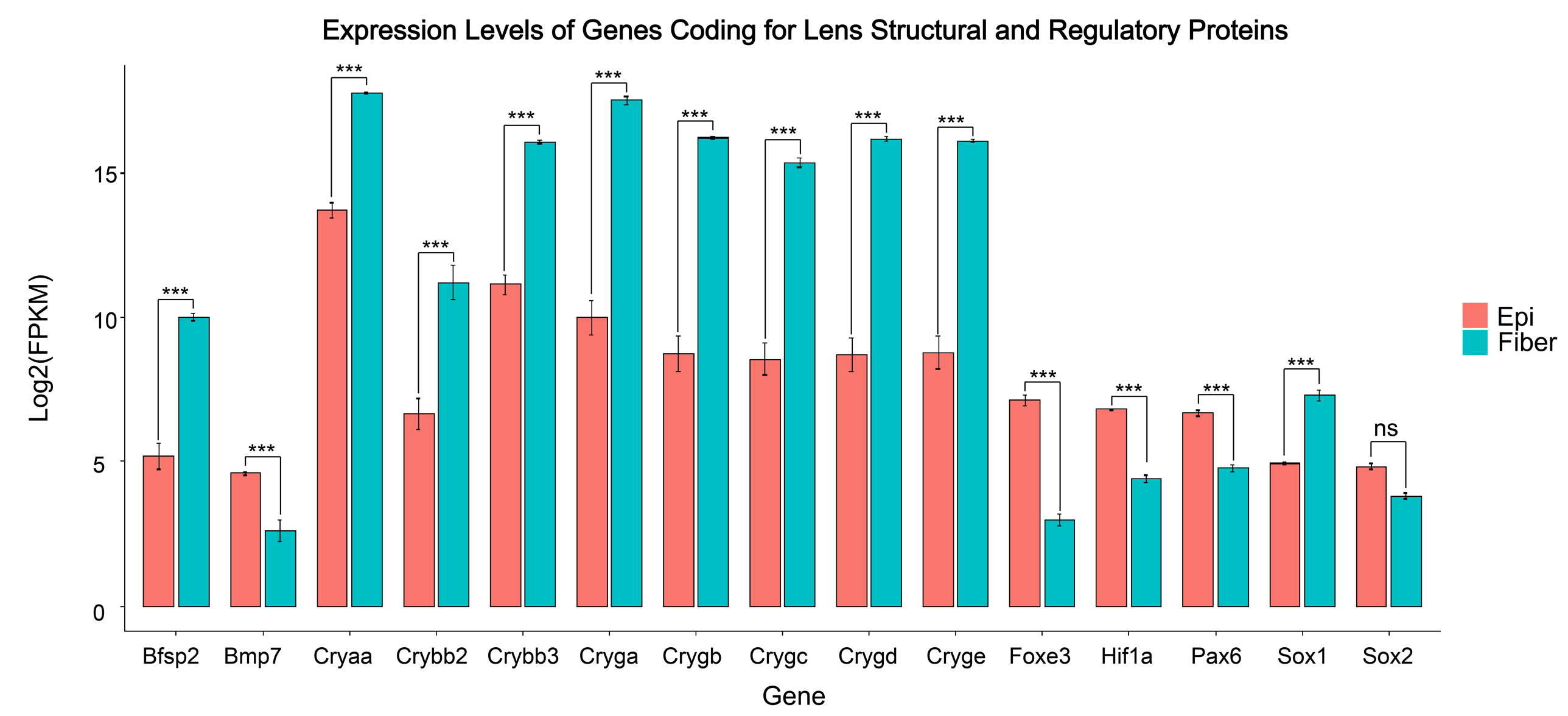


**Fig. S5: Gene expression levels encoding lens structural and regulatory proteins.**

Gene expression data (RNA-seq) of selected lens structural and regulatory proteins in lens epithelium and fiber cells of. Lens fiber cells show significant increase in expression of crystallin genes, all being expressed log2(FPKM) > 14. Genes encoding lens transcription factors Foxe3, Bmp7, Hif1a and Pax6 show a significant decrease in expression in lens fiber cells. These expression patterns show a shift in transcriptional output from regulatory proteins to key lens structural proteins during lens fiber cell differentiation. For transcriptional data, refer to Additional File 8: Table S3 and Additional File 9: Table S4. Significance bars denoted with asterisks: p ≤ 0.05, p ≤ 0.01 and p ≤ 0.001 denoted as *, **, *** respectively.
